# Deep learning-enabled 3D multimodal fusion of cone-beam CT and intraoral mesh scans for clinically applicable tooth-bone reconstruction

**DOI:** 10.1016/j.patter.2023.100825

**Published:** 2023-08-15

**Authors:** Jiaxiang Liu, Jin Hao, Hangzheng Lin, Wei Pan, Jianfei Yang, Yang Feng, Gaoang Wang, Jin Li, Zuolin Jin, Zhihe Zhao, Zuozhu Liu

**Affiliations:** 1 Stomatology Hospital, School of Stomatology, Zhejiang University School of Medicine, Zhejiang Provincial Clinical Research Center for Oral Diseases, Hangzhou 310000, China; 2 Zhejiang University-University of Illinois at Urbana-Champaign Institute, Zhejiang University, Haining 314400, China; 3 State Key Laboratory of Oral Diseases & National Center for Stomatology & National Clinical Research Center for Oral Diseases & West China Hospital of Stomatology, Sichuan University, Chengdu 610041, China; 4 College of Computer Science and Technology, Zhejiang University, Hangzhou 310058, China; 5 Harvard School of Dental Medicine, Harvard University, Boston, MA 02115, USA; 6 OPT Machine Vision Tech Co., Ltd., Tokyo 135-0064, Japan; 7 School of Electrical and Electronic Engineering, Nanyang Technological University, Singapore 639798, Singapore; 8 Angelalign Inc., Shanghai 200433, China; 9 Department of Stomatology, The First Affiliated Hospital of Shenzhen University, Shenzhen Second People’s Hospital, Shenzhen 518025, China; 10 Department of Orthodontics, School of Stomatology, Air Force Medical University, Xi’an 710032, China

**Keywords:** 3D tooth-bone reconstruction, digital dentistry, medical image analysis, cone-beam segmentation, intraoral scan tooth segmentation

## Abstract

High-fidelity three-dimensional (3D) models of tooth-bone structures are valuable for virtual dental treatment planning; however, they require integrating data from cone-beam computed tomography (CBCT) and intraoral scans (IOS) using methods that are either error-prone or time-consuming. Hence, this study presents Deep Dental Multimodal Fusion (DDMF), an automatic multimodal framework that reconstructs 3D tooth-bone structures using CBCT and IOS. Specifically, the DDMF framework comprises CBCT and IOS segmentation modules as well as a multimodal reconstruction module with novel pixel representation learning architectures, prior knowledge-guided losses, and geometry-based 3D fusion techniques. Experiments on real-world large-scale datasets revealed that DDMF achieved superior segmentation performance on CBCT and IOS, achieving a 0.17 mm average symmetric surface distance (ASSD) for 3D fusion with a substantial processing time reduction. Additionally, clinical applicability studies have demonstrated DDMF’s potential for accurately simulating tooth-bone structures throughout the orthodontic treatment process.

## Highlights

• Identified CBCT and IOS fusion for 3D tooth-bone reconstruction in clinical applications
• Proposed deep multimodal fusion pipeline to integrate both CBCT and IOS modalities
• Proposed novel techniques for accurate CBCT and IOS segmentation and 3D mesh fusion
• Collected multimodal dataset, validated method, demonstrated real-world applicability

## The bigger picture

The integration of digital dentistry with artificial intelligence holds significant importance. In the field of oral healthcare, various modalities of data provide complementary information. However, the manual fusion process required by dentists often consumes a significant amount of time, hindering precise reconstruction, diagnosis, and treatment simulation. To address this issue, we propose a multimodal fusion system that automates the fusion of diverse three-dimensional dental medical imaging modalities, enabling efficient and accurate tooth crown-root-bone reconstruction and analysis, thus serving as a powerful tool for oral healthcare practitioners. The application of this system holds paramount clinical significance, enhancing diagnostic accuracy, reducing treatment costs, and improving overall oral health outcomes for patients.

## Teaser

The DDMF framework offers accurate and efficient tooth-bone analysis in virtual dental treatment planning. Dentists benefit from improved segmentation and reconstruction of crown-root-bone structures. The study showcases the potential of deep learning in digital dentistry and inspires innovations in medical imaging. Overall, it advances dental healthcare by enhancing treatment planning and decision making, saving time for dental professionals and improving patient outcomes.

## Introduction

Digital technology is expected to change every aspect of modern dentistry, from virtual treatment planning to remote patient management. In particular, digital cone-beam computed tomography (CBCT) models have accelerated treatment planning and management in dental practice such as orthodontics and implant surgery. One advantage of these CBCT models is that they can illustrate the complex anatomical structures of both teeth and bones, creating a virtual model of the head.^1^^,^^2^ Recent advances in deep-learning-based automatic segmentation methods have enabled the segmentation of tooth-bone structures in CBCT images^3^^,^^4^^,^^5^^,^^6^^,^^7^ for digital model visualization. However, the clinical applicability of the segmented CBCT images remains limited. Previous research has shown that CBCT measurements can be up to 6.9% smaller than the actual values of objects, depending on the resolutions.^8^^,^^9^ This CBCT value discrepancy, termed shrinkage, makes the digital model unreliable for the precise simulation of implant surgery and orthodontic outcomes, suggesting the necessity of data matching between CBCT and other modality measurements in clinical practice. In addition, the accurate capture of occlusal surface information is of high importance in many clinical applications, as the design of saw, drilling, and orthognathic positioning guides is primarily dependent on occlusal surfaces.^10^^,^^11^^,^^12^ Nevertheless, CBCT often fails to recapitulate accurate information on occlusal surfaces because of the high density of enamel, dental restorations, implants, and orthodontic appliances.^13^^,^^14^^,^^15^ Furthermore, the correct anatomical position of dentition in the maxilla and mandible is often missing from CBCT images, making orthodontic treatment simulation impossible using CBCT alone.

Compared with CBCT, intraoral scanners (IOSs) are extensively used in dentistry to generate a digital impression of the tooth’s anatomy by projecting a light source onto the dental arches. An IOS scan is typically represented as a mesh with 150,000–350,000 triangular faces, each denoting a specific tooth or the gingiva. Recently, several deep-learning-based methods have been proposed for the automatic segmentation of IOS meshes.^16^^,^^17^^,^^18^^,^^19^^,^^20^ Although IOS meshes only provide information on the tooth crown and gingiva, their shrinkage ratio can be as low as 0.9%, making them the most accurate models in three-dimensional (3D) digital modalities. In addition, IOSs are sufficiently accurate for capturing occlusal information and dentition position.^21^ In practice, the fusion of both CBCT and IOS, that is, integrating comprehensive 3D CBCT models (complete tooth-bone structures) with high-resolution tooth crowns and accurate occlusal information from IOS, could provide crown-root-bone structures with the accuracy required for many dental applications.

Previous studies have focused on the fusion of CBCT and IOS for clinical applications.^22^^,^^23^ However, there are still many challenges in the fusion of CBCT and IOS, and a fully automatic, efficient, and clinically applicable solution has yet to be developed. First, the accurate segmentation of CBCT and IOS, which represents an indispensable prerequisite in current manual fusion solutions, is a non-trivial task because of the complicated anatomic and morphological features of different patients. Although recent research has highlighted the effectiveness of deep-learning methods for the accurate and automatic segmentation of CBCT and IOS, their performance could be further improved for clinically applicable tooth identification and multimodal fusion (MF). Second, the automatic fusion of both modalities remains an open and poorly defined task. Specifically, although the IOS provides clear half-jaw tooth crowns and occlusal information, CBCT models usually consider different bite positions without available separated jaws. Hence, the contacts in the maxilla and mandible, connected boundaries between adjacent teeth, and shape variance between reconstructed 3D CBCT models and IOS scans present great difficulties for an accurate and efficient MF and the delineation of individual teeth. Finally, the lack of a large-scale, real-world dataset for such MF tasks, which is crucial for the careful and systematic testing of these types of data fusion methods, represents another significant challenge.

**Figure 1:**
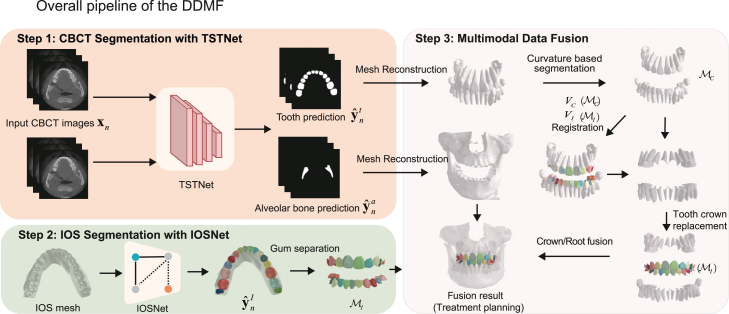
Overall pipeline of the DDMF framework Step 1: the pipeline of CBCT image segmentation for tooth and alveolar bone with TSTNet. Step 2: the pipeline of IOS segmentation with IOSNet and post-processing to generate FDI tooth codes. Step 3: the pipeline of the multimodal fusion module that fuses the reconstructed CBCT 3D meshes and IOS meshes with point curvature feature-based half-jaw segmentation, registration, and 3D fusion methods.

Therefore, in this study, we aimed to resolve the aforementioned challenges by introducing a fully automatic system that efficiently generates accurate crown-root-bone structures by fusing IOS mesh and CBCT image data with deep-learning methods. To achieve this, our model was trained on a large-scale dataset with 503 CBCT and 28,559 IOS meshes, manually annotated by human experts. For CBCT segmentation, our framework achieved an average Dice coefficient of 93.99%, significantly outperforming the baselines. For IOS segmentation, using a test dataset of 200 IOS meshes, our model achieved mean intersection over union (mIoU) values of 93.07% and 95.70% for the maxilla and mandible, outperforming state-of-the-art methods by 1.77% and 3.52%, respectively. For MF, Deep Dental MF (DDMF) showed a 0.47 mm Hausdorff distance (HD)^24^ between the model and ground truth for the entire set of teeth from 20 cases, which was 0.21 mm lower than that of CBCT reconstruction. The pipeline of the DDMF can be observed in Figure 1. In addition, the DDMF framework required 20–25 min to generate the fused model, compared with the duration of over 5 h required by human experts assisted by semi-automatic tools.

## Results

### Participants

We conducted experiments on a large-scale, real-world dataset that contained 503 samples with both CBCT and IOS, and an extra dataset of 28,559 IOS meshes collected from hospitals and clinics in 25 provinces in China from 2018 to 2021. The 503 patients all had malocclusion and aged between 9 and 48 years, with 32.5% being males and 67.5% being females, respectively. The CBCT data were acquired using different types of equipment, with resolutions ranging from 0.125 to 0.5 mm. The CBCT images and IOS meshes were annotated by a group of human experts.

### CBCT segmentation results

**Figure 2:**
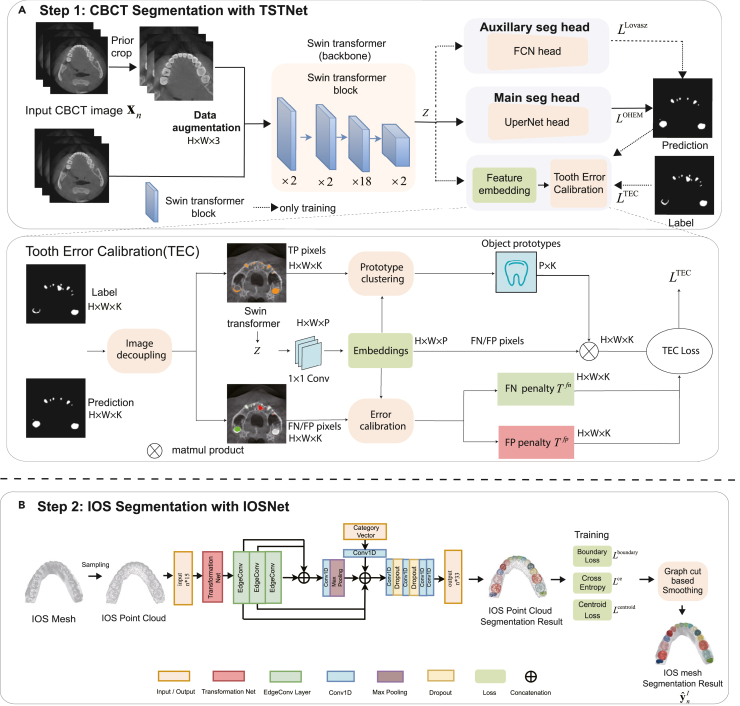
The pipeline of TSTNet for tooth and alveolar bone with TSTNet and the pipeline IOSNet (A) The pipeline of CBCT image segmentation for tooth and alveolar bone with TSTNet. (B) The pipeline of IOS segmentation with IOSNet and post-processing to generate FDI tooth codes.

We conducted a 5-fold cross-validation test, each with a hold-out set of CBCT images from 50 patients, to evaluate the segmentation performance of the proposed CBCT segmentation module, termed Tooth Swin Transformer Network (TSTNet), as shown in Figure 2. To establish a robust baseline, we compared TSTNet with several widely used and state-of-the-art segmentation networks as baselines and fine-tuned them for the best performance, such as UNet,^25^ UNet++,^26^ Deeplabv3,^27^ FCN,^28^ Medical Transformer (MedT),^29^ UCTransNet,^30^ and the standard Swin Transformer.^31^ We did not conduct comparisons with baselines that required 3D voxel annotations^7^^,^^32^ as the task definition was different and their codes and datasets were not publicly available. The Swin, MedT, UCTransNet, UNet, and UNet++ networks were implemented based on their original source codes, while the implementations of FCN and Deeplabv3 were based on the codes from MMsegmentation.^33^ To ensure that our evaluation accurately reflected the CBCT segmentation performance, we restored the original resolutions of the CBCT data for the 5-fold test.

**Figure 3:**
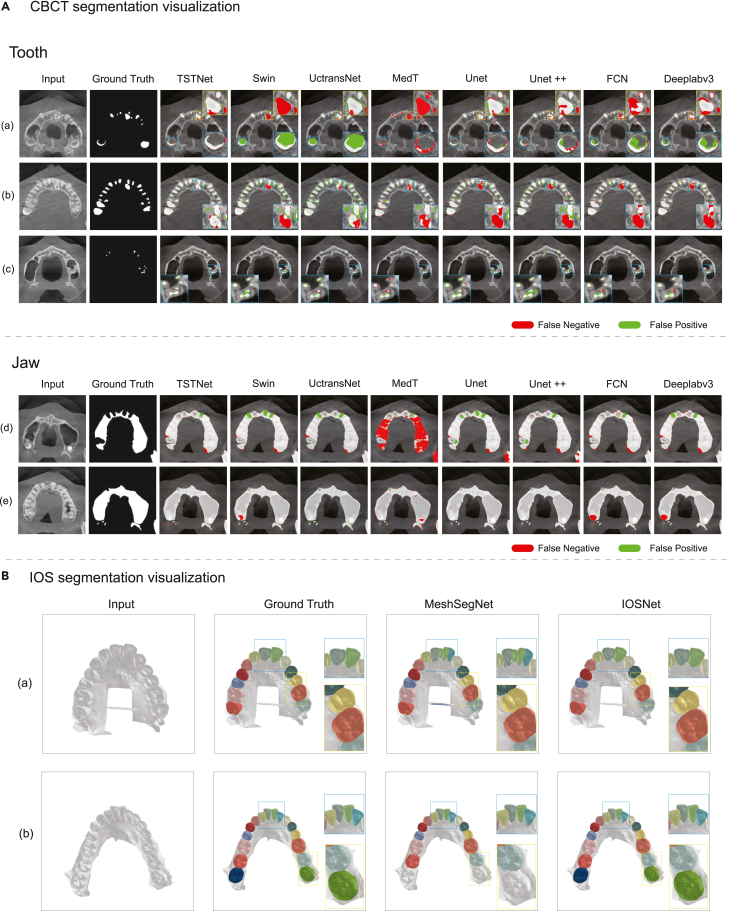
Visualization of the segmentation results for CBCT images and IOS meshes (A) Five cases for tooth and alveolar bone segmentation, demonstrating that our method commits fewer mistakes with false positives and false negatives. (B) Two cases for IOS tooth segmentation, demonstrating that our method performs much better than the state-of-the-art baselines.

**Figure 4:**
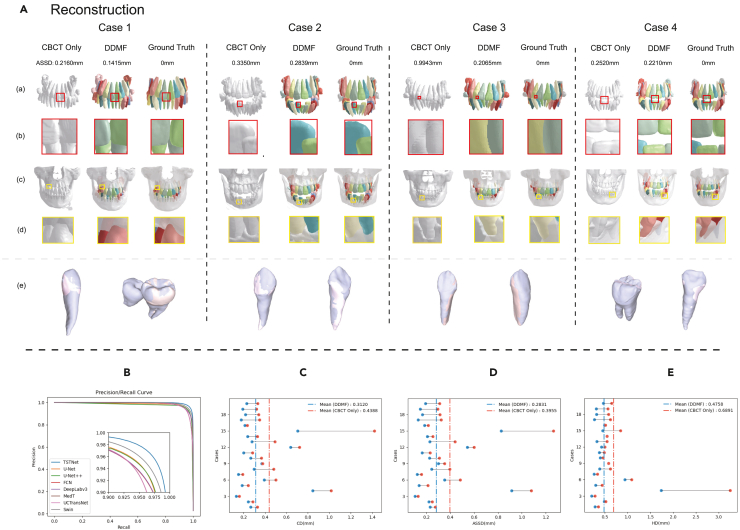
Visualization of the reconstruction and statistical results (A) Reconstructed 3D CBCT meshes and the fused 3D tooth and alveolar bone of DDMF and ground truth. (Ae) shows very tiny surface deviations as indicated by the light pink colored areas, which were consistent with the small ASSD and CD errors. (B) Statistical results of the TSTNet. (C–E), Statistical multimodal fusion results of DDMF.

The overall segmentation results are listed in Table 1. TSTNet achieved an average Dice coefficient and IoU of 93.99% and 88.68% on the 5-fold test, respectively, outperforming all the baselines. We noticed that a well-tuned UNet or UNet++ could already achieve a relatively good performance on this two-dimensional (2D) tooth segmentation task, and the standard Swin Transformer further boosted the performance. In addition, TSTNet consistently outperformed the state-of-the-art Swin model, with a performance gain of 1.05% IoU and 0.70% Dice coefficient. Considering the similar performance across all baselines, the performance gain was sufficiently significant, as illustrated in Figure 3. Moreover, as shown in Figure 4B, the precision/recall curve of TSTNet with the baselines indicated that TSTNet exhibited the best performance. Additional results are shown in Tables S1 and S5.

**Table 1:** Five-fold cross-validation segmentation results on CBCT (each tested with 50 patients)

Model	IoU, %	Dice coefficient, %	Recall, %	Precision, %
UNet	86.90 ± 1.06	92.95 ± 0.61	92.83 ± 2.74	93.23 ± 1.57
UNet++	86.83 ± 1.16	92.95 ± 0.66	93.40 ± 1.66	92.51 ± 0.60
FCN	85.68 ± 1.60	92.28 ± 0.92	92.21 ± 2.31	92.39 ± 0.48
Deeplabv3	85.68 ± 1.61	92.28 ± 0.93	92.05 ± 2.34	92.54 ± 0.53
MedT	73.39 ± 5.97	84.52 ± 3.81	75.00 ± 5.87	97.01 ± 1.01^∗^
UCTransNet	84.08 ± 0.80	91.35 ± 0.47	97.95 ± 0.62^∗^	85.59 ± 0.73
Swin	87.61 ± 1.19^#^	93.29 ± 0.67^#^	93.95 ± 1.68^#^	92.95 ± 0.54
TSTNet	88.68 ± 2.15^∗^	93.99 ± 1.20^∗^	92.37 ± 2.78	95.71 ± 0.70^#^

IoU, intersection over union. ^∗^Best performance and ^#^Second-Best performance. Values denote mean ± SD on cross-validation.

The superior performance of TSTNet is also convincingly demonstrated by the visualizations shown in Figure 3A. Our TSTNet method showed fewer false positive (FP) and false negative (FN) errors than the baselines. For instance, the baselines might incorrectly recognize a large portion of the background as erupted third molars (case a), fail to identify the large incisor (case a), fail to recognize hyperdontia (case b), or fail to distinguish the ambiguous boundaries among tiny tooth slices (case c). By contrast, TSTNet generated considerably better results for these complex cases. A similar performance could also be observed in the jaw segmentation results, as in cases d and e. Further visualizations can be found in Figures S3, S4, and S7.

### IOS segmentation results

The performance was also compared with classical point cloud segmentation networks (i.e., PointNet,^34^ PointNet++,^35^ and DGCNN^36^) and strong IOS segmentation CNN baselines in Xu et al.^17^ (i.e., MeshSegNet,^37^ DCNet,^16^ and TSGCNet^38^), all following their original settings and source codes. We evaluated the performance of IOSNet on the same test set as in Hao et al.^16^ with 200 meshes from 100 patients. We reported the mIoU, per-face accuracy, and average-area accuracy as in Hao et al.^16^ for comparison. Additional visualizations are shown in Figure S5.

The results are reported in Table 2. The IOSNet achieved an mIoU of 93.70% and 95.70% on maxillary and mandibular IOS scans, respectively, outperforming the baselines by 1.77% and 3.52%. Figure 3B illustrates two cases. The results demonstrated that IOSNet could generate segmentations nearly identical to the ground truth; that is, it did not recognize two incisors as one tooth (case a) and could recognize the third molar and generate better tooth-gingiva and tooth-tooth boundaries (cases a and b). Given that DCNet could generate clinically applicable results for most cases,^16^ such a significant improvement in mIoU could corroborate the excellent performance of IOSNet. We do not provide too many visualizations here. The performance of IOSNet was subsequently demonstrated through real-world clinical validation using our DDMF framework. Additional results are reported in Table S2.

**Table 2:** Segmentation results of the IOSNet and baselines (tested on 100 patients)

Model	Mandible			Maxilla		
	mIoU, %	ACC_f_, %	ACC_a_, %	mIoU, %	ACC_f_, %	ACC_a_, %
CNN	85.32 (81.96–88.69)	91.74 (89.77–93.72)	93.75 (92.14–95.36)	89.68 (86.90–92.48)	94.14 (92.35–95.93)	95.80 (94.37–97.22)
PointNet	63.10 (57.90–69.29)	79.67 (76.32–83.01)	86.21 (83.93–88.49)	59.05 (53.14–64.95)	75.58 (71.63–79.53)	80.95 (77.67–84.24)
PointNet++	83.22 (81.38–85.06)	91.49 (90.41–92.57)	94.59 (93.91–95.27)	85.82 (84.14–87.50)	93.15 (91.92–94.38)	95.65 (94.95–96.35)
DGCNN	84.93 (83.27–86.58)	92.74 (91.82–93.65)	95.80 (95.18–96.43)	88.70 (87.55–89.86)	94.41 (93.73–95.10)	96.94 (96.42–97.45)
MeshSegNet	82.82 (80.51–85.13)	93.50 (92.55–94.45)	95.04 (94.18–95.90)	85.62 (83.61–87.64)	93.96 (93.04–94.88)	95.59 (94.77–96.41)
TSGCNet	83.04 (80.34–85.96)	92.92 (91.57–94.37)	94.60 (93.43–95.89)	80.31 (77.61–83.32)	91.53 (90.07–93.01)	93.55 (92.26–94.87)
DCNet	91.93 (91.09–92.78)	96.01 (95.42–96.61)	97.98 (97.56–98.41)	92.18 (91.02–93.36)	95.99 (95.28–96.71)	97.90 (97.33–98.45)
IOSNet	90.37 (88.69–92.06)	96.82 (96.44–97.20)	97.96 (97.61–98.31)	92.50 (91.44–93.57)	97.11 (96.79–97.44)	98.27 (97.97–98.57)
IOSNet^a^	92.44 (91.50–93.37)	97.31 (97.05–97.57)	98.41 (98.18–98.64)	93.73 (93.21–94.25)	97.36 (97.10–97.62)	98.53 (98.32–98.74)
IOSNet^b^	93.70 (92.15–95.25)^∗^	98.14 (97.78–98.50)^∗^	98.81 (98.49–99.14)^∗^	95.70 (94.80–96.60)^∗^	98.35 (98.08–98.61)^∗^	99.05 (98.82–99.28)^∗^

mIoU, mean intersection over union; ACC_f_, per-face accuracy; ACC_a_, average-area accuracy. Values in parentheses are 95% confidence intervals. All baselines and IOSNet are trained on a small dataset with 4,271 IOS scans. ^∗^Best performance.

^a^IOSNet trained on 28,000 scans without smoothing.

^b^IOSNet trained on 28,000 scans with smoothing.

### Multimodal fusion results

To evaluate the MF achieved by our DDMF framework, we computed the average symmetric surface distance (ASSD),^24^ HD,^24^ and Chamfer distance (CD) metrics^39^ between the fused outputs and ground truth for 50 patients. Notably, the ground truth fused models were provided by a committee of human experts and were ensured to be clinically applicable because they were successfully used in clinical practice. Table 3 reports the fusion error for seven different teeth categories as well as the overall fusion performance. We excluded third molars because many patients did not have third molars in our MF dataset. The results of these intermediate steps are described in the following sections. We did not investigate any baselines here as there are no systematic solutions for this challenging task. However, we conducted a clinical applicability study to test the effectiveness of our method.

**Table 3:** Multimodal fusion results of the proposed method (tested on 1,003 teeth in 50 cases)

	Central incisor	Lateral incisor	Cuspid	1st premolar	2nd premolar	1st molar	2nd molar	Average
ASSD, mm	0.16 ± 0.06	0.17 ± 0.06	0.18 ± 0.08	0.17 ± 0.08	0.17 ± 0.07	0.19 ± 0.08	0.21 ± 0.06	0.17 ± 0.07
CD, mm	0.19 ± 0.06	0.19 ± 0.06	0.21 ± 0.07	0.20 ± 0.07	0.19 ± 0.06	0.21 ± 0.07	0.24 ± 0.06	0.20 ± 0.06
HD, mm	0.36 ± 0.15	0.36 ± 0.15	0.38 ± 0.23	0.36 ± 0.15	0.37 ± 0.16	0.41 ± 0.07	0.47 ± 0.15	0.37 ± 0.17

Data are expressed as mean ± SD. ASSD, average symmetric surface distance; CD, Chamfer distance; HD, Hausdorff distance.

We noticed that the corresponding average distances could be as small as 0.17, 0.20, and 0.37 mm for ASSD, CD, and HD, respectively with consistently small SDs. The errors were small and consistent across different teeth categories. The relatively large errors in the 1st and 2nd molars were due mainly to these two molars’ having larger volumes. Nonetheless, considering that the resolution of CBCT machines ranged between 0.125 and 0.5 mm, a distance error as low as one to two pixels would be acceptable for numerous clinical applications, as validated by human experts and demonstrated by the clinical applicability test. More detailed MF results are presented in Tables S3 and S4.

In this regard, four cases of MF are visualized in Figure 4, with additional visualizations included in Figure S6. By fusing CBCT and IOS, instance-level 3D teeth could be automatically generated by incorporating the Fédération Dentaire Internationale (FDI) tooth number information from the IOS, as shown in Figure 4A, thus eliminating the need for performing the time-consuming task of providing instance-level annotations in CBCT slices. As shown in Figures 4A and 4B, the magnified tooth-tooth boundaries also reveal the existence of tooth adhesion in the CBCT reconstructions, while the fused results were nearly identical to the ground truth. Furthermore, we visualized both teeth and bones and examined the tooth-bone relationships in each case. As shown in Figures 4A–4D, our findings demonstrated that DDMF could accurately identify critical tooth-bone boundaries such as the relative positions between the tooth roots, and alveolar bones were clearly recognized in all cases. Hence, these results can be employed for high-fidelity 3D simulations of treatment plans. Finally, we randomly selected several teeth and placed the fused results and ground truth in the same position with the same orientation to identify the differences. From Figures 4A–4E, there were very small surface deviations, as indicated by the light pink areas, which were consistent with the small ASSD and CD errors. Furthermore, CD, ASSD, and HD were calculated for the entire set of teeth from the 20 cases to compare the variations between CBCT reconstructions and DDMF outcomes. Figures 4C–4E display the statistical findings. It can be observed that the DDMF reduced the prediction error, thus demonstrating its effectiveness.

### Ablation on the effectiveness of data augmentation, TEC, and loss

An ablation study was conducted to evaluate the effectiveness of the proposed TSTNet. The Swin Transformer was used as the baseline and only achieved an IoU of 90.52%, on a development set of 43 patients. In contrast, our TSTNet achieved an IoU of 93.22%, while each novel component, including prior-knowledge-based data augmentation, the TEC component, and the integrated loss function, significantly contributed to the enhanced performance, as illustrated in Table 4.

**Table 4:** Ablation studies of TSTNet (tested on 43 patients)

DA	Loss	TEC	IoU, %	Dice, %	Recall, %	Precision, %
✓	✓	✓	93.22**^∗^**	96.49^∗^	97.75	95.27^∗^
–	✓	✓	91.46	95.54	96.58	94.52
✓	–	✓	92.97	96.35	97.93^∗^	94.92
✓	✓	–	92.87	96.30	97.92	94.73
–	–	–	90.52	95.02	97.69	92.50

DA, data preprocessing and augmentation. ^∗^Best performance.

### Ablation on the effectiveness of point curvature feature

We also discuss the effectiveness of the proposed curvature-based algorithm for separating the upper and lower jaws, with experiments conducted on 50 patients, 31 of whom were in a closed-biting position with tight tooth contacts. Remarkably, our method successfully separated 94% of the upper and lower jaws, whereas traditional segmentation algorithms based on the Gaussian curvature and DBSCAN^40^ separated only 42% and 24% of them, respectively, as shown in Table 5.

**Table 5:** Curvature-based segmentation on 3D CBCT meshes (tested on 50 patients)

	Gaussian curvature-based method	DBSCAN	This study
Success rate	42%	24%	94%

Success rate is the probability of successfully separating CBCT mesh into the maxilla and mandible.

### Clinical utility evaluation

We assessed the clinical utility of our method by computing the end-to-end inference time of each module and the DDMF framework (see Tables 6 and S1). TSTNet took approximately 0.052 and 3 s with multiscale post-processing to segment one CBCT slice, three to four orders of magnitude faster than human experts using interactive software. IOSNet took approximately 24 s to segment a half-jaw IOS, which was approximately 50 times faster than that of human experts. The DDMF framework took 20–25 min to generate the fused 3D mesh model following the sequential processing order, which took at least 5 h for experienced human experts, even with the help of interactive software and semi-automatic algorithms, as reported in Table 6. The current inference speed is appealing to dentists. It is worth noting that in our current framework, CBCT slices were segmented one by one, followed by IOS segmentation before fusion. This inference process can be substantially accelerated by processing the CBCT slices in parallel or by segmenting the IOS and CBCT simultaneously.

**Table 6:** End-to-end model inference time and clinical utility of DDMF

Models	DDMF	Human experts
Inf-T, min	∼20–25	∼300–400

Inf-T, end-to-end inference time.

## Discussion

### CBCT segmentation

Deep convolutional neural networks (CNNs) have shown promising performance in a variety of medical image segmentation tasks, such as multiorgan, cardiac, and lesion segmentation.^25^^,^^41^^,^^42^^,^^43^^,^^44^ Recently, inspired by the success of transformers in natural language processing,^45^^,^^46^^,^^47^ transformers have been introduced into the computer vision domain to learn explicit global and long-range semantic information interactions, demonstrating superior performance on many downstream vision tasks.^31^^,^^48^^,^^49^^,^^50^ Previous studies have also explored employing transformers for medical image analysis^3^^,^^29^^,^^30^^,^^41^^,^^42^ and obtained competitive or better performance than CNNs. In this study, we proposed TSTNet, a novel approach based on the Swin Transformer backbone with additional domain-specific designs for CBCT segmentation.

Although the automatic segmentation of CBCT has been established by many groups, the automatic segmentation of the tooth and alveolar bone is a challenging task, as teeth exhibit large geometric variations, similar intensities between the tooth and alveolar bone, and complex topological and anatomical structures across different patients.^3^^,^^4^^,^^6^^,^^7^^,^^32^^,^^51^ Prior works can be divided into two categories: (1) traditional methods based on hand-crafted features^51^^,^^52^ and (2) deep-learning-based methods which achieve better performance than traditional methods.^3^^,^^5^^,^^7^^,^^31^^,^^52^^,^^53^

Among deep-learning-based methods, formulated tooth segmentation is an instance segmentation task on 3D CBCT images that usually requires annotating tooth instances on 3D voxels across the entire CBCT scan.^7^^,^^32^ However, annotating 3D voxels in CBCT scans is costly and time-consuming, compared with annotating some selected 2D slices.^54^ Additionally, general 3D segmentation methods typically require more computational resources and exhibit a larger inference latency.^54^

Meanwhile, some 3D works only focused on tooth segmentation and ignored the alveolar bones. In contrast, we simultaneously recognized 3D teeth and alveolar bones with semantic segmentation over 2D slices. TSTNet only requires annotating tooth pixels on several 2D slices, that is, approximately 20 slices from 300–600 slices in a CBCT scan. Additionally, generating FDI tooth codes is also critical for clinical applications. Jang et al.^4^ designed a tooth identification method based on 2D panoramic images reconstructed from CBCT to identify incisors, canines, premolar, and molars, then assigning FDI tooth codes accordingly. However, the two-stage classification procedure is error-prone and cannot handle complicated cases, such as missing teeth or hyperdontia because the tooth numbers are not inherently provided. Meanwhile, in the proposed DDMF framework, we identify the FDI tooth number with the help of the IOS during fusion. By doing so, we substantially reduce the human labor required for annotation as well as the network complexity and the corresponding training efforts for segmentation. For example, hierarchical or multistage networks are not needed.^4^^,^^5^^,^^7^ Finally, none of the existing methods have been applied in the context of the MF of CBCT and IOS^32^; hence, their clinical applicability remains limited in practice.

### IOS segmentation

IOS segmentation is closely related to 3D point cloud or mesh segmentation in computer vision, in which many methods have been proposed under different settings (e.g., PointNet and DGCNN). For 3D tooth segmentation on IOS, traditional methods use handcrafted geometrical features and semi-automatic segmentation methods.^55^^,^^56^^,^^57^^,^^58^^,^^59^^,^^60^

Recent studies have employed deep learning for automatic 3D tooth segmentation,^16^^,^^17^^,^^18^^,^^19^^,^^20^^,^^59^^,^^61^ in which deep neural networks are used to conduct segmentation either on mesh or point clouds. The performance has been further boosted by methods that design specific neural network architectures for end-to-end tooth segmentation, such as MeshSegNet,^37^^,^^62^ DCNet,^16^ TSGCNet,^38^ and Mask-MCNet.^63^ However, most of these methods have been only trained and evaluated on very limited datasets, that is, datasets with 30–120 IOS scans,^16^ which does not cover complex real-world cases, for example, without third molars or heterogeneous oral diseases. Hence, their applicability to heterogeneous real-world cases has not yet been verified. Recently, DCNet was the first network to be verified using a clinical applicability study and was demonstrated to generate clinically applicable results in many real-world clinical cases.^16^ In comparison, our proposed IOSNet outperformed DCNet with novel loss functions on a much larger dataset. Such state-of-the-art performance corroborates its clinical applicability for segmentation in most cases.

### CBCT and IOS fusion

Very few studies have been conducted on the MF of CBCT and IOS. Previous studies have relied on traditional registration and level-set segmentation methods and required considerable manual work to crop and stitch the reconstructed meshes.^23^ Moreover, they required many heuristics, similar to a manual visualization system, rather than producing high-fidelity fused outputs on the basis of CBCT and IOS scans. Hence, the segmentation performance and stitched results were based only on several illustrations and were not systematically evaluated. Although some prior studies have investigated how to perform registration between CBCT and IOS scans, they did not identify the upper and lower jaws or required manual work to do so, and the instance-level tooth delineation and crown-root-bone analysis for real-world clinical use were not available as well.^4^^,^^64^^,^^65^^,^^66^ In contrast, our proposed DDMF framework introduces novel deep-learning-based methods for CBCT and IOS segmentation and achieves end-to-end fusion with a novel half-jaw registration and fusion strategy.

One of the major contributions of our work is the demonstration of its clinical applicability in collaboration with industrial partners and hospitals and clinics in China. Our system has been implemented in real-world clinics, enabling doctors to visualize the crown-root-bone relationships during the entire orthodontic treatment process and make better treatment decisions for patients. Notably, our system’s superiority in addressing the problems of root-bone relationships has been confirmed through the successful outcomes of real-world clinical cases, outperforming traditional treatment planning methods that rely solely on IOS or CBCT imaging, as illustrated in the Videos S1 and S2.

Video S1. Previous treatment planning outcome with IOS

Video S2. New treatment planning with DDMF framework

### Limitations

Although our DDMF framework demonstrated superior performance and clinical applicability, several limitations must be addressed in future studies. First, the DDMF involves several intermediate steps in the fusion process, such as curvature-based segmentation, two-stage registration, and 3D fusion. This results in the most significant errors in the DDMF framework, that is, the half-jaw separation and registration method might fail in 6%–10% of total cases based on our statistics, implying that manual correction is still required for these complicated cases. Our future work will involve designing novel learning-based methods for a more accurate half-jaw separation, 3D registration, and fusion. Second, the segmentation performance for CBCT and IOS scans can be further improved. Although TSTNet and IOSNet achieved superior performance even for patients with unerupted teeth, hyperdontia, malposition, and ambiguous tooth boundaries, they may still be error-prone for extremely complicated cases; for example, patients with metal artifacts or root canal therapy. Future studies will combine deep learning with domain knowledge in stomatology to achieve better segmentation results for both CBCT and IOS scans. Finally, although our method has already been integrated into clinical software to assist in orthodontic treatment planning, it has been mainly evaluated from an algorithmic perspective. Therefore, the clinical usage and dental clinical findings should be further explored using rigorous multicenter clinical trials and large-scale complicated cases.

### Conclusion

We proposed a novel DDMF framework of multimodal CBCT and IOS fusion for intelligent tooth crown-root-bone analyses. Our framework comprises a CBCT segmentation module, an IOS segmentation module, and a MF module. The effectiveness of each module and the entire framework was systematically demonstrated with comprehensive experiments on our large-scale multimodal dataset. Our framework had been integrated into a clinical software to assist dentists in orthodontic treatment planning and decision making. Future work includes better MF and segmentation algorithms and large-scale multicenter clinical trials.

## Experimental procedures

### Resource availability

#### Lead contact

The lead contact for questions about this paper is Zuozhu Liu, who can be reached at zuozhuliu@intl.zju.edu.cn.

#### Materials availability

This study did not generate new unique reagents.

### Ethics statement

The study was approved by the institutional ethics review boards of West China College of Stomatology Sichuan University (WCHSIRB-D-2021-331). The recruited patients of the retrospective and prospective parts were well informed of the study and all provided signed informed consent. Patients or the public were not involved in the design, recruitment, and conduct of the study.

### Data acquisition and study design

For CBCT segmentation, each pixel is associated with an annotation for background, tooth, or alveolar bone. Note that we did not annotate all slices in 503 CBCT scans (comprising more than 150,000 slices) but only annotated 9,651 CBCT slices of them for CBCT tooth segmentation, where we selected 15 to 25 slices for each patient based on the dentists’ heuristics. Most CBCT slices have a pixel resolution (height × width) ranging from 260× 260 to 1,000× 1,000. For IOS segmentation, each mesh face is associated with an annotation for the gingiva or the FDI notation of 32 different teeth. The 3D IOS mesh scans usually contain 100,000 to 400,000 triangular faces with a spatial resolution of 0.008–0.02mm. For 3D MF, each tooth is annotated by human experts assisted by CAD software to generate golden-standard 3D fused models. During annotation, a committee of experts were asked to decide whether the annotation is satisfactory for future oral diagnosis and treatment planning. The annotations were checked and improved by these experts until they met the necessary quality standards for future clinical use. Detailed annotation pipeline and data statistics are reported in the Supplementary.

### Methodology

The DDMF framework includes three major steps: the CBCT segmentation, the IOS segmentation, and the MF, as illustrated in Figure 1. First, the CBCT segmentation module takes 2D CBCT slices as input and generates predictions for all pixels. Second, the IOS segmentation module takes IOS meshes as input and generates predictions for all faces in the IOS meshes. The MF module then integrates the reconstructed CBCT meshes and segmented IOS meshes to generate fused, accurate, high-resolution meshes with high-fidelity crown-root-bone structures for real-world clinical applications.

#### CBCT segmentation with TSTNet

We formulated the CBCT segmentation task as 2D segmentation over each CBCT slice. Suppose we have a dataset $D={\left\{\left(x_{n},y_{n}^{t},y_{n}^{a}\right)\right\}}_{n=1}^{N}$, where $x_{n}$, $y_{n}^{t}$, and $y_{n}^{a}$ denote the nth CBCT slice, corresponding pixel-level tooth and alveolar bone labels respectively, and N is the number of images in the dataset. In this step, the goal is to obtain pixel-wise tooth predictions ${\hat{y}}_{n}^{t}$ and alveolar bone predictions ${\hat{y}}_{n}^{a}$ from the CBCT slice $x_{n}$, where the predictions can be used to reconstruct the 3D tooth and alveolar bone mesh models for subsequent usage. Below are step-by-step details of the TSTNet, as illustrated in Figure 2. More architecture details are in the Figure S1.

##### Data preprocessing

The number of background pixels and tooth pixels in the CBCT images $x_{i}$ are highly imbalanced. Based on empirical statistics from the annotated masks $y_{n}^{t}$, we cropped the lower 1/4 and right/top/left 1/10 in the original CBCT image $x_{n}$ to alleviate the pixel-wise class imbalance problem. Afterward, the CBCT images $x_{n}$ were augmented with resizing, random clip, and flip strategies.

##### The backbone of TSTNet

We proposed a deep-learning method based on Swin Transformer, named TSTNet, to perform 2D segmentation on each CBCT slice $x_{n}$. As shown in Figure 2, TSTNet is composed of a backbone network and multiple segmentation heads. Swin Transformer^31^ is utilized as the backbone, which is a hierarchical Transformer whose representation is computed with Shifted windows. The hierarchical architecture has the flexibility to model at various scales, capable of extracting both local and global features, where the local features help identify the boundary between the background class and the tooth class, and the global features provide richer context information for robust classification. Such designs allow the Swin Transformer to achieve superior performance on multiple vision tasks compared with CNN based methods. Specifically, the backbone of TSTNet contains four stages. Each stage is constructed using 2/2/18/2 Swin Transformer blocks, and the multi-head attention (MHA) modules within these blocks contain 4/18/16/32 heads respectively.^31^ We followed the original setting of Swin Transformer to employ the UperNet head as the main segmentation head for multiscale feature aggregation and fusion. More details about the backbone can be found in.^31^

##### Tooth error calibration head

Existing segmentation heads misclassified the pixels in the boundary between the tooth and alveolar bone. To alleviate this issue, we proposed the tooth error calibration (TEC) head to correct the error-prone feature representations in boundary areas for better segmentation performance. Notably, TEC can be incorporated into the hidden layers during training, and decoupled in the inference stage without additional parameters and inference time. TEC consists of the image decoupling, prototype clustering, and error calibration submodules, as illustrated in Figure 2.

The ground truth $y_{n}^{t}$ and the predicted tooth mask of the UperNet head serve as inputs for TEC. The image decoupling submodule categorizes the pixels into three sets based on predictions: true positive (TP) $s_{k}^{tp}$, FN $s_{k}^{fn}$, and FP $s_{k}^{fp}$ for category k, where k denotes the tooth category in our case. Based on the current and historical TP pixels, the prototype clustering submodule calculates the category prototype $\mu_{k}$ via exponential moving average (EMA)^69^:

(Equation 1) $$\mu_{k}=\rho \mu_{k}+\left(1-\rho \right)\frac{1}{n_{k}^{tp}}\sum_{i\in s_{k}^{tp}}e_{i}\text{,}$$

where $n_{k}^{tp}$ is the number of current TP pixels for category k, $e_{i}$ is the embedding for pixel i, ρ=0.9 is the momentum value to adjust the retained proportion of historical prototype. Afterward, the cosine similarity between pixel i and prototype $\mu_{k}$ in embedding space is defined as follows:

(Equation 2) $$\cos\;\theta_{ik}={\tilde{\mu }}_{k}{\tilde{e}}_{i}^{⊤}=\frac{\mu_{k}e_{i}^{⊤}}{{\left\|\mu_{k}\right\|}_{2}{\left\|e_{i}\right\|}_{2}}\text{,}$$

where ${\left\|\cdot \right\|}_{2}$ is the L2 distance and ${\tilde{\mu }}_{k}$ is the normalized vector with magnitude 1. Our method aims at maximizing $\cos\;\theta_{ik}$ to make $e_{i}$ close to prototype $\mu_{k}$, thus clustering pixels of the same category for better tooth segmentation performance.

The TEC simultaneously calculates the penalty terms for FN and FP pixels with an error calibration submodule. The FP errors occur when pixels of the background are excessively similar to the tooth pixels in the feature space, while the FN errors occur when pixels belong to the tooth misclassified as background. To tackle this, we take pixel i of the tooth category k as an anchor. The FN penalty term pulls the FN pixels toward the anchor of the tooth class, while the FP penalty term pushes the FP pixels to the opposite pole against the anchor in the embedding space, i.e., as background pixels. We define the FN and FP penalty terms as follows:

(Equation 3) $$T_{i}^{fp}=\left\{ \begin{array}{cc} 1+\frac{1}{n_{k}^{fp}}\sum_{j\in s_{k}^{fp}}{\tilde{e}}_{j}{\tilde{e}}_{i}^{⊤} & ,\text{if}\;n_{k}^{fp}>0\\ 0 & ,\text{otherwise.} \end{array}\right.\text{,}$$

(Equation 4) $$T_{i}^{fn}=\left\{ \begin{array}{cc} 1-\frac{1}{n_{k}^{fn}}\sum_{j\in s_{k}^{fn}}{\tilde{e}}_{j}{\tilde{e}}_{i}^{⊤} & ,\text{if}\;n_{k}^{fn}>0\\ 0 & ,\text{otherwise.} \end{array}\right.\;\text{.}$$

$T_{i}^{fp}$ is 0 when there are no FP pixels or the average similarity converges to −1, indicating all FP pixels are in the opposite direction from anchor i. $T_{i}^{fn}$ is reduced to 0 when there are no FN pixels or all FN and TP pixels are placed in the same direction in the feature space. Finally, TEC integrates the cosine similarities and the FP/FN penalty terms into a TEC loss, serving as an additional loss to train the network:

(Equation 5) $$L_{i}^{\text{TEC}}=-\log\frac{\exp\left(\cos\;\theta_{ik}/\tau -\left(1-p_{ik}\right)T_{i}^{fn}\right)}{\exp\left(\cos\;\theta_{ik}/\tau -\left(1-p_{ik}\right)T_{i}^{fn}\right)+\sum_{l\neq k}\exp\left(\cos\;\theta_{il}/\tau \right)}-\log\frac{\exp\left(\cos\;\theta_{ik}/\tau -\left(1-p_{ik}\right)T_{i}^{fp}\right)}{\exp\left(\cos\;\theta_{ik}/\tau -\left(1-p_{ik}\right)T_{i}^{fp}\right)+\sum_{l\neq k}\exp\left(\cos\;\theta_{il}/\tau \right)},$$

where τ=0.5 is the temperature, and $p_{ik}$ is the probability prediction for wrongly segmented pixels. By minimizing $L_{i}^{\text{TEC}}$, all FP pixels are pushed to the opposite direction against anchor i, and all FN pixels are pulled to the same direction toward anchor i.

##### Loss function of TSTNet

In CBCT slices, the area of the background class is roughly ten times larger than that of the tooth class, which leads to a severe class imbalance problem. To overcome this issue, the class balanced cross entropy loss and online hard example mining (OHEM) cross entropy loss^33^^,^^70^ are introduced as the objective function of the UperNet head, intending to avoid overfitting of the background class. The OHEM loss tends to choose the examples with higher loss or more diversity as the training data and assign different weights to different classes, reducing the bias to the class with majority samples.^71^ Besides the UperNet and TEC heads, TSTNet also employs an FCN head, serving as the auxiliary head to further improve the segmentation performance. As for the FCN head, the Lovasz-Softmax loss is employed as the objective function, which performs better on segmenting small objects and reducing FNs, and helps alleviate the class imbalance problem to some extent and avoid the missed detection of tooth area.^70^^,^^72^ Together with the OHEM cross-entropy loss and the pixel-level TEC representation learning modules with a novel TEC loss, TSTNet is able to attain fine-grained segmentation results across various tooth anatomies. Overall, the total loss for TSTNet on pixel i is defined as:

(Equation 6) $$L_{i}^{\text{Seg}}=L_{i}^{\text{OHEM}}+L_{i}^{\text{Lovasz}}+\lambda Li^{\text{TEC}},$$

where λ=0.1 is the weight of the TEC loss. During inference, the TEC and FCN heads are discarded.

#### IOS segmentation with IOSNet

We performed 3D segmentation on IOS meshes. The goal of this step is to predict the label $y_{n}^{I}$ given any face $f_{n}$ in the mesh $M_{I}$, where $y_{n}^{I}\in \left\{0,11-18,21-28,31-38,41-48\right\}$ denotes the gingiva and FDI notations for the 32 permanent teeth, respectively. We first transformed the IOS mesh to a point cloud during preprocessing by randomly sampling 10,000 face center points from each IOS mesh. For each point, we extracted the 3D coordinates, face normal vector, and face shape descriptor to form a 15-dimensional feature vector, as in.^16^ Then, the point clouds were segmented with the IOSNet and further mapped back to the original meshes with a k-nearest neighbor aggregation strategy, followed by a standard smoothing based on graph-cut for post-processing.^16^^,^^17^ The architecture of the IOSNet is illustrated in Figure 2, where we adopted the Edge-Conv block with similar architectures as in.^16^^,^^36^ More architecture details are in the Figure S2.

##### Novel loss functions in IOSNet

Though several methods have been proposed for tooth segmentation in IOS, they had limitations in precise boundary segmentation or generalization to complicated anatomies, such as crowded teeth and hyperdontia. To alleviate this issue, we proposed two novel loss functions. The first one is a centroid loss which helps learn the coarse tooth shapes, avoiding weird segmentations (recognizing two teeth as one or vice versa). The second one is boundary loss which helps produce accurate boundary predictions for complicated samples, i.e., mesh faces between tooth-tooth or tooth-gingiva boundaries.

In IOS mesh, adjacent teeth were frequently misidentified as a single tooth because of their similar structures or close connections. To achieve accurate segmentation, we designed the centroid loss to assure that the geometrical center of each tooth can be correctly captured. Specifically, given the tooth point cloud P, the centroid loss is defined as:

(Equation 7) $$L^{\text{centroid}}=\frac{1}{C}\sum_{i=1}^{C}dis\left(pc_{i}-gc_{i}\right)\text{,}$$

where C=33 denotes the number of categories in the annotations, dis(·) denotes the Euclidean distance of two points, $pc_{i}$ and $gc_{i}$ represent the prediction centroid and the gold centroid for the ith class, respectively, which are defined as:

(Equation 8) $$pc_{i}=\frac{\sum_{j=1}^{N_{i}}{\tilde{p}}_{j}\times s_{j}}{\sum_{j=1}^{N_{i}}{\tilde{p}}_{j}}\text{,}$$

(Equation 9) $$gc_{i}=\frac{\sum_{j=1}^{M_{i}}s_{j}}{M_{i}}\text{,}$$

where ${\tilde{p}}_{j}$ is defined as:

(Equation 10) $${\tilde{p}}_{j}=\left\{ \begin{array}{cc} 1\text{,} & p_{j}>th_{u}\\ 0\text{,} & p_{j}<th_{l}\\ p_{j}\text{,} & \text{otherwise} \end{array}\right.\text{,}$$

and $N_{i}$ and $M_{i}$ denote the number of points in the ith class except the gingiva in the prediction and the ground truth, $p_{j}$ denotes the probability of the jth point predicted as the ith class, and $s_{j}$ represents the 3D coordinates of the jth point. Here, we empirically defined $th_{l}$ and $th_{u}$ as 0.38 and 0.6, respectively, to eliminate points with low confidence during computing the centroids. By minimizing $L^{centroid}$, we encouraged the predictions to maintain the same geometrical centers as the ground truth, avoiding weird segmentation results.

Another challenge in 3D tooth segmentation is to accurately delineate the tooth-tooth or tooth-gingiva boundaries. To cope with the issue, we designed a novel boundary loss, which is defined as:

(Equation 11) $$L^{\text{boundary}}=L^{ce}\left(pb,gb\right)\text{,}$$

where $L^{ce}$ denotes the cross entropy loss, pb and gb represent the predicted probability and ground truth label of the boundary points, respectively. Here, the top 5% points were chosen as the boundary points according to the KL-divergence of all the points, while the KL-divergence for the ith point is defined as:

(Equation 12) $$KL_{-}div_{i}=\max_{j\in \kappa_{i}}KLD\left(c_{i},k_{j}\right)\text{,}$$

where $c_{i}$ denotes the prediction probability distribution of the center point i, and $k_{j}$ denotes the jth neighbor point’s probability distribution, KLD is the KL-divergence between two distributions. We used maximum as the aggregation operation in a local neighborhood to select the most representative value from a set of KLD values computed by probability distribution of the center point and its neighbor points. $KL_{-}div_{i}$ indicates the distributional difference between the ith point and its $K=\left|\kappa_{i}\right|$ surrounding points, while points with a large $KL_{-}div_{i}$ indicate it is of high probability to become a boundary point. We set K=5 in our implementation. Overall, the total loss for IOSNet is defined as:

(Equation 13) $$L^{\text{total}}=L^{\text{ce}}\left(p,g\right)+L^{\text{centroid}}+L^{\text{boundary}}\text{,}$$

where $L^{\text{ce}}$ denotes the cross entropy loss, p and g denote the prediction scores of all points from IOSNet and the ground truth of all points respectively.

#### Experimental setup

For TSTNet, the CBCT images were resized to random resolution within 2,048× 2,048, and subsequently randomly clipped to 512× 512 and flipped. We employed the AdamW optimizer with an initial learning rate of $6e^{-5}$ and a weight decay of 0.01, a scheduler that used linear learning rate decay, and a linear warmup of 1,500 iterations. All models were trained on NVIDIA RTX 3090 GPUs with batch size 16 for 160,000 iterations. For IOSNet, we followed the procedures in Hao et al.^16^ and Xu et al.^17^ to adopt a graph-cut-based boundary smoothing for post-processing, training all models with 10,000 points. We employed AdamW as the optimizer with an initial learning rate of $3e^{-3}$ and a weight decay of $1e^{-3}$. All models were trained on NVIDIA RTX 3090 GPUs with batch size 8 for 200 epochs.

#### Multimodal fusion of CBCT and IOS

With the segmentation outputs from the previous steps, the MF module aims to produce fused 3D tooth instances with high-fidelity toot crowns from IOS, comprehensive tooth roots from CBCT, and accurate alveolar bones from CBCT, leading to complete 3D dental models for accurate tooth crown-root-bone analysis in clinical applications.

The MF modules include three steps: 1) 3D CBCT mesh reconstruction and automatic segmentation of half jaws and individual teeth in the reconstructed mesh based on point curvature feature; 2) Registration between CBCT data and IOS tooth crown meshes (mandible and maxilla); 3) Replacement of the crowns in CBCT meshes with IOS meshes to obtain fused clinically applicable dental models, as illustrated in Figure 1 - Step 3: Multimodal Data Fusion.

##### Point curvature feature-based half-jaw segmentation

**Figure 5:**
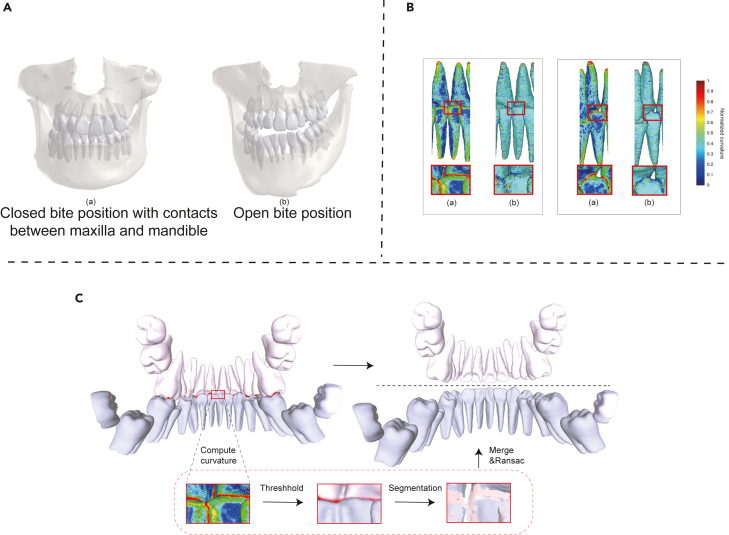
The details of the point curvature-based half-jaw segment (A) The reconstructed 3D CBCT meshes. (Aa) Closed bite position with contacts between maxilla and mandible. (Ab) Open bite position. (B) The visualization of colorized curvatures. (Ba) Our point curvature. (Bb) Gaussian curvature. (C) The half-jaw segmentation processing based on point curvature.

The MF module first reconstructs the 3D meshes for the teeth and alveolar bones from CBCT based on the standard marching-cube reconstruction and HLO smoothing algorithms.^73^ The CBCT and IOS usually have different bite positions. The CBCT usually includes both upper and lower jaws with close or open bite positions as in Figure 5A, while the IOS usually includes separated half jaws. Hence, to accurately fuse both modalities, a promising method is to first get the upper and lower jaws in CBCT, and then perform half-jaw registration and fusion between CBCT and IOS. However, as shown in Figure 5A, there usually exist connected boundaries between adjacent teeth or the contacts in maxilla and mandible, especially for patients in close bite positions, which imposes significant difficulties for accurate half jaw registration and individual teeth delineation. To address this issue, we proposed a geometry-based segmentation algorithm to separate the half jaws and further delineate each tooth in CBCT meshes with a novel point curvature feature.

Specifically, the point curvature feature of a vertex is defined as the average of angles between normal vectors of all its neighbors, which is different from either mean curvature or Gaussian curvature. Mathematically, given an vertex v with normal vector $n_{v}$, we define its first-order neighbors as u∈N(v), associated with normal vectors $n_{u}$, the point curvature feature $cur_{v}$ of vertex v is defined as:

(Equation 14) $$cur_{v}=\frac{1}{\left|N\left(v\right)\right|}\sum_{u\in N\left(v\right)}\arccos\left(\frac{n_{v}\cdot n_{u}}{\left|n_{v}\right|\left|n_{u}\right|}\right)\text{.}$$

This definition can be extended to lth-order neighbors by changing the set of neighbors. The intuition behind the curvature-based segmentation algorithm is that the angle between the normal vectors of adjacent points can capture the curvature of different scales by changing the level of neighbors. We experimentally found that this curvature could help recognize the ambiguous boundaries between adjacent teeth, and the upper and lower jaw contacts, while the traditional curvatures such as Gaussian curvature usually failed in such scenarios, as shown in Figure 5B and demonstrated in the following experiments.

We applied a point curvature feature-based region growth segmentation algorithm, an “erosion-expansion” procedure, to separate the upper and lower jaws, as shown in Figure 5C. The vertices with high curvature values can serve as boundaries even with connected teeth in maxilla and mandible. We first computed the point curvature feature for each vertex, and removed vertices with curvatures in the top T percent from the mesh (erosion). Followed by a simple connected component analysis algorithm, we separated the individual teeth, and merged back the deleted vertices to the nearest separated components, on top of which the KD-trees are constructed, to get unbroken reconstructed teeth (expansion). Based on individual teeth, we generated the half jaws by computing the gravity center of each tooth and separating them into upper and lower parts with a RANSAC algorithm.^74^ Additional technical algorithm details were attached in the Data S1, S2 and S3.^75^

##### Registration of two modalities

The CBCT segmentation results from the previous section are maxillary and mandibular parts, which are to be registered with the IOS meshes. We denote the vertex set of the IOS mesh as $V_{I}$, and the vertex set of the CBCT mesh as $V_{C}$. The registration method is to find the optimal transformation $T^{*}=\left[R|t\right]$ such that the transformed point cloud $T^{*}\left(x\right)$ best aligns with the target, in which R∈SO(3) (3D rotation group in geometry) denotes the rotation, $t\in R^{3}$ denotes the translation. The registration formula is defined as follows:

(Equation 15) $$T^{*}=\underset{T}{\mathrm{argmin}}\sum_{\left(v_{i},v_{c}\right)\in Corr}{\left\|v_{c}-T\left(v_{i}\right)\right\|}^{2}\text{,}$$

where T is the set of rigid transformations represented by a 4× 4 matrix. $T^{*}$ is the optimal transformation. Corr is a set of correspondences between $V_{I}$ and $V_{C}$, where $v_{c}\in V_{C},v_{i}\in V_{I}$.

We proposed a two-stage registration method composed of a global registration to roughly register the two point sets as the initial alignment, and an improved ICP registration step for further refinement. In the global registration, the CBCT and IOS meshes are first down-sampled with voxel size of 0.6 and then registered with a RANSAC scheme on the 33-dimensional FPFH vector space.^76^ The segmentation is further improved by the multiscale ICP algorithm performing point-to-plane ICP registration at three scales. In particular, we performed multiscale ICP with point-to-plane ICP registration^77^ at three sequential scales (with distance thresholds of 0.36, 0.18, 0.12)^78^:

(Equation 16) $$T^{i}=\underset{T}{\mathrm{argmin}}\sum_{\left(v_{i},v_{c}\right)\in Corr^{i}}{\left\|v_{c}-T^{i-1}\left(v_{i}\right)\right\|}^{2},i=1,2,3\text{,}$$

where $T^{0}$ is the global rigid transformation obtained at the distance threshold of 0.36, and $T^{1},T^{2}$ denote the corresponding iterative multiscale registration with distance thresholds 0.18 and 0.12, respectively. Finally, we took the transformed mesh by $T^{2}$ as the registration output.

##### Tooth crown replacement with mesh fusion

The final step in this MF module is to replace the low-resolution and error-prone CBCT crown with the high-fidelity and clinically applicable IOS crown. We performed the fusion on the point level, taking the registration results as the inputs. Specifically, the points $V_{I}$ in the IOS mesh $M_{I}$ were taken as reference, and the points belonging to the CBCT crown, i.e., $V_{C}$ in $M_{C}$, were removed. These points were identified based on the Euclidean distance of the points in $V_{C}$ to the KDTree constructed from IOS point clouds $V_{I}$. With an empirical threshold value on the Euclidean distance, followed by a simple DBSCAN algorithm^40^ to detect the isolated outlier points, we were able to easily identify, crop, and replace the tooth crown points in $V_{C}$ with points in $V_{I}$. Finally, we got the fused mesh P based on the fused point cloud and the original normal vectors from the IOS and CBCT mesh $\left(M_{I},M_{C}\right)$ with Poisson reconstruction. More technical details regarding the tooth crown replacement steps are attached in the Data S4.^75^

## Data Availability

• The clinical CBCT and IOS data were collected by the hospitals in de-identified format. Owing to patient-privacy constraints, we are not able to release all dataset publicly. A partial release of the dataset is available at Zenodo under the https://doi.org/10.5281/zenodo.8027553^67^ and is publicly available as of the date of publication. • All original code has been deposited at Zenodo under the https://doi.org/10.5281/zenodo.8027716^68^ and is publicly available as of the date of publication. • Any additional information required to reanalyze the data reported in this paper is available from the lead contact upon request.

## Supplemental information

Document S1. Tables S1–S6, Figures S1–S7, and supplemental experimental procedures

Data S1. Curvature-based segmentation

Data S2. Merge process

Data S3. Curvature-based segmentation on 3D CBCT mesh

Data S4. Tooth crown replacement

Document S2. Article plus supplemental information
