# Supplementary material for: Deep learning-enabled 3D multimodal fusion of cone-beam CT and intraoral mesh scans for clinically applicable tooth-bone reconstruction
Source: Patterns (N Y). 2023 Aug 15;4(9):100825. doi: 10.1016/j.patter.2023.100825 (PMC10499902; doi:10.1016/j.patter.2023.100825)
Supplement: Document S1. Tables S1–S6, Figures S1–S7, and supplemental experimental procedures [file mmc1.pdf]

Patterns, Volume *4*

Supplemental information

Deep learning-enabled 3D multimodal fusion of cone-beam CT and intraoral mesh scans for clinically applicable tooth-bone reconstruction

Jiaxiang Liu, Jin Hao, Hangzheng Lin, Wei Pan, Jianfei Yang, Yang Feng, Gaoang Wang, Jin Li, Zuolin Jin, Zhihe Zhao, and Zuozhu Liu

Patterns, Volume *4*

Supplemental information

Deep learning-enabled 3D multimodal fusion of cone-beam CT and intraoral mesh scans for clinically applicable tooth-bone reconstruction

Jiaxiang Liu, Jin Hao, Hangzheng Lin, Wei Pan, Jianfei Yang, Yang Feng, Gaoang Wang, Jin Li, Zuolin Jin, Zhihe Zhao, and Zuozhu Liu

Layers	Output size (Output name)	TSTNet
Input	h x w x 3	
Patch Embedding	h/4 x w/4 x 128	[Patch Partition → h/4 x w/4 x 48<br>Linear Embedding: 4x4, dim 128 Conv2d, stride 4 → LN]
Swin Block	h/4 x w/4 x 128	[LN → window size 7, dim 128, head 4 W-MSA → Residual connect<br>LN → MLP → Residual connect<br>LN → window size 7, dim 128, head 4 SW-MSA → Residual connect<br>LN → MLP → Residual connect]
Swin_stage2	h/8 x w/8 x 256	[Patch Merging [group feature map → h/8 x w/8 x 4 x 128<br>LN → dim 128 x 2 linear → h/8 x w/8 x 256]<br>window size 7, dim 256, head 8 Swin Block]
Swin_stage3	h/16 x w/16 x 512	[Patch Merging [group feature map → h/16 x w/16 x 4 x 256<br>LN → dim 256 x 2 linear → h/16 x w/16 x 512]<br>window size 7, dim 512, head 16 Swin Block] x 9
Swin_stage4	h/32 x w/32 x 1024<br>(pred seg)	[Patch Merging [group feature map → h/32 x w/32 x 4 x 512<br>LN → dim 512 x 2 linear → h/32 x w/32 x 1024]<br>window size 7, dim 1024, head 32 Swin Block]
TEC<br>[discard when infering]	(metric loss)	[Compute TP, FN, FP(GT labels, pred seg)<br>Compute category prototype(TP pixel) by EMA<br>Compute cos similarity(pixel, category prototype)<br>Error calibration [compute FP penalty term(FP pixel, anchor)<br>compute FN penalty term(FN pixel, anchor)]<br>Compute metric loss(cos similaity, penalty, compensation)]
FCN Head<br>[auxiliary head, discard when infering]	2 x h x w	[3 x 3, dim 256 Conv2d → SyncBN<br>3 x 3, dim 256 Conv2d → SyncBN<br>ratio 0.1 Dropout2d → 1x1, dim 2 Conv2d]
UperNet Head<br>[decode head]	2 x h x w	[head_input channels [128, 256, 512, 1024]<br>[channels 128 → 1 x 1, dim 512 Conv2d → SyncBN → out_128<br>channels 256 → 1 x 1, dim 512 Conv2d → SyncBN → out_256<br>channels 512 → 1 x 1, dim 512 Conv2d → SyncBN → out_512]<br>channels 1024 → [scale 1 AdaptiveAvgPool → 1 x 1, dim 512 Conv2d → SyncBN<br>scale 2 AdaptiveAvgPool → 1 x 1, dim 512 Conv2d → SyncBN<br>scale 3 AdaptiveAvgPool → 1 x 1, dim 512 Conv2d → SyncBN<br>scale 6 AdaptiveAvgPool → 1 x 1, dim 512 Conv2d → SyncBN]<br>→ concat along channel dim → 3 x 3, dim 512 Conv2d → SyncBN → out_1024<br>[out_128 → 3 x 3, dim 512 Conv2d → SyncBN<br>out_256 → 3 x 3, dim 512 Conv2d → SyncBN<br>out_512 → 3 x 3, dim 512 Conv2d → SyncBN]<br>SyncBN ← 3 x 3, dim 512 Conv2d ← concat along channel dim ←<br>→ ratio 0.1 Dropout2d → 1x1, dim 2 Conv2d]

Figure S1: **The details of TSTNet.**

Layers	Output Size (output name)	IOSNet
Input	b x n x 15	
Get_graph_feature	b x 30 x n x k	knn(k) → b x n x k<br>get_neighbor_feature → b x n x k x 15<br>offset_feature = neighbor_feature — center_point_feature → b x n x k x 15<br>concat offset_feature and center_point_feature along channel dimension → b x n x k x 30
Transformation Net	b x n x 15	1x1, 128 Conv2d → BatchNorm → Mish activation<br>1x1, 256 Conv2d → BatchNorm → Mish activation<br>max pool along k_neighbor dim → b x 256 x n<br>1x1, 1024 Conv1d → BatchNorm → Mish activation<br>max pool along n dim → b x 1024<br>512-d linear → Mish activation<br>256-d linear → Mish activation<br>225-d linear → reshape as b x15 x15(transform matrix)<br>input * transform matrix
EdgeConv_1	b x 64 x n<br>(edgeconv1)	get_graph_feature<br>1x1, 128 Conv2d → BatchNorm → Mish activation<br>1x1, 128 Conv2d → BatchNorm → Mish activation<br>1x1, 64 Conv2d → BatchNorm → Mish activation<br>max pool along k_neighbor dim
EdgeConv_2	b x 64 x n<br>(edgeconv2)	get_graph_feature<br>1x1, 64 Conv2d → BatchNorm → Mish activation<br>1x1, 64 Conv2d → BatchNorm → Mish activation<br>1x1, 64 Conv2d → BatchNorm → Mish activation<br>max pool along k_neighbor dim
EdgeConv_3	b x 64 x n<br>(edgeconv3)	get_graph_feature<br>1x1, 64 Conv2d → BatchNorm → Mish activation<br>1x1, 64 Conv2d → BatchNorm → Mish activation<br>max pool along k_neighbor dim
Get_global_feature	b x 1024<br>(global_feature)	concat (edgeconv1, edgeconv2, edgeconv3)<br>1x1, 1024 Conv1d → BatchNorm → Mish activation<br>max pool along n dim
Category Vector	b x 2	
Add_category_vector	b x 1088 x n<br>(new_global_fea)	1x1, 64 Conv1d → Mish activation → category_feature<br>concat(global_feature, category_feature)
Get_all_feature	b x 1280 x n	concat (new_global_fea, edgeconv1, edgeconv2, edgeconv3)
Segmentation head	b x 33 x n	1x1, 256 Conv1d → BatchNorm → Mish activation<br>dropout<br>1x1, 256 Conv1d → BatchNorm → Mish activation<br>dropout<br>1x1, 128 Conv1d → BatchNorm → Mish activation<br>1x1, 33 Conv1d

Figure S2: **The details of IOSNet.**

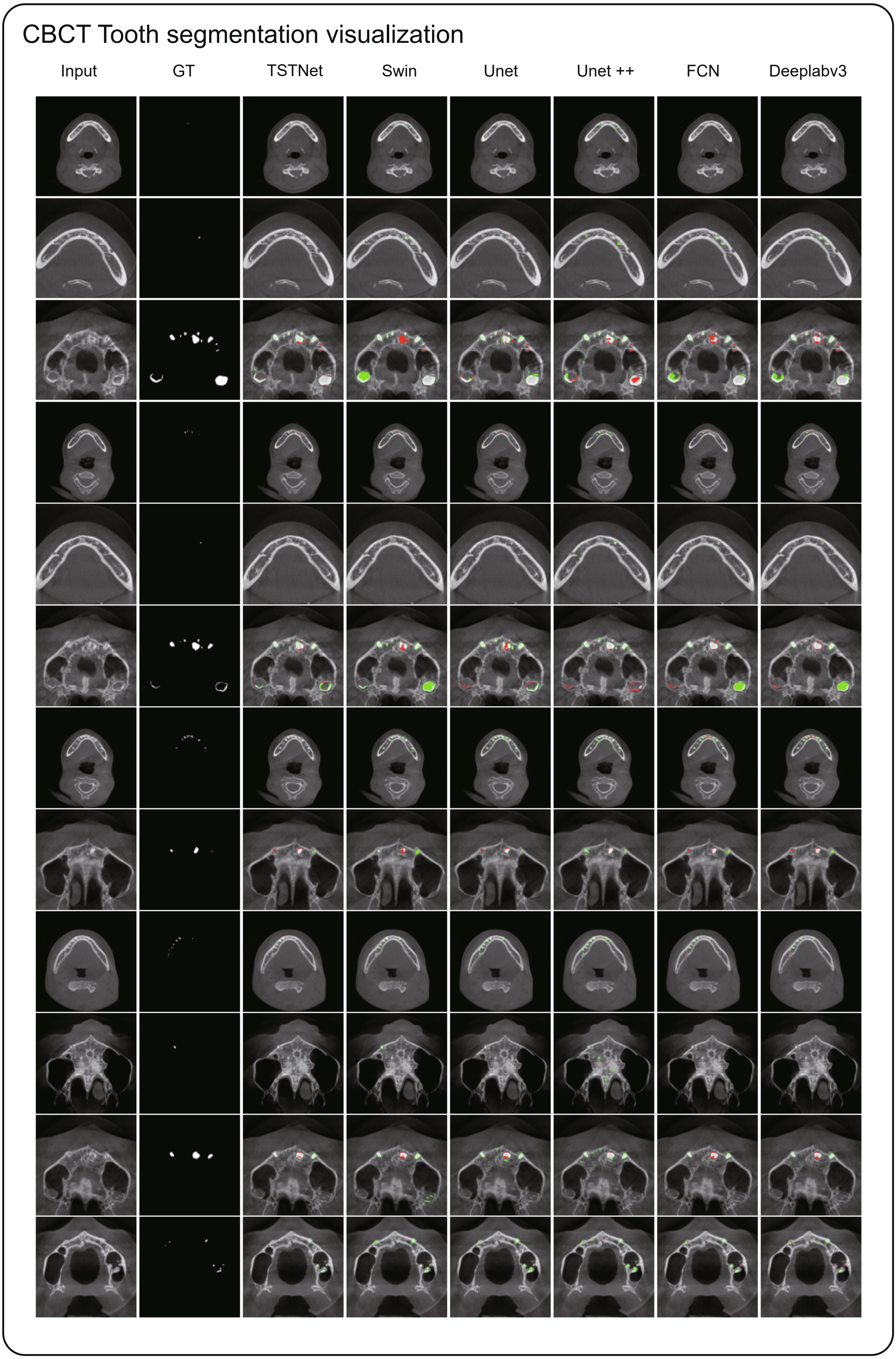

Figure S3: **More visualizations of tooth segmentation results.** TSTNet can accurately segment the pixels and greatly calibrate the FP and FN pixels. The results of TSTNet are the closest to the ground truth.

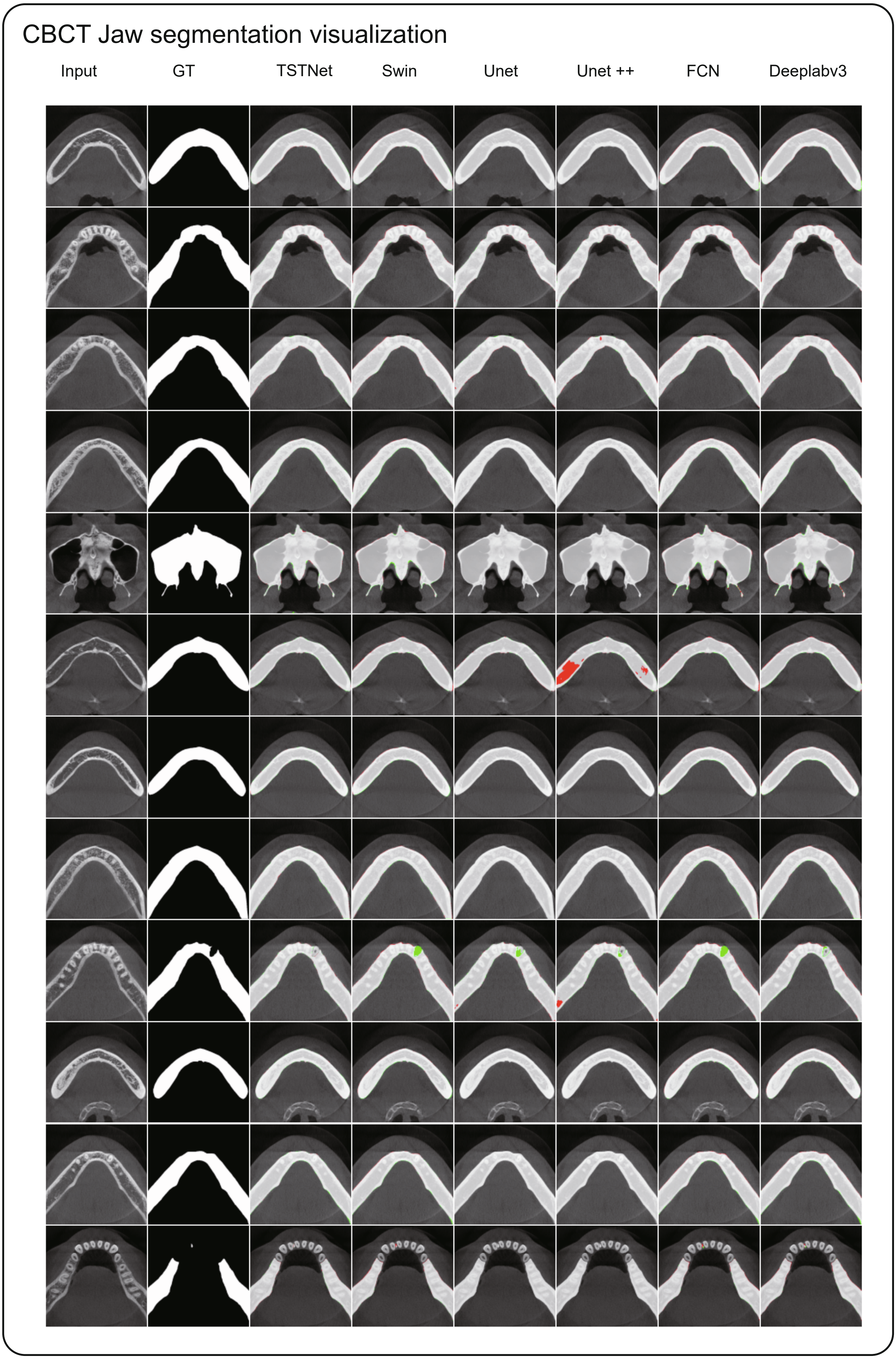

Figure S4: **More visualizations of jaw segmentation results.**

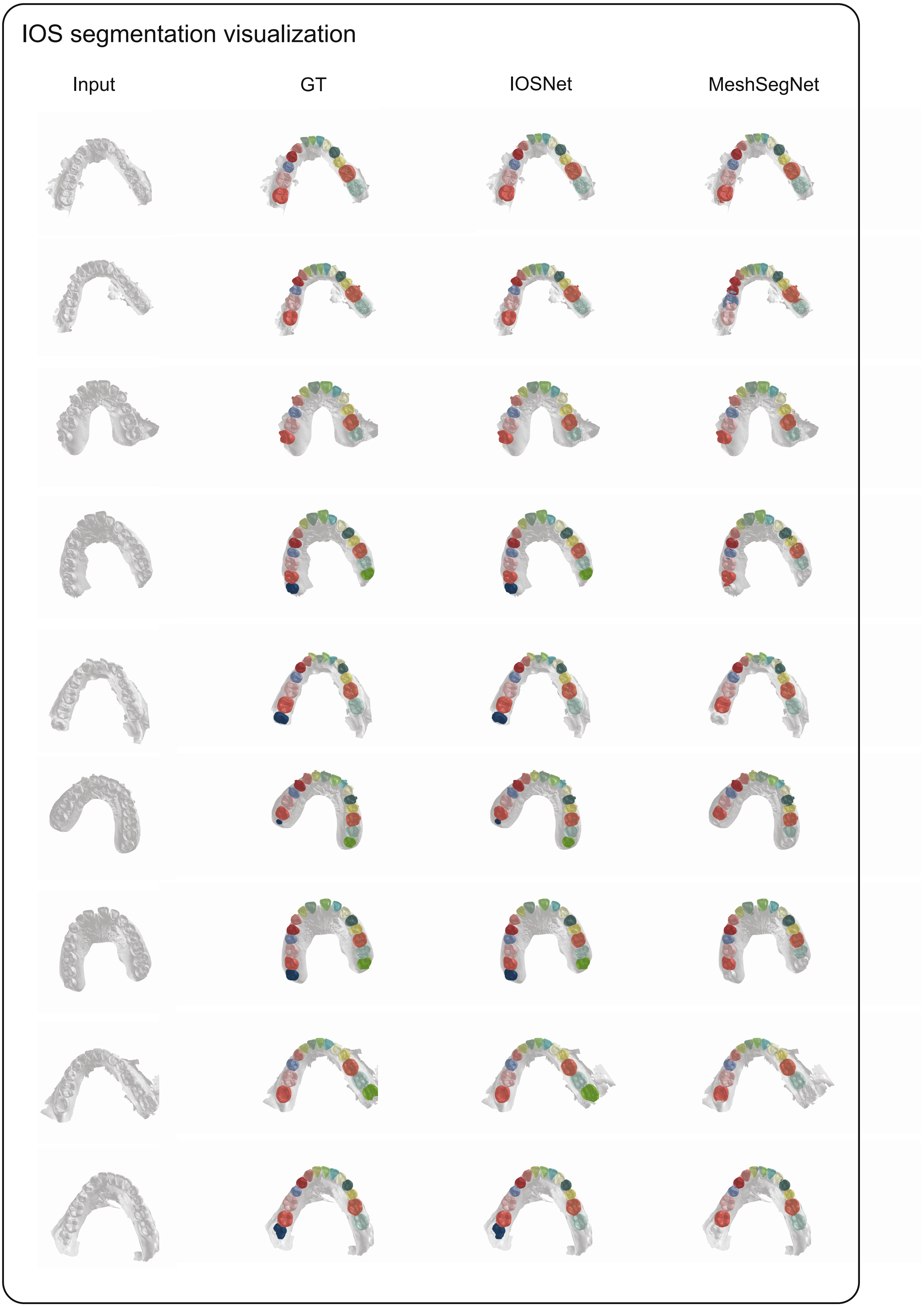

Figure S5: **More visualizations of IOSNet results.** IOSNet can get smoother and more accurate tooth segmentation results than MeshSegNet. In terms of the abnormal tooth numbers (the 1st row), our IOSNet can identify the incisors accurately.

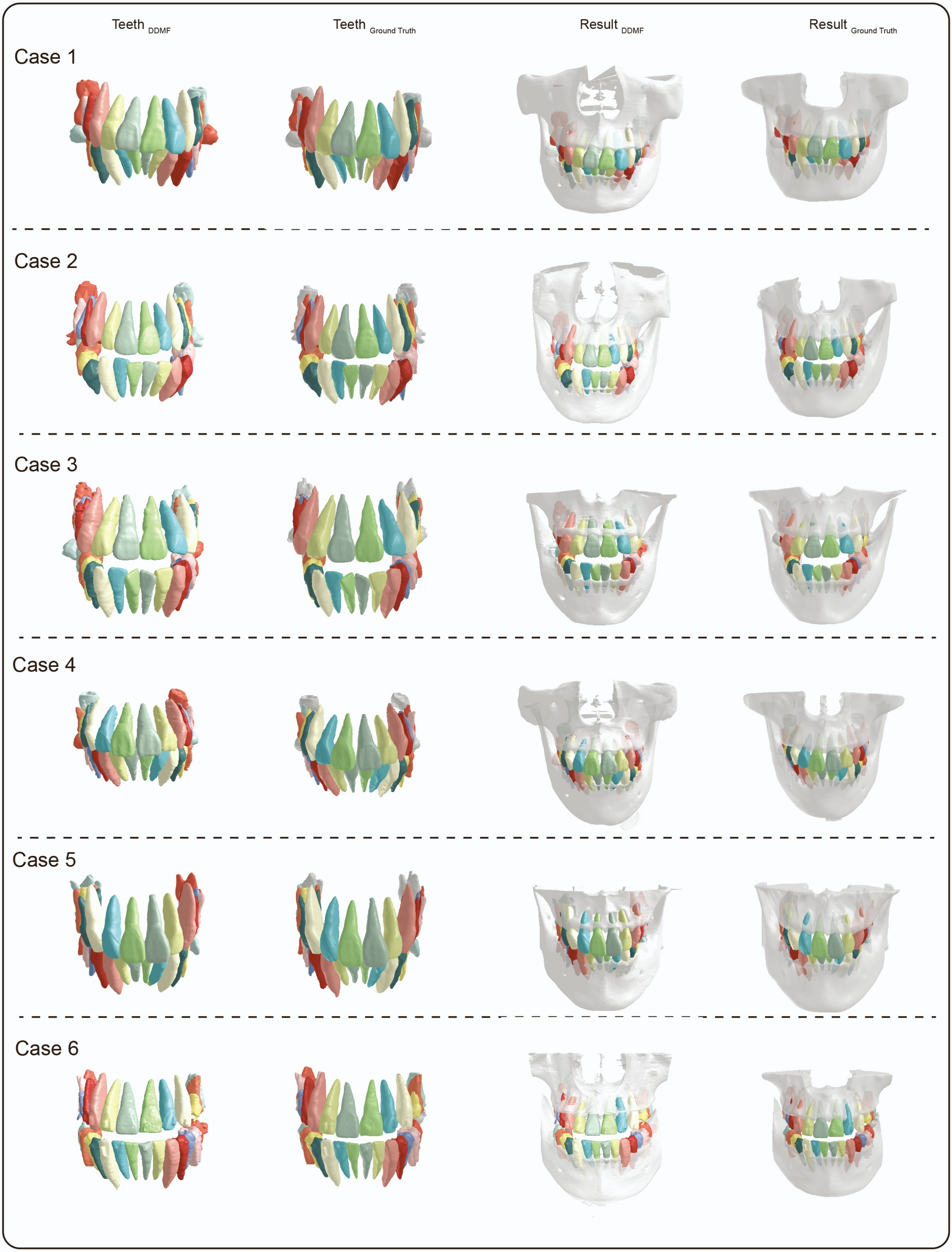

Figure S6: **More visualizations of DDMF results.** The quality of the DDMF result is approximate to the ground truth labeled by professional experts.

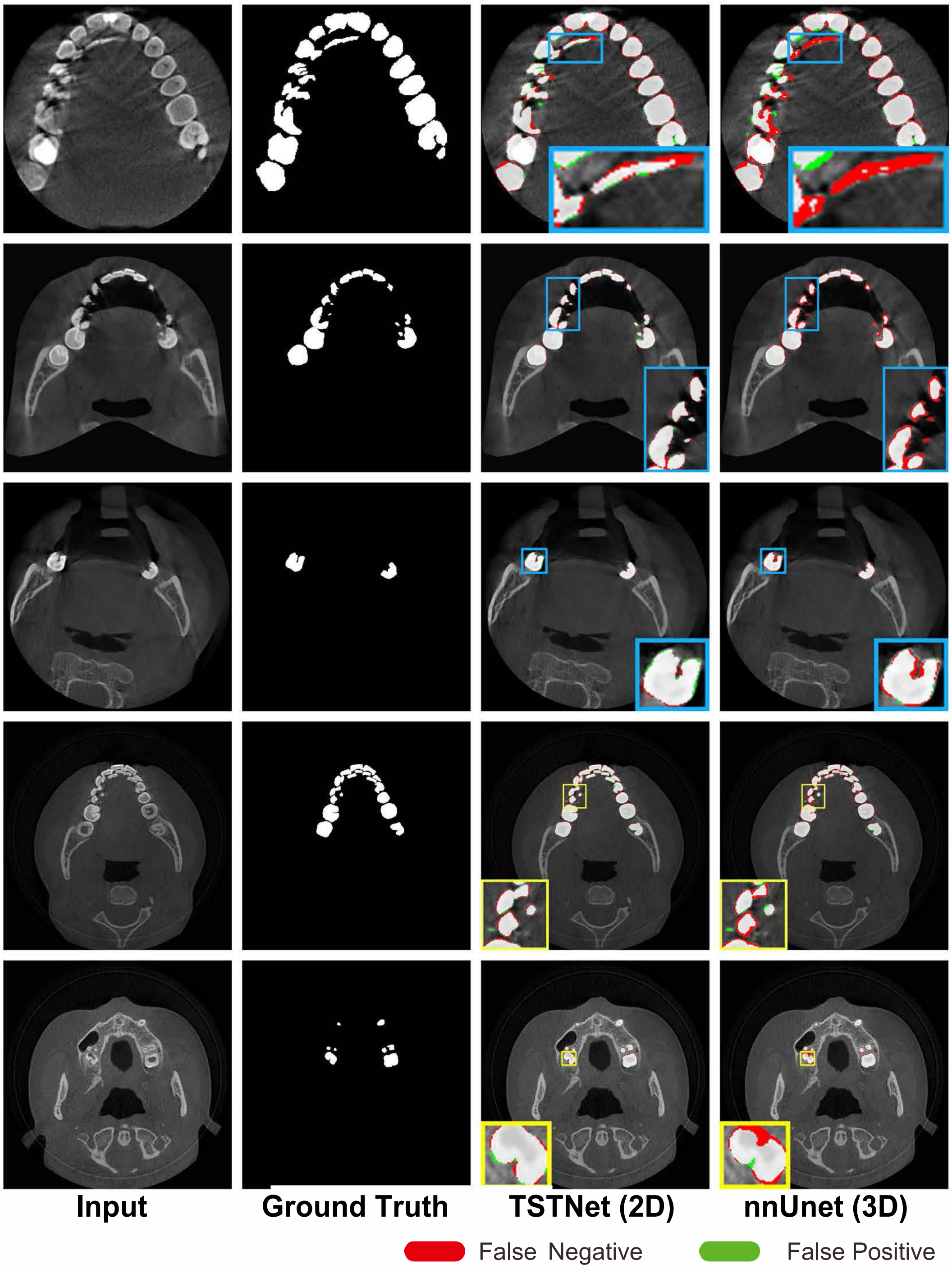

Figure S7: **Visualization of segmentation results for TSTNet and nnUnet.** We annotated 30 cases in 3D using 3D Slicer, and then trained nnUnet, a powerful backbone for many 3D segmentation tasks, with these 30 annotated cases. Visualizations demonstrate the superior performance of TSTNet, which commits fewer false positive and false negative errors than nnUnet.

Table S1: **Model complexity and clinical utility of CBCT segmentation (Tested on n=43 patients).** The number of parameters in TSTNet is approximately the same as the standard Swin transformer.

**Models**	**Unet**	**Unet++**	**FCN**	**Deeplabv3**	**Swin**	**TSTNst (w/o aug)**	**TSTNet**	**TSTNst (multi-scale)**	**Human Experts**
#Params(M)	29.62	34.95	49.48	68.10	121.17	121.43	121.43	121.43	-
Inf-T(s)	~0.072	~0.085	~0.076	~0.066	~0.071	~0.052	~0.479	~3.070	~600-900

#Params: number of parameters in the neural networks. Inf-T: end-to-end inference time of models. ~ means approximately. “w/o aug” denotes TSTNet with standard augmentation, i.e., without cropping and domain specific augmentation.
“multi-scale” denotes TSTNet with multi-scale inference.

Table S2: **Model Complexity and Clinical Utility of IOS Segmentation (Tested on n=100 patients).** It indicates that the number of parameters and the inference time of IOSNet are almost the same as the DC-Net. Compared with the baseline, although the IOSNet has more parameters, its inference speed is one order of magnitude faster than the baseline, and at least 25 times faster than the human experts, which shows that the IOSNet can achieve highly accurate segmentation at a faster speed. Therefore, our IOSNet has a promising future in clinical dentistry.

**Models**	**Baseline**	**DC-Net**	**IOSNet**	**Human Experts**
#Params(M)	0.4	1.5	1.5	-
Inf-T(s)	~321	~24	~24	~600-900

#Params: number of parameters in the neural networks. Inf-T: end-to-end inference time of models.
~ means approximately.

Table S3: **Evaluation of the registration between reconstructed CBCT meshes and IOSscans (Tested on 30 groups data).** Our registration method can achieve the best performance in the Registration of two modal data.

Method	Super4pc	AAICP	Ours
Fitness	0.1120	0.0573	**0.3100**
Inlier_rmse	0.0858	0.0866	**0.0823**
Correspondence_set size	7933.9	4009.5	**21270.9**
Success rate	13.30%	6.67%	**90%**

Fitness, which measures the overlapping area. The higher the better.

Inlier_rmse, which measures the RMSE of all inlier correspondences. The lower the better.

Table S4: **Tooth multimodal fusion Metrics of the proposed method (Tested on 479 teeth in 20 cases).**

Case	ASSD (mm)	CD (mm)	HD (mm)
Case01	0.14±0.04	0.17±0.02	0.35±0.22
Case02	0.18±0.02	0.21±0.02	0.40±0.05
Case03	0.18±0.03	0.21±0.03	0.39±0.08
Case04	0.14±0.02	0.17±0.02	0.31±0.06
Case05	0.25±0.07	0.27±0.07	0.51±0.10
Case06	0.11±0.02	0.14±0.02	0.21±0.04
Case07	0.17±0.04	0.19±0.05	0.35±0.13
Case08	0.14±0.02	0.17±0.02	0.30±0.09
Case09	0.14±0.03	0.17±0.02	0.32±0.07
Case10	0.10±0.02	0.14±0.02	0.23±0.06
Case11	0.12±0.06	0.15±0.06	0.28±0.16
Case12	0.11±0.05	0.14±0.04	0.26±0.12
Case13	0.10±0.02	0.14±0.02	0.23±0.05
Case14	0.13±0.04	0.16±0.03	0.31±0.08
Case15	0.21±0.09	0.23±0.07	0.48±0.23
Case16	0.15±0.08	0.18±0.06	0.34±0.17
Case17	0.12±0.03	0.15±0.03	0.26±0.07
Case18	0.15±0.02	0.17±0.02	0.30±0.06
Case19	0.22±0.03	0.24±0.03	0.57±0.13
Case20	0.21±0.09	0.24±0.07	0.59±0.55

ASSD: Average symmetric surface distance; HD: Housdoff distance; CD: Chamfer distance.

Table S5: **Segmentation results of TSTNet and nnUnet (Tested on 15 patients, which has 295 slices).** We annotated 30 cases in 3D using 3D Slicer, and then trained nnUnet, a powerful backbone for many 3D segmentation tasks, with these 30 annotated cases. TSTNet exhibits better slice-wise segmentation results than nnUnet.

**Model**	**IoU (%)**	**Dice (%)**	**Inf-T (s)**
nnUNet	85.02	91.90	~600
TSTNet	**88.77**	**94.05**	**~140**

IoU: intersection over union; Dice: Dice Coefficient. Bolden numbers indicate the best performance.

Inf-T: end-to-end inference time of models for a single patient.

Table S6: **CBCT information**

#CBCT scans	503 (more than 150,000 slices)
#Annotated slices	9,651
Age	19.53 ± 7.57 (mean ± std)
Male/Female	32.5%/67.5%
Pixel resolution of 2D CBCT slices	[260, 260] to [1000, 1000]
**Imaging protocols**	
Voxel resolution	[0.3, 0.3, 0.3], [0.266, 0.266, 0.251], [0.2, 0.2, 0.2], [0.25, 0.25, 0.25], [0.226, 0.226, 0.226], [0.18, 0.18, 0.18], [0.35, 0.35, 0.35], [0.16, 0.16, 0.16], [0.246, 0.246, 0.245], [0.183, 0.183, 0.183], [0.227, 0.227, 0.227], [0.319, 0.319, 0.322], [0.5, 0.5, 0.5], [0.28, 0.28, 0.28], [0.337, 0.337, 0.3], [0.22, 0.22, 0.22], [0.15, 0.15, 0.15], [0.256, 0.256, 0.256], [0.32, 0.32, 0.269], [0.125, 0.125, 0.5]
CBCT machine	HYBPLUS, SS-X9010DPro-3DE, PHT-35LHS, HighRes3D, PAPAYA 3D, CS 9300 Select, NTVGiEVO, XG3D, SS-X10010DPlus, Point 800 3D Plus 8N, Alioth, SS-X9010DPro, K9500, RAYSCAN Alpha, skyView, ORTHOPHOS SL, CS 8100SC 3D, Point 3D Combi 500 Series, SS-X9010DMax

Supplemental Experimental Procedures

Dataset: The dataset includes 503 samples with both CBCT and IOS collected from hospitals and clinics in 25 provinces in China during 2018-2021. The 503 samples are also associated with ground truth 3D reconstructions with fused IOS tooth crowns and CBCT tooth bones, all annotated by a committee of human experts. The 503 patients are at age 19.53±7.57 years old, with 32.5% male and 67.5% female. Besides, we collected an extra 28,559 IOS meshes. The tooth segmentation network is trained with 5-fold cross validation each with 50 patients. The jaw segmentation network is trained with 840 slices and tested on 246 slices. The IOS segmentation network is evaluated with 200 IOS scans as in the baselines.

Data annotation for 3D CBCT scans is more time-consuming and expensive compared to annotating selected 2D slices, as indicated by existing literature [1,2] and our experience. It typically takes 150-240 minutes to get a coarse label for one 3D CBCT scan using interactive tools like 3DSlicer, and an additional 60-120 minutes to have experts double-check annotations. On the other hand, annotating one 2D CBCT scan with 15-20 slices takes 30-40 minutes, and experts can double-check annotations in 10 minutes with an observational error of 1-2 pixels. Due to this difference in annotation time, most 3D-based CBCT studies use small datasets or datasets with coarse annotations. Using 2D segmentation methods allows for leveraging large and high-quality datasets. Additionally, general 3D segmentation methods require more computational resources and exhibit larger inference latency[3]. For instance, segmenting one 3D CBCT scan using a standard 3D nnUnet [19] takes over 10 minutes with NVIDIA GTX 3090 GPUs. This long response time and GPU demand necessitate optimization of both algorithm design and engineering implementation to meet real-world clinical applications. Therefore, we use 2D segmentation instead of 3D to deal with CBCT segmentation.

We annotated 9,651 CBCT slices of the 503 patients for CBCT tooth segmentation, where we select 15-25 slices for each patient. Similarly, we annotated 1,066 images for CBCT jaw segmentation. The slices are selected to maximize the difference among slices, e.g., for a CBCT with 400 slices, we might start annotation at slice 100 where tooth apex are first scanned, and subsequently select in total 15-25 slices to annotate every 10 slices, avoiding annotating highly-correlated and highly-similar slices. By doing so we can get annotated slices that contain different anatomical information, which could help improve the robustness and generalization ability of our model. We choose the slices for the jaw annotation following the same procedure.

The annotation process for CBCT slices is three-stage. First, each slice is annotated by the senior undergraduates or master students majoring in stomatology. Second, the annotated slices are revised and corrected by a committee of experts who hold doctoral degrees in stomatology and at least 5 years clinical experience. Finally, several senior radiologists with more than 10 years experience are going to work with the expert to further refine the labels, and their consensus is regarded as the ground truth. The images are annotated with the Labelme software, while a small portion of them are also examined by the Mimic software. The details of the CBCT information can be found in Table S6.

The annotation process for the IOS dataset is as follows. Given an input mesh scan, the experts can use the software “Atreat Processor” to annotate each mesh face. For each patient, a junior technician with 1-5 years working experience in dentistry will first annotate the IOS scan, with the help of automic solutions such as DC-Net. Afterwards, the annotation will be examined by a senior technician with more than 5 years working experience and a dentist (usually an orthodontist).

The annotations of the fused tooth-bone meshes which are going to be used to compute the 3D fusion performance such as ASSD are also conducted by a committee of human experts assisted by CAD software. During annotation, the experts need to decide whether the annotation is satisfactory for future oral diagnosis and treatment planning. If all of them (usually three experts) agree that the annotation is satisfactory, we will take that annotation. Otherwise, the annotation needs to be improved until it is good enough for future clinical usage.

Data preprocessing: In training, this paper proposed a data augmentation strategy for CBCT data. Firstly, based on the prior and empirical statistics from the annotated masks, we cropped the lower 1/4 and right/top/left 1/10 in the original CBCT image to partially alleviate the class imbalance problem. The CBCT images are resized to random resolution within [2048, 2048] with a ratio range of is (0.5, 2.0), and randomly clipped to [512, 512]. Then, the random horizontal flipping probability of 0.5, and random photometric distortion are performed.

As for the IOS scans, we first uniformly sample 10,000 faces from the original mesh and regard each face center as a point to form a point cloud with 10,000 points. The features associated with each

point include three parts: 1. the position of the face, i.e., the 3D coordinate of the face center denoted as $h_c = (x_c, y_c, z_c) \in R^3$; 2. the 3-dimensional normal vector of the face surface $h_n \in R^3$; 3. the face shape feature $f_s \in R^9$. For each face with three vertices as $(x_i, y_i, z_i)$for i from 1 to 3, and a face center (x_c, y_c, z_c), the face shape feature is defined as $Concat(x_i - x_c, y_i - y_c, z_i - z_c)$ for i from 1 to 3. $Concat(\cdot)$ is the concatenate operation for vectors, leading to a 9-dimensional shape feature for each face. In consequence, the final output after data preprocessing for each IOS mesh scan is a point cloud with 10,000 points, each associated with a 15-dimensional feature vector $h = Concat(h_c, h_n, h_s) \in R^{15}$. This preprocessing procedure can be finished with a modern computer on-the-fly.

We apply data augmentation with four different perturbations to enlarge the IOS dataset. We independently sample 10,000 points every time for each perturbation. The perturbation is either random rotation by -10 to 10 degrees or random translation by -10 to 10 millimeters, along with one of the x, y, and z axes. The network is trained for 100 epochs. The model is evaluated on a validation set with 100 scans, and the model with the highest validation accuracy is selected for testing. During testing, we sample 40,000 points for each mesh, which will be segmented. For the rest points (i.e., faces), we choose five coordinate-based nearest neighbors from the 40,000 points, and the neighbor with the highest probability will determine the label for that point.

For CBCT jaw data, 840 CBCT images were for training, and 246 images were tested. In training, random horizontal flip and random scale are applied to augment the input image, random crop and padding are implemented to set the image size [512, 512].

TSTNet Details: The architecture details of TSTNet are shown in Figure S1. The OHEM and Lovasz loss are implemented as the reference [4], which assigns the weights (1:2) to background and tooth classes. The minimum keep pixels parameter in OHEM loss is 0.7.

IOSNet Details: The architecture details of IOSNet are shown in Figure S2.

Mesh reconstruction: The CBCT mesh is reconstructed from the CBCT segmentation results. The marching cubes algorithm is used to process CBCT image data to obtain the complete reconstructed mesh of all the CBCT teeth mesh [1]. Afterwards, the HLO is applied to smooth the surfaces to reduce the sharp creases on the surface caused by image pixels and marching cube algorithms [2]. Then, we got a satisfactory CBCT mesh.

Point Curvature Feature based Segmentation: In our method, the point curvature feature of a vertex is defined as the average of angles between normal vectors of all its neighbors, which is different from either mean curvature or Gaussian curvature, as defined in the Method. The point curvature feature is used to separate the maxilla and mandible, as well as delineate each tooth in the reconstructed 3D CBCT mesh.

We propose a new segmentation algorithm to separate the half jaws and each tooth using this novel point curvature feature. The intuition behind the curvature-based segmentation algorithm is that the angle between the normal vectors of adjacent points can capture the curvature of different scales by changing the level of neighbors. The algorithm is more or less like an “erosion-expansion” procedure. We first compute the point curvature feature for each vertex, and vertices with curvature in the top T percent would be removed from the mesh (erosion). Followed by a simple connected component analysis algorithm, the individual teeth can be separated. We will use 2nd order neighbors and $T = 20\%$ so that the main bodies of the teeth can be roughly separated. The general curvature-based segmentation algorithm is detailed in Algorithm 1 (Data S1). After the segmentation is applied, we will get both the unrefined teeth and the removed segments. After that, we extract every connected component from the remaining mesh as a single tooth. Small segments (experimentally, less than 2000 vertices) are discarded as noises. Then, we build the k-d tree for each estimated tooth, and we merge the removed point to its closest tooth. The details of the merge process are shown in Algorithm 2 (Data S2).

To better leverage the precision and efficiency of the k-d tree, we propose a multi-step approach during the merge process. We use T_{iter} steps for the merge process. In each step, we will merge the $1/T_{iter}$ removed points back to the teeth. Then we rebuild all the k-d trees based on the updated tooth points, and so on. The larger T_{iter}, the better merge performance. In the extreme case, we update the k-d tree after merging one point. Typically, we choose $T_{iter} = 5$ to balance the trade off between merging efficiency and performance. We take the minimum and the maximum z-axis coordinates of points as the reference, and classify each tooth to the maxilla or mandible by comparing the distance from the tooth centroid to the two references. As a result, the maxilla and mandible are separated and classified for registration in the next step. The maxilla and mandible segmentation algorithm is detailed in Algorithm 3 (Data S3).

Tooth crown replacement algorithm: When the registration IOS data is obtained, the next step is to fuse the CBCT with IOS scans to complete the integration of 3D crown and root data. The goal of this step is to replace the points and geometry corresponding to the tooth crown in CBCT with the IOS scan. We propose a tooth crown replacement algorithm, which consists of two steps: (1) Half-jaw fusion to get the fused half jaw, (2) Individual tooth fusion to get each fused individual tooth.

The half-jaw fusion algorithm is illustrated in Algorithm 4 (Data S4). We construct the KD-tree with the IOS data, and compute the distance from each point in the CBCT to the KD-tree. To remove the points corresponding to the CBCT crown, we remove points with top $d(d = 30\%)$ distance in the CBCT point cloud. However, some independent noise (including noise points and noise clusters) may be introduced in the mesh reconstruction process. Therefore, the outlier algorithm and the DBSCAN clustering algorithm are adopted for deleting those clusters and noise points [7]. The DBSCAN algorithm is a high-density-based clustering non-parametric algorithm that can delete some abnormal debris clusters with less than $k(k = 2000)$ point clouds. Finally, the oral scan is integrated with the remaining points in the CBCT, and Poisson reconstruction (Depth=8) is performed to generate the rough fusion results. Laplacian filtering algorithm (*Iter* = 6) is applied to obtain the final fusion result.

After we get the fused half jaw, we further integrate the tooth roots with the IOS mesh crown to get each tooth. By doing so we get each tooth with FDI notations. Specifically, we merge the remaining points in the CBCT to its corresponding IOS tooth crown by computing the Euclidean distance and integrating CBCT points to the nearest IOS tooth crown.

CBCT image segmentation metrics: To objectively evaluate the results obtained from different CBCT segmentation methods, four representative fusion quality metrics are adopted in this paper [8], using the default parameters provided in a corresponding study. The larger values of these metrics, the better segmentation results. More details regarding these metrics are as follows.

1) IoU

The Intersection over Union (IoU) is defined as the area of intersection between the predicted segmentation map A and the ground truth map B, divided by the area of the union between the two maps, and ranges between 0 and 1:

$$\mathrm{IoU} = J(A, B) = \frac{|A \cap B|}{|A \cup B|}.$$

2) Dice

The Dice coefficient, commonly used in medical image analysis, can be defined as twice the overlap area of the predicted and ground-truth maps divided by the total number of pixels:

$$\mathrm{Dice} = \frac{2|A \cap B|}{|A| + |B|}.$$

3) Recall / Precision

The Recall / Precision scores can be defined for each class, as well as at the aggregate level, as follows:

$$\mathrm{Recall} = \frac{\mathrm{TP}}{\mathrm{TP} + \mathrm{FN}}, \qquad \mathrm{Precision} = \frac{\mathrm{TP}}{\mathrm{TP} + \mathrm{FP}},$$

where TP refers to the true positive fraction, FP refers to the false positive fraction, and FN refers to the false negative fraction.

IOS mesh segmentation metrics

1) ACC_a

ACC_a means the average-area accuracy. Given a jaw mesh $M = \{m_i\}, i = 1\ to\ N$ with N faces, the ACC_a is defined as the ratio of the total area of correctly predicted faces to the whole faces :

$$ACC_a = \frac{\sum_{i=1}^{N} \Phi(y_i, \hat{y}_i) \cdot a_i}{\sum_{i=1}^{N} a_i},$$

$$\Phi(y_i, \hat{y}_i) = \begin{cases} 1, & if\ y_i = \hat{y}_i, \\ 0, & otherwise. \end{cases},$$

where a_i denotes the area for face m_i, $\hat{y}_i$ and y_i denotes the predicted label and the gold label of face m_i, respectively, and $\Phi(\cdot)$ is the indicator function.

2) ACC_f

ACC_f means the per-face accuracy. Similarly, given a jaw mesh $M = \{m_i\}, i = 1\ to\ N$ with *N* faces, the ACC_f is defined as the ratio of the number of correctly predicted faces to the whole faces:

$$ACC_f = \frac{1}{N} \sum_{i=1}^{N} \Phi(y_i, \hat{y}_i),$$

where the meanings of $\hat{y}_i$, y_i and $\Phi(\cdot)$ are the same as above.

Multimodal Fusion metrics: We evaluate the multimodal fusion result by computing the error between the results of DDMF and ground truth. We utilize three distance-based metrics to measure the geometric error, namely, Chamfer distance (CD) [9], average absolute distance (ASSD) [10], and Hausdorff distance (HD) [10]. The CD measures the mean distance between two sets of vertices. ASSD and HD compute the average and maximum distance between two sets of 10k sampled points from surface meshes.

The ASSD is defined as follows :

$$ASSD\left(S_R, S_A\right) = mean\left\{mean\left\{dist\left(a, S_R\right), a \in S_A\right\}, mean\left\{dist\left(r, S_A\right), r \in S_R\right\}\right\}.$$

S_R and *S_A* are the surfaces of objects of gold standard and algorithm segmentation, respectively, dist(a, *S_R*) is the nearest Euclidean distance from a surface point a to the surface *S_R*, and mean {•} is the arithmetical average operator.

The Hausdorff Distance (*HD*) between two finite point sets *A* and *B* is defined by:

$$HD(A, B) = \max(h(A, B), h(B, A)),$$

where *h(A,B)* is called the directed Hausdorff distance and given by

$$h(A, B) = \max_{a \in A} \min_{b \in B} ||a - b||,$$

where ||a−b|| is Euclidean distance between a and b.

The Chamfer distance between S_1, S_2 is defined as follow:

$$d_{CD}(S_1, S_2) = \sum_{x \in S_1} \min_{y \in S_2} \|x - y\|_2^2 + \sum_{y \in S_2} \min_{x \in S_1} \|x - y\|_2^2 .$$

For each point, the d_{CD} finds the nearest neighbor in the other set and sums the squared distances up. Viewed as a function of point locations in S_1 and S_2, CD is continuous and piecewise smooth. The range search for each point is independent.

Comparison with Baselines

1) CBCT image segmentation

We introduce both state-of-the-art CNNs (Unet [11] , Unet++ [12] , FCN [13] , and DeepLabv3 [14]) and recently proposed transformer models(MedT [15], UctransNet [16], Swin transformer [17]). The backbone of DeepLabv3 and FCN is ResNet-50 [18]. The Swin Transformer is pre-trained with Imagenet-22k. DeepLabv3, FCN, and standard Swin Transformer are based on the public code at mmsegmentation [4], and the training hyper parameters are in mmsegmentation by default. The implementations of Swin, MedT, UCTransNet, UNet, UNet++ are based on their original source codes. The training is conducted with 4 Nvidia RTX 3090 GPUs and Pytorch 1.8.0 for comparison experiments, and the training procedure lasts for 160K iterations.

Supplemental References

1. Xie, Y., Zhang, J., Xia, Y., and Wu, Q. (2022). UniMiSS: Universal Medical Self-supervised Learning via Breaking Dimensionality Barrier. In Computer Vision–ECCV 2022: 17th European Conference, Tel Aviv, Israel, October 23–27, 2022, Proceedings, Part XXI (Springer), pp. 558–575.
2. Cui, Z., Fang, Y., Mei, L., Zhang, B., Yu, B., Liu, J., Jiang, C., Sun, Y., Ma, L., and Huang, J. (2022). A fully automatic AI system for tooth and alveolar bone segmentation from cone-beam CT images. Nat. Commun. *13*, 1–11.
3. Zhang, Y., Liao, Q., Ding, L., and Zhang, J. (2022). Bridging 2D and 3D segmentation networks for computation-efficient volumetric medical image segmentation: An empirical study of 2.5 D solutions. Comput. Med. Imaging Graph. *99*, 102088.
4. Xu, J., Chen, K., and Lin, D. (2020). MMSegmenation.
5. Rusu, R.B., Blodow, N., and Beetz, M. (2009). Fast point feature histograms (FPFH) for 3D registration. In 2009 IEEE international conference on robotics and automation (IEEE), pp. 3212–3217.
6. Besl, P.J., and McKay, N.D. (1992). Method for registration of 3-D shapes. In Sensor fusion IV: control paradigms and data structures (Spie), pp. 586–606.
7. Zhou, Q.-Y., Park, J., and Koltun, V. (2018). Open3D: A modern library for 3D data processing. ArXiv Prepr. ArXiv180109847.
8. Minaee, S., Boykov, Y.Y., Porikli, F., Plaza, A.J., Kehtarnavaz, N., and Terzopoulos, D. (2021). Image segmentation using deep learning: A survey. IEEE Trans. Pattern Anal. Mach. Intell.
9. Fan, H., Su, H., and Guibas, L.J. (2017). A point set generation network for 3d object reconstruction from a single image. In Proceedings of the IEEE conference on computer vision and pattern recognition, pp. 605–613.
10. Cruz, R.S., Lebrat, L., Bourgeat, P., Fookes, C., Fripp, J., and Salvado, O. (2021). Deepcsr: A 3d deep learning approach for cortical surface reconstruction. In Proceedings of the IEEE/CVF Winter Conference on Applications of Computer Vision, pp. 806–815.
11. Ronneberger, O., Fischer, P., and Brox, T. (2015). U-net: Convolutional networks for biomedical image segmentation. In International Conference on Medical image computing and computer-assisted intervention (Springer), pp. 234–241.
12. Zhou, Z., Siddiquee, M.M.R., Tajbakhsh, N., and Liang, J. (2019). Unet++: Redesigning skip connections to exploit multiscale features in image segmentation. IEEE Trans. Med. Imaging *39*, 1856–1867.
13. Long, J., Shelhamer, E., and Darrell, T. (2015). Fully convolutional networks for semantic segmentation. In Proceedings of the IEEE conference on computer vision and pattern recognition, pp. 3431–3440.

14. Chen, L.-C., Papandreou, G., Schroff, F., and Adam, H. (2017). Rethinking atrous convolution for semantic image segmentation. ArXiv Prepr. ArXiv170605587.
15. Valanarasu, J.M.J., Oza, P., Hacihaliloglu, I., and Patel, V.M. (2021). Medical transformer: Gated axial-attention for medical image segmentation. In International Conference on Medical Image Computing and Computer-Assisted Intervention (Springer), pp. 36–46.
16. Wang, H., Cao, P., Wang, J., and Zaiane, O.R. (2022). Uctransnet: rethinking the skip connections in u-net from a channel-wise perspective with transformer. In Proceedings of the AAAI Conference on Artificial Intelligence, pp. 2441–2449.
17. Liu, Z., Lin, Y., Cao, Y., Hu, H., Wei, Y., Zhang, Z., Lin, S., and Guo, B. (2021). Swin transformer: Hierarchical vision transformer using shifted windows. In Proceedings of the IEEE/CVF International Conference on Computer Vision, pp. 10012–10022.
18. He, K., Zhang, X., Ren, S., and Sun, J. (2016). Identity mappings in deep residual networks. In European conference on computer vision (Springer), pp. 630–645.
19. Isensee, F., Jaeger, P.F., Kohl, S.A., Petersen, J., and Maier-Hein, K.H. (2021). nnU-Net: a self-configuring method for deep learning-based biomedical image segmentation. Nat. Methods *18*, 203–211.
