# Supplementary material for: Deep learning-enabled 3D multimodal fusion of cone-beam CT and intraoral mesh scans for clinically applicable tooth-bone reconstruction
Source: Patterns (N Y). 2023 Aug 15;4(9):100825. doi: 10.1016/j.patter.2023.100825 (PMC10499902; doi:10.1016/j.patter.2023.100825)
Supplement: Data S1. Curvature-based segmentation [file mmc2.pdf]

Data S1. Curvature-based Segmentation

Algorithm 1 Curvature-based Segmentation ($CurSeg(M, L, T)$)

```
M represents the teeth mesh. T ∈ (0,1) represents the threshold for curvature segmentation. Set the number of vertices and faces of M as N_v and N_f, and the L level neighbors set of vertex V_i as U_i, where i ∈ [1, N_v]. Set the set of curvature for each vertices as C := {C_1, C_2, ..., C_{N_v}}
N ← empty point cloud
for i := 1, 2, ..., N_v do
    C_i ← (1/K) Σ_{U_i} arccos(V_i · U_i^k), where k ∈ [1, K]
end for
V_T ← the set of vertices with largest N_v T curvatures from C
Remove V_T from M
for i := 1, 2, ..., N_f do
    if F_i has vertex in V_T then
        Remove F_i from M
    end if
end for
return M, V_T
```
