# Supplementary material for: Deep learning-enabled 3D multimodal fusion of cone-beam CT and intraoral mesh scans for clinically applicable tooth-bone reconstruction
Source: Patterns (N Y). 2023 Aug 15;4(9):100825. doi: 10.1016/j.patter.2023.100825 (PMC10499902; doi:10.1016/j.patter.2023.100825)
Supplement: Data S2. Merge process [file mmc3.pdf]

Data S2. Merge process

Algorithm 2 Merge ($Merge(M, N, T_{iter})$)

$M := [M_1, M_2, \dots, M_{N_t}]$ represents the teeth points, where $M_{i \in [1, N_t]}$ represents the i_{th} tooth
$N := [V_1, V_2, \dots, V_{N_r}]$ represents the removed points. T_{iter} represents the total merge steps
$N_T \leftarrow \frac{N}{T_{iter}}$
for $t := 1, 2, \dots, T_{iter}$ **do**
    for $i := 1, 2, \dots, N_t$ **do**
        $tree_i \leftarrow$ k-d tree based on M_i
        for $j := 1, 2, \dots, N_r$ **do**
            $D_{ij} \leftarrow$ the distance from V_j to $tree_i$
        end for
    end for
    for $j := 1, 2, \dots, N_r$ **do**
        $D_j \leftarrow \min_i D_{ij}$
        $k_j \leftarrow \arg\min_i D_{ij}$
    end for
    $V_T \leftarrow$ set of N_T removed vertices with smallest D
    Remove V_T from N
    for $j := 1, 2, \dots, N_T$ **do**
        merge $V_T^j \in V_T$ to M_{k_j}
    end for
end for
return M
