# Supplementary material for: Deep learning-enabled 3D multimodal fusion of cone-beam CT and intraoral mesh scans for clinically applicable tooth-bone reconstruction
Source: Patterns (N Y). 2023 Aug 15;4(9):100825. doi: 10.1016/j.patter.2023.100825 (PMC10499902; doi:10.1016/j.patter.2023.100825)
Supplement: Data S3. Curvature-based segmentation on 3D CBCT mesh [file mmc4.pdf]

Data S3. Curvature-based Segmentation on 3D CBCT mesh

Algorithm 3 Curvature-based Segmentation on 3D CBCT Mesh $Seg(M, L, T_c, T_{iter}))$

M represents the CBCT data. $T_c \in (0, 1)$ and L represent the threshold and the level of neighbors used in the curvature segmentation. T_{iter} represents the total number of merge steps. N_t represents the estimated number of teeth. T_{size} represents the maximum number of point clouds for a single tooth

$M, N \leftarrow CurSeg(M, L, T_c)$
$M \leftarrow$ [M$_1$,M_2,...,M_{N_t}], where $M_{i\in[1,N_t]}$ is the connected component from M
while size($M_{i\in[1,N_t]}$) $> T_{size}$ **do**
    M_i,N_i := $CurSeg(M_i)$
    $M_i \leftarrow [M_1$,M_2,...,$M_{N_t^i}]$, where $M_{j\in[1,N_t^i]}$ is the connected component from M_i
    $M := M + M_i$
    $N := N + N_i$
end while
$M := Merge(M, N, T_{iter})$
$Low \leftarrow$ the lowest z-axis among all teeth in M
$Up \leftarrow$ the largest z-axis among all teeth in M
$N_t \leftarrow$ size of M
$Maxilla \leftarrow$ empty list
$Mandible \leftarrow$ empty list
for $i := 1, 2, \ldots, N_t$ **do**
    $Centro_i \leftarrow$ centroid of M_i, where $Centro_i := [Centro_i^x, Centro_i^y, Centro_i^z]$
    if $Centro_i^z$ -Low > Up-$Centro_i^z$ **then**
        $Maxilla$ += M_i
    else
        $Mandible$ += M_i
    end if
end for
return $Maxilla$,$Mandible$
