# Supplementary material for: Deep learning-enabled 3D multimodal fusion of cone-beam CT and intraoral mesh scans for clinically applicable tooth-bone reconstruction
Source: Patterns (N Y). 2023 Aug 15;4(9):100825. doi: 10.1016/j.patter.2023.100825 (PMC10499902; doi:10.1016/j.patter.2023.100825)
Supplement: Data S4. Tooth crown replacement [file mmc5.pdf]

Data S4. Tooth crown replacement

Algorithm 4 Tooth crown replacement algorithm ($CrownsReplacement(\mathcal{M}_C, \mathcal{M}_I, K, Iter)$)

1: $\mathcal{M}_C \in R^{N_m \times 3}$ and $\mathcal{M}_I \in R^{N_n \times 3}$ represent the CBCT and IOS point clouds respectively, where N_m and N_n are the points number in each point cloud. Set the the threshold of DBSCAN as K, and the iteration steps of Laplacian filter as $Iter$
2: Construct KDTree from $\mathcal{M}_I$
3: Calculate the Euclidean distance between $\mathcal{M}_C$ and KDTree
4: Sort($\mathcal{M}_C$,Euclidean distance)
5: Delete d ($d < N_m$) nearest neighbor points to KDTree from $\mathcal{M}_C$
6: Delete noise and clusters($points < K$) from $\mathcal{M}_C$ with Local Outlier Factor technique and DBSCAN
7: $P \leftarrow \mathcal{M}_C + \mathcal{M}_I$
8: Poisson reconstruction(P, $Depth$)
9: **for** $i := 1, 2, \ldots, Iter$ **do**
10: $\quad P$:= Laplacian filter(P_i)
11: **end for**
12: **return** P
