# Supplementary material for: Deep learning-enabled 3D multimodal fusion of cone-beam CT and intraoral mesh scans for clinically applicable tooth-bone reconstruction
Source: Patterns (N Y). 2023 Aug 15;4(9):100825. doi: 10.1016/j.patter.2023.100825 (PMC10499902; doi:10.1016/j.patter.2023.100825)
Supplement: Document S2. Article plus supplemental information [file mmc8.pdf]

Patterns

Article

Deep learning-enabled 3D multimodal fusion of cone-beam CT and intraoral mesh scans for clinically applicable tooth-bone reconstruction

Highlights

- Identified CBCT and IOS fusion for 3D tooth-bone reconstruction in clinical applications
- Proposed deep multimodal fusion pipeline to integrate both CBCT and IOS modalities
- Proposed novel techniques for accurate CBCT and IOS segmentation and 3D mesh fusion
- Collected multimodal dataset, validated method, demonstrated real-world applicability

Authors

Jiaxiang Liu, Jin Hao,
Hangzheng Lin, ..., Zuolin Jin,
Zhihe Zhao, Zuozhu Liu

Correspondence

zhzhao@scu.edu.cn (Z.Z.),
zuozhuliu@intl.zju.edu.cn (Z.L.)

In brief

The DDMF framework offers accurate and efficient tooth-bone analysis in virtual dental treatment planning. Dentists benefit from improved segmentation and reconstruction of crown-root-bone structures. The study showcases the potential of deep learning in digital dentistry and inspires innovations in medical imaging. Overall, it advances dental healthcare by enhancing treatment planning and decision making, saving time for dental professionals and improving patient outcomes.

Liu et al., 2023, Patterns *4*, 100825
September 8, 2023 © 2023 The Authors.
https://doi.org/10.1016/j.patter.2023.100825

CellPress

Patterns

CellPress
OPEN ACCESS

Article

Deep learning-enabled 3D multimodal fusion of cone-beam CT and intraoral mesh scans for clinically applicable tooth-bone reconstruction

Jiaxiang Liu,[1,2,4,11] Jin Hao,[3,5,11] Hangzheng Lin,[2] Wei Pan,[6] Jianfei Yang,[7] Yang Feng,[8] Gaoang Wang,[2] Jin Li,[9] Zuolin Jin,[10] Zhihe Zhao,[3,*] and Zuozhu Liu[1,2,12,*]

[1]Stomatology Hospital, School of Stomatology, Zhejiang University School of Medicine, Zhejiang Provincial Clinical Research Center for Oral Diseases, Hangzhou 310000, China
[2]Zhejiang University-University of Illinois at Urbana-Champaign Institute, Zhejiang University, Haining 314400, China
[3]State Key Laboratory of Oral Diseases & National Center for Stomatology & National Clinical Research Center for Oral Diseases & West China Hospital of Stomatology, Sichuan University, Chengdu 610041, China
[4]College of Computer Science and Technology, Zhejiang University, Hangzhou 310058, China
[5]Harvard School of Dental Medicine, Harvard University, Boston, MA 02115, USA
[6]OPT Machine Vision Tech Co., Ltd., Tokyo 135-0064, Japan
[7]School of Electrical and Electronic Engineering, Nanyang Technological University, Singapore 639798, Singapore
[8]Angelalign Inc., Shanghai 200433, China
[9]Department of Stomatology, The First Affiliated Hospital of Shenzhen University, Shenzhen Second People's Hospital, Shenzhen 518025, China
[10]Department of Orthodontics, School of Stomatology, Air Force Medical University, Xi'an 710032, China
[11]These authors contributed equally
[12]Lead contact
*Correspondence: zhzhao@scu.edu.cn (Z.Z.), zuozhuliu@intl.zju.edu.cn (Z.L.)
https://doi.org/10.1016/j.patter.2023.100825

THE BIGGER PICTURE The integration of digital dentistry with artificial intelligence holds significant importance. In the field of oral healthcare, various modalities of data provide complementary information. However, the manual fusion process required by dentists often consumes a significant amount of time, hindering precise reconstruction, diagnosis, and treatment simulation. To address this issue, we propose a multimodal fusion system that automates the fusion of diverse three-dimensional dental medical imaging modalities, enabling efficient and accurate tooth crown-root-bone reconstruction and analysis, thus serving as a powerful tool for oral healthcare practitioners. The application of this system holds paramount clinical significance, enhancing diagnostic accuracy, reducing treatment costs, and improving overall oral health outcomes for patients.

1 2 3 4 5 **Proof-of-Concept:** Data science output has been formulated, implemented, and tested for one domain/problem

SUMMARY

High-fidelity three-dimensional (3D) models of tooth-bone structures are valuable for virtual dental treatment planning; however, they require integrating data from cone-beam computed tomography (CBCT) and intraoral scans (IOS) using methods that are either error-prone or time-consuming. Hence, this study presents Deep Dental Multimodal Fusion (DDMF), an automatic multimodal framework that reconstructs 3D tooth-bone structures using CBCT and IOS. Specifically, the DDMF framework comprises CBCT and IOS segmentation modules as well as a multimodal reconstruction module with novel pixel representation learning architectures, prior knowledge-guided losses, and geometry-based 3D fusion techniques. Experiments on real-world large-scale datasets revealed that DDMF achieved superior segmentation performance on CBCT and IOS, achieving a 0.17 mm average symmetric surface distance (ASSD) for 3D fusion with a substantial processing time reduction. Additionally, clinical applicability studies have demonstrated DDMF's potential for accurately simulating tooth-bone structures throughout the orthodontic treatment process.

This is an open access article under the CC BY license (http://creativecommons.org/licenses/by/4.0/).

INTRODUCTION

Digital technology is expected to change every aspect of modern dentistry, from virtual treatment planning to remote patient management. In particular, digital cone-beam computed tomography (CBCT) models have accelerated treatment planning and management in dental practice such as orthodontics and implant surgery. One advantage of these CBCT models is that they can illustrate the complex anatomical structures of both teeth and bones, creating a virtual model of the head.[1,2] Recent advances in deep-learning-based automatic segmentation methods have enabled the segmentation of tooth-bone structures in CBCT images[3–7] for digital model visualization. However, the clinical applicability of the segmented CBCT images remains limited. Previous research has shown that CBCT measurements can be up to 6.9% smaller than the actual values of objects, depending on the resolutions.[8,9] This CBCT value discrepancy, termed shrinkage, makes the digital model unreliable for the precise simulation of implant surgery and orthodontic outcomes, suggesting the necessity of data matching between CBCT and other modality measurements in clinical practice. In addition, the accurate capture of occlusal surface information is of high importance in many clinical applications, as the design of saw, drilling, and orthognathic positioning guides is primarily dependent on occlusal surfaces.[10–12] Nevertheless, CBCT often fails to recapitulate accurate information on occlusal surfaces because of the high density of enamel, dental restorations, implants, and orthodontic appliances.[13–15] Furthermore, the correct anatomical position of dentition in the maxilla and mandible is often missing from CBCT images, making orthodontic treatment simulation impossible using CBCT alone.

Compared with CBCT, intraoral scanners (IOSs) are extensively used in dentistry to generate a digital impression of the tooth's anatomy by projecting a light source onto the dental arches. An IOS scan is typically represented as a mesh with 150,000–350,000 triangular faces, each denoting a specific tooth or the gingiva. Recently, several deep-learning-based methods have been proposed for the automatic segmentation of IOS meshes.[16–20] Although IOS meshes only provide information on the tooth crown and gingiva, their shrinkage ratio can be as low as 0.9%, making them the most accurate models in three-dimensional (3D) digital modalities. In addition, IOSs are sufficiently accurate for capturing occlusal information and dentition position.[21] In practice, the fusion of both CBCT and IOS, that is, integrating comprehensive 3D CBCT models (complete tooth-bone structures) with high-resolution tooth crowns and accurate occlusal information from IOS, could provide crown-root-bone structures with the accuracy required for many dental applications.

Previous studies have focused on the fusion of CBCT and IOS for clinical applications.[22,23] However, there are still many challenges in the fusion of CBCT and IOS, and a fully automatic, efficient, and clinically applicable solution has yet to be developed. First, the accurate segmentation of CBCT and IOS, which represents an indispensable prerequisite in current manual fusion solutions, is a non-trivial task because of the complicated anatomic and morphological features of different patients. Although recent research has highlighted the effectiveness of deep-learning methods for the accurate and automatic segmentation of CBCT and IOS, their performance could be further improved for clinically applicable tooth identification and multimodal fusion (MF). Second, the automatic fusion of both modalities remains an open and poorly defined task. Specifically, although the IOS provides clear half-jaw tooth crowns and occlusal information, CBCT models usually consider different bite positions without available separated jaws. Hence, the contacts in the maxilla and mandible, connected boundaries between adjacent teeth, and shape variance between reconstructed 3D CBCT models and IOS scans present great difficulties for an accurate and efficient MF and the delineation of individual teeth. Finally, the lack of a large-scale, real-world dataset for such MF tasks, which is crucial for the careful and systematic testing of these types of data fusion methods, represents another significant challenge.

Therefore, in this study, we aimed to resolve the aforementioned challenges by introducing a fully automatic system that efficiently generates accurate crown-root-bone structures by fusing IOS mesh and CBCT image data with deep-learning methods. To achieve this, our model was trained on a large-scale dataset with 503 CBCT and 28,559 IOS meshes, manually annotated by human experts. For CBCT segmentation, our framework achieved an average Dice coefficient of 93.99%, significantly outperforming the baselines. For IOS segmentation, using a test dataset of 200 IOS meshes, our model achieved mean intersection over union (mIoU) values of 93.07% and 95.70% for the maxilla and mandible, outperforming state-of-the-art methods by 1.77% and 3.52%, respectively. For MF, Deep Dental MF (DDMF) showed a 0.47 mm Hausdorff distance (HD)[24] between the model and ground truth for the entire set of teeth from 20 cases, which was 0.21 mm lower than that of CBCT reconstruction. The pipeline of the DDMF can be observed in Figure 1. In addition, the DDMF framework required 20–25 min to generate the fused model, compared with the duration of over 5 h required by human experts assisted by semi-automatic tools.

RESULTS

Participants

We conducted experiments on a large-scale, real-world dataset that contained 503 samples with both CBCT and IOS, and an extra dataset of 28,559 IOS meshes collected from hospitals and clinics in 25 provinces in China from 2018 to 2021. The 503 patients all had malocclusion and aged between 9 and 48 years, with 32.5% being males and 67.5% being females, respectively. The CBCT data were acquired using different types of equipment, with resolutions ranging from 0.125 to 0.5 mm. The CBCT images and IOS meshes were annotated by a group of human experts.

CBCT segmentation results

We conducted a 5-fold cross-validation test, each with a hold-out set of CBCT images from 50 patients, to evaluate the segmentation performance of the proposed CBCT segmentation module, termed Tooth Swin Transformer Network (TSTNet), as shown in Figure 2. To establish a robust baseline, we compared TSTNet with several widely used and state-of-the-art segmentation networks as baselines and fine-tuned them for the best performance, such as UNet,[25] UNet++,[26] Deeplabv3,[27] FCN,[28] Medical Transformer (MedT),[29] UCTransNet,[30] and the standard Swin Transformer.[31] We did not conduct comparisons with baselines that required 3D voxel annotations[7,32] as the task definition was

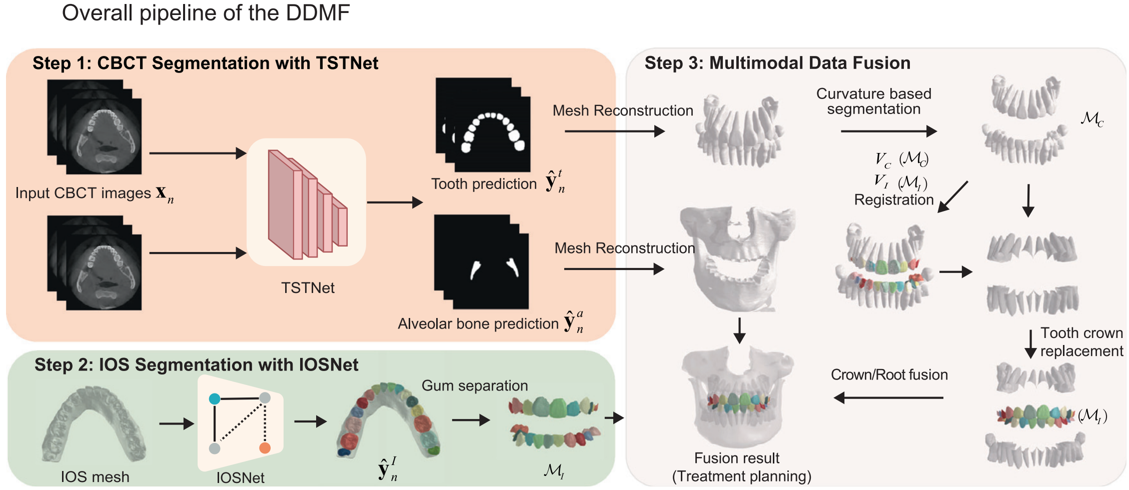

Figure 1. Overall pipeline of the DDMF framework

Step 1: the pipeline of CBCT image segmentation for tooth and alveolar bone with TSTNet. Step 2: the pipeline of IOS segmentation with IOSNet and post-processing to generate FDI tooth codes. Step 3: the pipeline of the multimodal fusion module that fuses the reconstructed CBCT 3D meshes and IOS meshes with point curvature feature-based half-jaw segmentation, registration, and 3D fusion methods.

different and their codes and datasets were not publicly available. The Swin, MedT, UCTransNet, UNet, and UNet++ networks were implemented based on their original source codes, while the implementations of FCN and Deeplabv3 were based on the codes from MMsegmentation.[33] To ensure that our evaluation accurately reflected the CBCT segmentation performance, we restored the original resolutions of the CBCT data for the 5-fold test.

The overall segmentation results are listed in Table 1. TSTNet achieved an average Dice coefficient and IoU of 93.99% and 88.68% on the 5-fold test, respectively, outperforming all the baselines. We noticed that a well-tuned UNet or UNet++ could already achieve a relatively good performance on this two-dimensional (2D) tooth segmentation task, and the standard Swin Transformer further boosted the performance. In addition, TSTNet consistently outperformed the state-of-the-art Swin model, with a performance gain of 1.05% IoU and 0.70% Dice coefficient. Considering the similar performance across all baselines, the performance gain was sufficiently significant, as illustrated in Figure 3. Moreover, as shown in Figure 4B, the precision/recall curve of TSTNet with the baselines indicated that TSTNet exhibited the best performance. Additional results are shown in Tables S1 and S5.

The superior performance of TSTNet is also convincingly demonstrated by the visualizations shown in Figure 3A. Our TSTNet method showed fewer false positive (FP) and false negative (FN) errors than the baselines. For instance, the baselines might incorrectly recognize a large portion of the background as erupted third molars (case a), fail to identify the large incisor (case a), fail to recognize hyperdontia (case b), or fail to distinguish the ambiguous boundaries among tiny tooth slices (case c). By contrast, TSTNet generated considerably better results for these complex cases. A similar performance could also be observed in the jaw segmentation results, as in cases d and e. Further visualizations can be found in Figures S3, S4, and S7.

IOS segmentation results

The performance was also compared with classical point cloud segmentation networks (i.e., PointNet,[34] PointNet++,[35] and DGCNN[36]) and strong IOS segmentation CNN baselines in Xu et al.[17] (i.e., MeshSegNet,[37] DCNet,[16] and TSGCNet[38]), all following their original settings and source codes. We evaluated the performance of IOSNet on the same test set as in Hao et al.[16] with 200 meshes from 100 patients. We reported the mIoU, perface accuracy, and average-area accuracy as in Hao et al.[16] for comparison. Additional visualizations are shown in Figure S5.

The results are reported in Table 2. The IOSNet achieved an mIoU of 93.70% and 95.70% on maxillary and mandibular IOS scans, respectively, outperforming the baselines by 1.77% and 3.52%. Figure 3B illustrates two cases. The results demonstrated that IOSNet could generate segmentations nearly identical to the ground truth; that is, it did not recognize two incisors as one tooth (case a) and could recognize the third molar and generate better tooth-gingiva and tooth-tooth boundaries (cases a and b). Given that DCNet could generate clinically applicable results for most cases,[16] such a significant improvement in mIoU could corroborate the excellent performance of IOSNet. We do not provide too many visualizations here. The performance of IOSNet was subsequently demonstrated through real-world clinical validation using our DDMF framework. Additional results are reported in Table S2.

Multimodal fusion results

To evaluate the MF achieved by our DDMF framework, we computed the average symmetric surface distance (ASSD),[24] HD,[24] and Chamfer distance (CD) metrics[39] between the fused outputs and ground truth for 50 patients. Notably, the ground truth fused models were provided by a committee of human experts and were ensured to be clinically applicable because they were successfully used in clinical practice. Table 3 reports the fusion error for seven different teeth categories as well as the

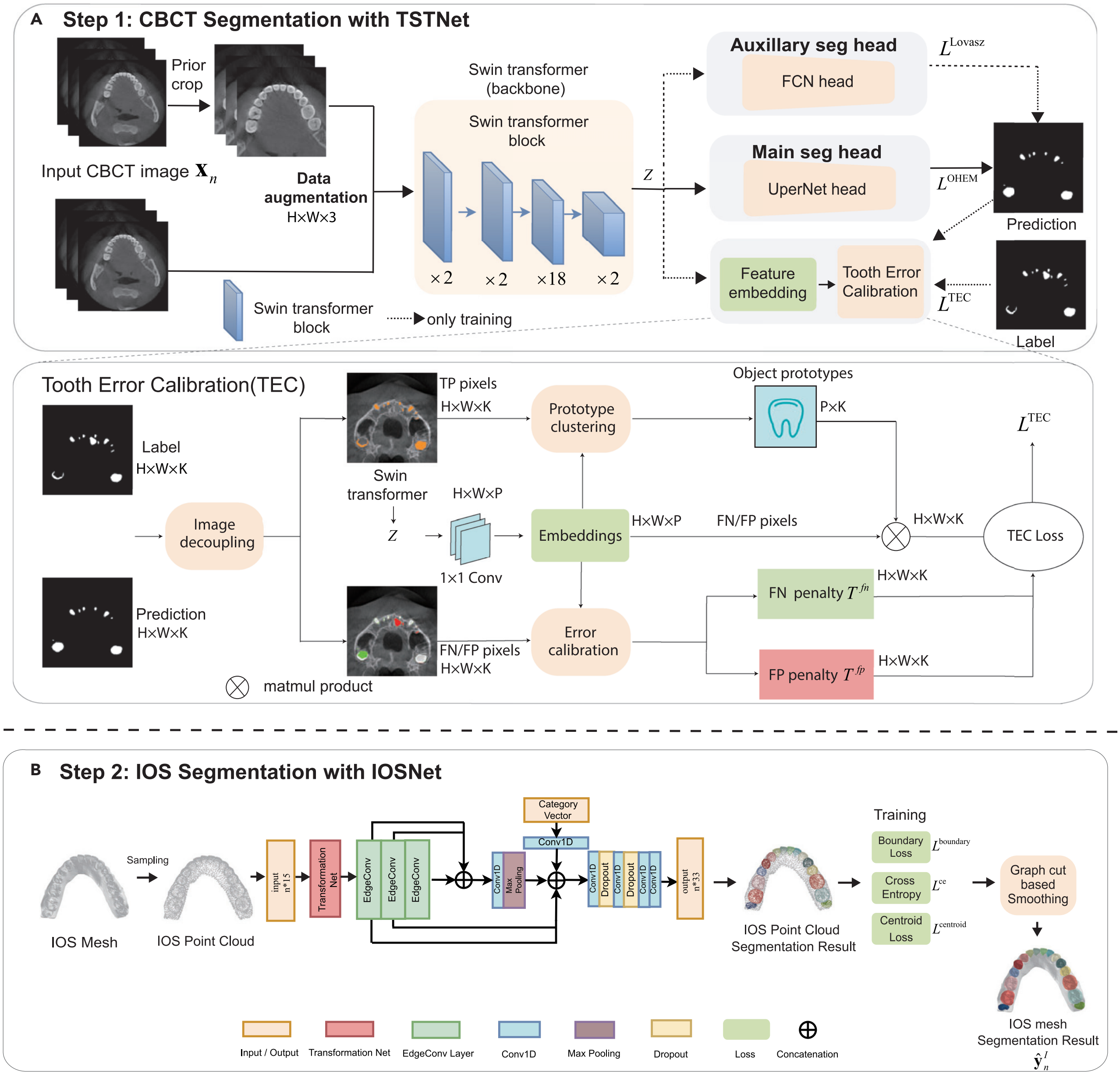

Figure 2. The pipeline of TSTNet for tooth and alveolar bone with TSTNet and the pipeline IOSNet
(A) The pipeline of CBCT image segmentation for tooth and alveolar bone with TSTNet.
(B) The pipeline of IOS segmentation with IOSNet and post-processing to generate FDI tooth codes.

overall fusion performance. We excluded third molars because many patients did not have third molars in our MF dataset. The results of these intermediate steps are described in the following sections. We did not investigate any baselines here as there are no systematic solutions for this challenging task. However, we conducted a clinical applicability study to test the effectiveness of our method.

We noticed that the corresponding average distances could be as small as 0.17, 0.20, and 0.37 mm for ASSD, CD, and HD, respectively with consistently small SDs. The errors were small and consistent across different teeth categories. The relatively large errors in the 1st and 2nd molars were due mainly to these two molars' having larger volumes. Nonetheless, considering that the resolution of CBCT machines ranged between 0.125 and 0.5 mm, a distance error as low as one to two pixels would be acceptable for numerous clinical applications, as validated by human experts and demonstrated by the clinical applicability test. More detailed MF results are presented in Tables S3 and S4.

In this regard, four cases of MF are visualized in Figure 4, with additional visualizations included in Figure S6. By fusing CBCT and IOS, instance-level 3D teeth could be automatically generated by incorporating the Fédération Dentaire Internationale

CellPress
OPEN ACCESS

Table 1. Five-fold cross-validation segmentation results on CBCT (each tested with 50 patients)

Model	IoU, %	Dice coefficient, %	Recall, %	Precision, %
UNet	86.90 ± 1.06	92.95 ± 0.61	92.83 ± 2.74	93.23 ± 1.57
UNet++	86.83 ± 1.16	92.95 ± 0.66	93.40 ± 1.66	92.51 ± 0.60
FCN	85.68 ± 1.60	92.28 ± 0.92	92.21 ± 2.31	92.39 ± 0.48
Deeplabv3	85.68 ± 1.61	92.28 ± 0.93	92.05 ± 2.34	92.54 ± 0.53
MedT	73.39 ± 5.97	84.52 ± 3.81	75.00 ± 5.87	97.01 ± 1.01*
UCTransNet	84.08 ± 0.80	91.35 ± 0.47	97.95 ± 0.62*	85.59 ± 0.73
Swin	87.61 ± 1.19#	93.29 ± 0.67#	93.95 ± 1.68#	92.95 ± 0.54
TSTNet	88.68 ± 2.15*	93.99 ± 1.20*	92.37 ± 2.78	95.71 ± 0.70#

IoU, intersection over union. *Best performance and #Second-Best performance. Values denote mean ± SD on cross-validation.

(FDI) tooth number information from the IOS, as shown in Figure 4A, thus eliminating the need for performing the time-consuming task of providing instance-level annotations in CBCT slices. As shown in Figures 4A and 4B, the magnified tooth-tooth boundaries also reveal the existence of tooth adhesion in the CBCT reconstructions, while the fused results were nearly identical to the ground truth. Furthermore, we visualized both teeth and bones and examined the tooth-bone relationships in each case. As shown in Figures 4A–4D, our findings demonstrated that DDMF could accurately identify critical tooth-bone boundaries such as the relative positions between the tooth roots, and alveolar bones were clearly recognized in all cases. Hence, these results can be employed for high-fidelity 3D simulations of treatment plans. Finally, we randomly selected several teeth and placed the fused results and ground truth in the same position with the same orientation to identify the differences. From Figures 4A–4E, there were very small surface deviations, as indicated by the light pink areas, which were consistent with the small ASSD and CD errors. Furthermore, CD, ASSD, and HD were calculated for the entire set of teeth from the 20 cases to compare the variations between CBCT reconstructions and DDMF outcomes. Figures 4C–4E display the statistical findings. It can be observed that the DDMF reduced the prediction error, thus demonstrating its effectiveness.

Ablation on the effectiveness of data augmentation, TEC, and loss

An ablation study was conducted to evaluate the effectiveness of the proposed TSTNet. The Swin Transformer was used as the baseline and only achieved an IoU of 90.52%, on a development set of 43 patients. In contrast, our TSTNet achieved an IoU of 93.22%, while each novel component, including prior-knowledge-based data augmentation, the TEC component, and the integrated loss function, significantly contributed to the enhanced performance, as illustrated in Table 4.

Ablation on the effectiveness of point curvature feature

We also discuss the effectiveness of the proposed curvature-based algorithm for separating the upper and lower jaws, with experiments conducted on 50 patients, 31 of whom were in a closed-biting position with tight tooth contacts. Remarkably, our method successfully separated 94% of the upper and lower jaws, whereas traditional segmentation algorithms based on the Gaussian curvature and DBSCAN[40] separated only 42% and 24% of them, respectively, as shown in Table 5.

Clinical utility evaluation

We assessed the clinical utility of our method by computing the end-to-end inference time of each module and the DDMF framework (see Tables 6 and S1). TSTNet took approximately 0.052 and 3 s with multiscale post-processing to segment one CBCT slice, three to four orders of magnitude faster than human experts using interactive software. IOSNet took approximately 24 s to segment a half-jaw IOS, which was approximately 50 times faster than that of human experts. The DDMF framework took 20–25 min to generate the fused 3D mesh model following the sequential processing order, which took at least 5 h for experienced human experts, even with the help of interactive software and semi-automatic algorithms, as reported in Table 6. The current inference speed is appealing to dentists. It is worth noting that in our current framework, CBCT slices were segmented one by one, followed by IOS segmentation before fusion. This inference process can be substantially accelerated by processing the CBCT slices in parallel or by segmenting the IOS and CBCT simultaneously.

DISCUSSION

CBCT segmentation

Deep convolutional neural networks (CNNs) have shown promising performance in a variety of medical image segmentation tasks, such as multiorgan, cardiac, and lesion segmentation.[25,41–44] Recently, inspired by the success of transformers in natural language processing,[45–47] transformers have been introduced into the computer vision domain to learn explicit global and long-range semantic information interactions, demonstrating superior performance on many downstream vision tasks.[31,48–50] Previous studies have also explored employing transformers for medical image analysis[3,29,30,41,42] and obtained competitive or better performance than CNNs. In this study, we proposed TSTNet, a novel approach based on the Swin Transformer backbone with additional domain-specific designs for CBCT segmentation.

Although the automatic segmentation of CBCT has been established by many groups, the automatic segmentation of the tooth and alveolar bone is a challenging task, as teeth exhibit large geometric variations, similar intensities between the tooth and alveolar

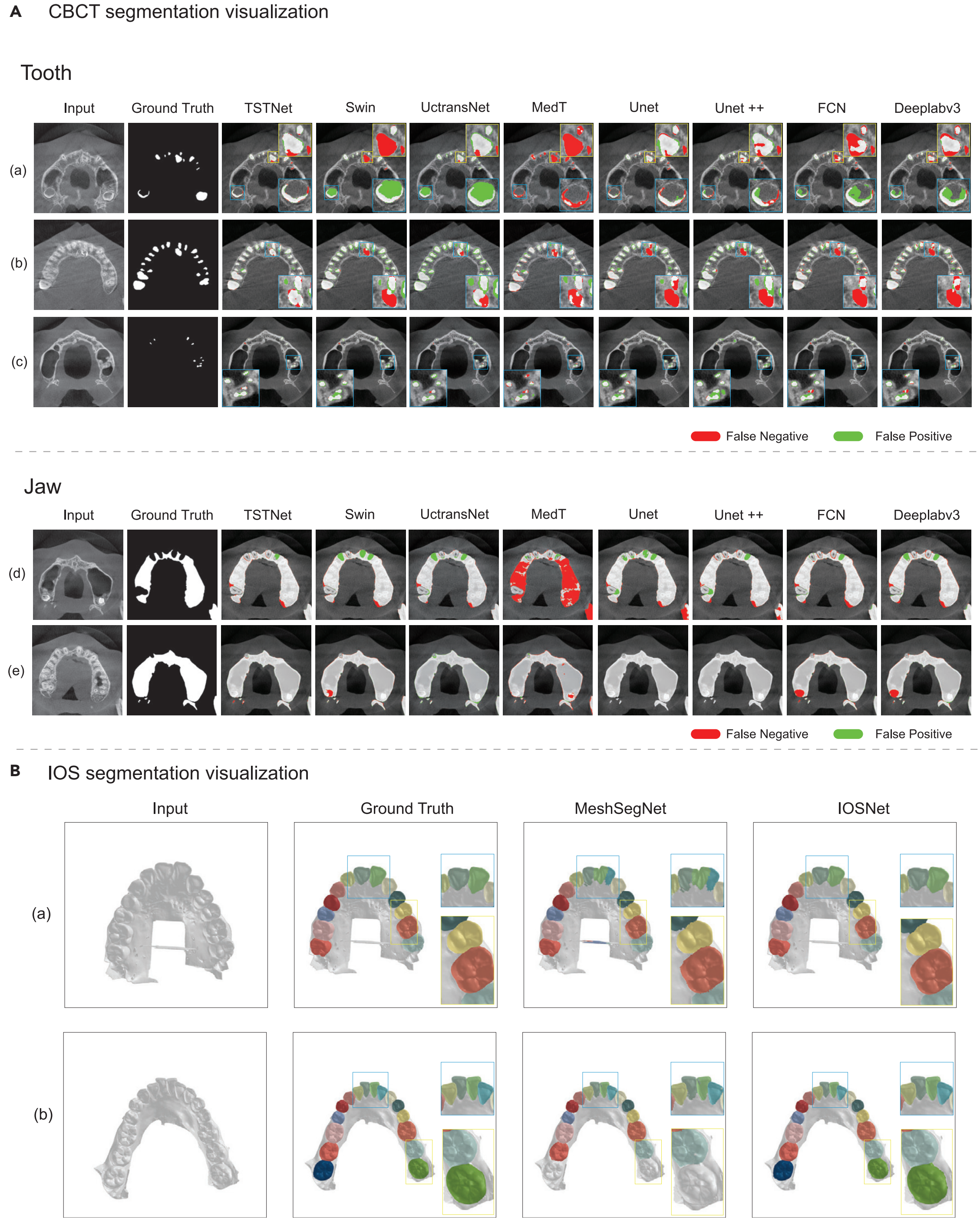

Figure 3. Visualization of the segmentation results for CBCT images and IOS meshes

(A) Five cases for tooth and alveolar bone segmentation, demonstrating that our method commits fewer mistakes with false positives and false negatives.

(B) Two cases for IOS tooth segmentation, demonstrating that our method performs much better than the state-of-the-art baselines.

bone, and complex topological and anatomical structures across different patients.[3,4,6,7,32,51] Prior works can be divided into two categories: (1) traditional methods based on hand-crafted features[51,52] and (2) deep-learning-based methods which achieve better performance than traditional methods.[3,5,7,31,52,53]

Among deep-learning-based methods, formulated tooth segmentation is an instance segmentation task on 3D CBCT images that usually requires annotating tooth instances on 3D voxels across the entire CBCT scan.[7,32] However, annotating 3D voxels in CBCT scans is costly and time-consuming, compared with

CellPress
OPEN ACCESS

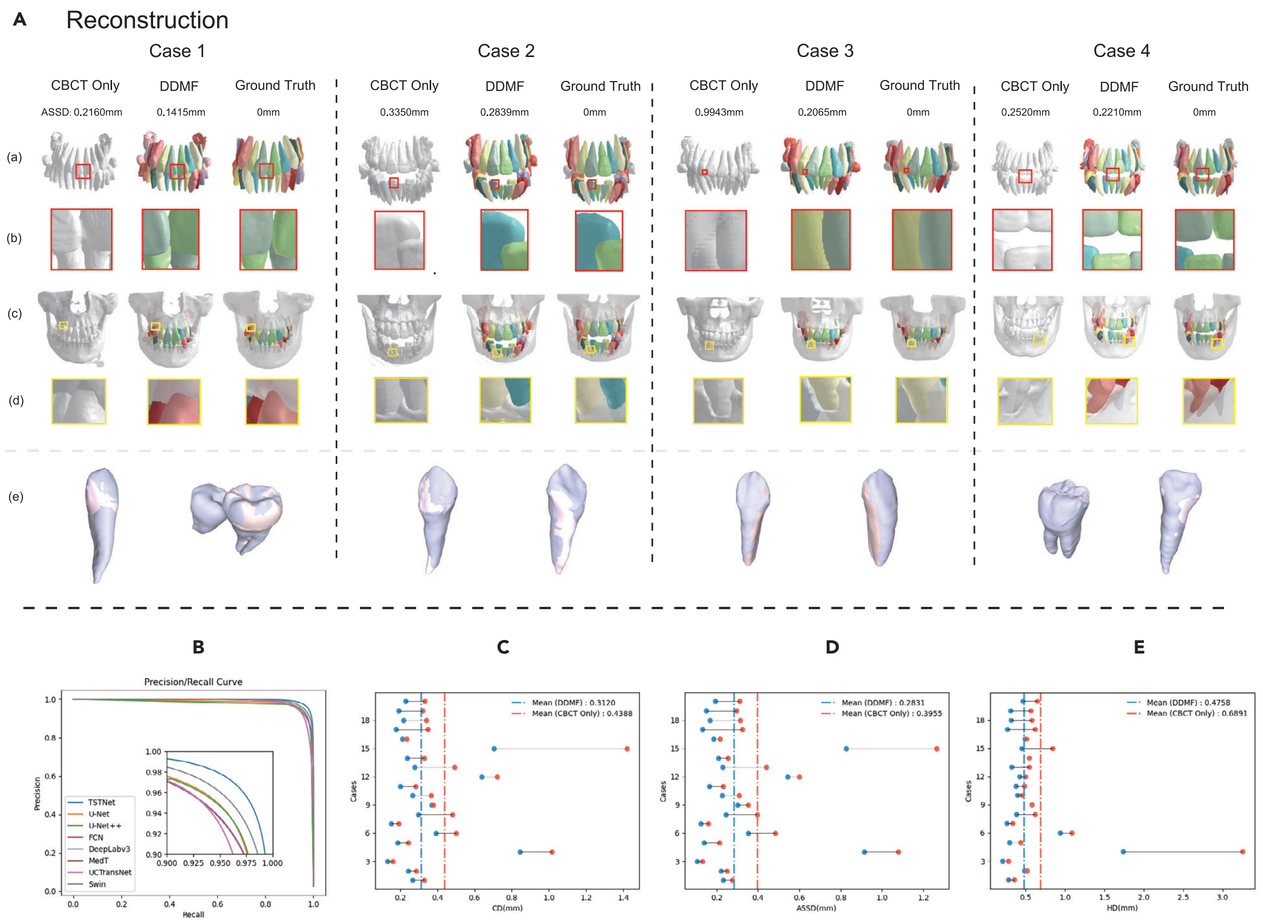

Figure 4. Visualization of the reconstruction and statistical results

(A) Reconstructed 3D CBCT meshes and the fused 3D tooth and alveolar bone of DDMF and ground truth. (Ae) shows very tiny surface deviations as indicated by the light pink colored areas, which were consistent with the small ASSD and CD errors.

(B) Statistical results of the TSTNet.

(C–E), Statistical multimodal fusion results of DDMF.

annotating some selected 2D slices.[54] Additionally, general 3D segmentation methods typically require more computational resources and exhibit a larger inference latency.[54]

Meanwhile, some 3D works only focused on tooth segmentation and ignored the alveolar bones. In contrast, we simultaneously recognized 3D teeth and alveolar bones with semantic segmentation over 2D slices. TSTNet only requires annotating tooth pixels on several 2D slices, that is, approximately 20 slices from 300–600 slices in a CBCT scan. Additionally, generating FDI tooth codes is also critical for clinical applications. Jang et al.[4] designed a tooth identification method based on 2D panoramic images reconstructed from CBCT to identify incisors, canines, premolar, and molars, then assigning FDI tooth codes accordingly. However, the two-stage classification procedure is error-prone and cannot handle complicated cases, such as missing teeth or hyperdontia because the tooth numbers are not inherently provided. Meanwhile, in the proposed DDMF framework, we identify the FDI tooth number with the help of the IOS during fusion. By doing so, we substantially reduce the human labor required for annotation as well as the network complexity and the corresponding training efforts for segmentation. For example, hierarchical or multistage networks are not needed.[4,5,7] Finally, none of the existing methods have been applied in the context of the MF of CBCT and IOS[32]; hence, their clinical applicability remains limited in practice.

IOS segmentation

IOS segmentation is closely related to 3D point cloud or mesh segmentation in computer vision, in which many methods have been proposed under different settings (e.g., PointNet and DGCNN). For 3D tooth segmentation on IOS, traditional methods use handcrafted geometrical features and semi-automatic segmentation methods.[55–60]

Recent studies have employed deep learning for automatic 3D tooth segmentation,[16–20,59,61] in which deep neural networks are used to conduct segmentation either on mesh or point clouds. The performance has been further boosted by methods that design specific neural network architectures for end-to-end tooth segmentation, such as MeshSegNet,[37,62] DCNet,[16] TSGCNet,[38] and Mask-MCNet.[63] However, most of these methods have

Table 2. Segmentation results of the IOSNet and baselines (tested on 100 patients)

Model	Mandible			Maxilla		
	mIoU, %	ACC_f, %	ACC_a, %	mIoU, %	ACC_f, %	ACC_a, %
CNN	85.32 (81.96–88.69)	91.74 (89.77–93.72)	93.75 (92.14–95.36)	89.68 (86.90–92.48)	94.14 (92.35–95.93)	95.80 (94.37–97.22)
PointNet	63.10 (57.90–69.29)	79.67 (76.32–83.01)	86.21 (83.93–88.49)	59.05 (53.14–64.95)	75.58 (71.63–79.53)	80.95 (77.67–84.24)
PointNet++	83.22 (81.38–85.06)	91.49 (90.41–92.57)	94.59 (93.91–95.27)	85.82 (84.14–87.50)	93.15 (91.92–94.38)	95.65 (94.95–96.35)
DGCNN	84.93 (83.27–86.58)	92.74 (91.82–93.65)	95.80 (95.18–96.43)	88.70 (87.55–89.86)	94.41 (93.73–95.10)	96.94 (96.42–97.45)
MeshSegNet	82.82 (80.51–85.13)	93.50 (92.55–94.45)	95.04 (94.18–95.90)	85.62 (83.61–87.64)	93.96 (93.04–94.88)	95.59 (94.77–96.41)
TSGCNet	83.04 (80.34–85.96)	92.92 (91.57–94.37)	94.60 (93.43–95.89)	80.31 (77.61–83.32)	91.53 (90.07–93.01)	93.55 (92.26–94.87)
DCNet	91.93 (91.09–92.78)	96.01 (95.42–96.61)	97.98 (97.56–98.41)	92.18 (91.02–93.36)	95.99 (95.28–96.71)	97.90 (97.33–98.45)
IOSNet	90.37 (88.69–92.06)	96.82 (96.44–97.20)	97.96 (97.61–98.31)	92.50 (91.44–93.57)	97.11 (96.79–97.44)	98.27 (97.97–98.57)
IOSNet[a]	92.44 (91.50–93.37)	97.31 (97.05–97.57)	98.41 (98.18–98.64)	93.73 (93.21–94.25)	97.36 (97.10–97.62)	98.53 (98.32–98.74)
IOSNet[b]	93.70 (92.15–95.25)*	98.14 (97.78–98.50)*	98.81 (98.49–99.14)*	95.70 (94.80–96.60)*	98.35 (98.08–98.61)*	99.05 (98.82–99.28)*

mIoU, mean intersection over union; ACC_f, per-face accuracy; ACC_a, average-area accuracy. Values in parentheses are 95% confidence intervals. All baselines and IOSNet are trained on a small dataset with 4,271 IOS scans. *Best performance.
[a]IOSNet trained on 28,000 scans without smoothing.
[b]IOSNet trained on 28,000 scans with smoothing.

been only trained and evaluated on very limited datasets, that is, datasets with 30–120 IOS scans,[16] which does not cover complex real-world cases, for example, without third molars or heterogeneous oral diseases. Hence, their applicability to heterogeneous real-world cases has not yet been verified. Recently, DCNet was the first network to be verified using a clinical applicability study and was demonstrated to generate clinically applicable results in many real-world clinical cases.[16] In comparison, our proposed IOSNet outperformed DCNet with novel loss functions on a much larger dataset. Such state-of-the-art performance corroborates its clinical applicability for segmentation in most cases.

CBCT and IOS fusion

Very few studies have been conducted on the MF of CBCT and IOS. Previous studies have relied on traditional registration and level-set segmentation methods and required considerable manual work to crop and stitch the reconstructed meshes.[23] Moreover, they required many heuristics, similar to a manual visualization system, rather than producing high-fidelity fused outputs on the basis of CBCT and IOS scans. Hence, the segmentation performance and stitched results were based only on several illustrations and were not systematically evaluated. Although some prior studies have investigated how to perform registration between CBCT and IOS scans, they did not identify the upper and lower jaws or required manual work to do so, and the instance-level tooth delineation and crown-root-bone analysis for real-world clinical use were not available as well.[4,64–66] In contrast, our proposed DDMF framework introduces novel deep-learning-based methods for CBCT and IOS segmentation and achieves end-to-end fusion with a novel half-jaw registration and fusion strategy.

One of the major contributions of our work is the demonstration of its clinical applicability in collaboration with industrial partners and hospitals and clinics in China. Our system has been implemented in real-world clinics, enabling doctors to visualize the crown-root-bone relationships during the entire orthodontic treatment process and make better treatment decisions for patients. Notably, our system's superiority in addressing the problems of root-bone relationships has been confirmed through the successful outcomes of real-world clinical cases, outperforming traditional treatment planning methods that rely solely on IOS or CBCT imaging, as illustrated in the Videos S1 and S2.

Limitations

Although our DDMF framework demonstrated superior performance and clinical applicability, several limitations must be addressed in future studies. First, the DDMF involves several intermediate steps in the fusion process, such as curvature-based segmentation, two-stage registration, and 3D fusion. This results in the most significant errors in the DDMF framework, that is, the half-jaw separation and registration method might fail in 6%–10% of total cases based on our statistics, implying that manual correction is still required for these complicated cases. Our future work will involve designing novel learning-based methods for a more accurate half-jaw separation, 3D registration, and fusion. Second, the segmentation performance for CBCT and IOS scans can be further improved. Although TSTNet and IOSNet achieved superior performance even for patients with unerupted

Table 3. Multimodal fusion results of the proposed method (tested on 1,003 teeth in 50 cases)

	Central incisor	Lateral incisor	Cuspid	1st premolar	2nd premolar	1st molar	2nd molar	Average
ASSD, mm	0.16 ± 0.06	0.17 ± 0.06	0.18 ± 0.08	0.17 ± 0.08	0.17 ± 0.07	0.19 ± 0.08	0.21 ± 0.06	0.17 ± 0.07
CD, mm	0.19 ± 0.06	0.19 ± 0.06	0.21 ± 0.07	0.20 ± 0.07	0.19 ± 0.06	0.21 ± 0.07	0.24 ± 0.06	0.20 ± 0.06
HD, mm	0.36 ± 0.15	0.36 ± 0.15	0.38 ± 0.23	0.36 ± 0.15	0.37 ± 0.16	0.41 ± 0.07	0.47 ± 0.15	0.37 ± 0.17

Data are expressed as mean ± SD. ASSD, average symmetric surface distance; CD, Chamfer distance; HD, Hausdorff distance.

Table 4. Ablation studies of TSTNet (tested on 43 patients)

DA	Loss	TEC	IoU, %	Dice, %	Recall, %	Precision, %
✓	✓	✓	93.22*	96.49*	97.75	95.27*
–	✓	✓	91.46	95.54	96.58	94.52
✓	–	✓	92.97	96.35	97.93*	94.92
✓	✓	–	92.87	96.30	97.92	94.73
–	–	–	90.52	95.02	97.69	92.50

DA, data preprocessing and augmentation. *Best performance.

teeth, hyperdontia, malposition, and ambiguous tooth boundaries, they may still be error-prone for extremely complicated cases; for example, patients with metal artifacts or root canal therapy. Future studies will combine deep learning with domain knowledge in stomatology to achieve better segmentation results for both CBCT and IOS scans. Finally, although our method has already been integrated into clinical software to assist in orthodontic treatment planning, it has been mainly evaluated from an algorithmic perspective. Therefore, the clinical usage and dental clinical findings should be further explored using rigorous multicenter clinical trials and large-scale complicated cases.

Conclusion

We proposed a novel DDMF framework of multimodal CBCT and IOS fusion for intelligent tooth crown-root-bone analyses. Our framework comprises a CBCT segmentation module, an IOS segmentation module, and a MF module. The effectiveness of each module and the entire framework was systematically demonstrated with comprehensive experiments on our large-scale multimodal dataset. Our framework had been integrated into a clinical software to assist dentists in orthodontic treatment planning and decision making. Future work includes better MF and segmentation algorithms and large-scale multicenter clinical trials.

EXPERIMENTAL PROCEDURES

Resource availability

Lead contact

The lead contact for questions about this paper is Zuozhu Liu, who can be reached at zuozhuliu@intl.zju.edu.cn.

Materials availability

This study did not generate new unique reagents.

Data and code availability

- The clinical CBCT and IOS data were collected by the hospitals in de-identified format. Owing to patient-privacy constraints, we are not able to release all dataset publicly. A partial release of the dataset is available at Zenodo under the https://doi.org/10.5281/zenodo.8027553[67] and is publicly available as of the date of publication.
- All original code has been deposited at Zenodo under the https://doi.org/10.5281/zenodo.8027716[68] and is publicly available as of the date of publication.

Table 5. Curvature-based segmentation on 3D CBCT meshes (tested on 50 patients)

	Gaussian curvature-based method	DBSCAN	This study
Success rate	42%	24%	94%

Success rate is the probability of successfully separating CBCT mesh into the maxilla and mandible.

Table 6. End-to-end model inference time and clinical utility of DDMF

Models	DDMF	Human experts
Inf-T, min	~20–25	~300–400

Inf-T, end-to-end inference time.

- Any additional information required to reanalyze the data reported in this paper is available from the lead contact upon request.

Ethics statement

The study was approved by the institutional ethics review boards of West China College of Stomatology Sichuan University (WCHSIRB-D-2021-331). The recruited patients of the retrospective and prospective parts were well informed of the study and all provided signed informed consent. Patients or the public were not involved in the design, recruitment, and conduct of the study.

Data acquisition and study design

For CBCT segmentation, each pixel is associated with an annotation for background, tooth, or alveolar bone. Note that we did not annotate all slices in 503 CBCT scans (comprising more than 150,000 slices) but only annotated 9,651 CBCT slices of them for CBCT tooth segmentation, where we selected 15 to 25 slices for each patient based on the dentists' heuristics. Most CBCT slices have a pixel resolution (height × width) ranging from 260× 260 to 1,000× 1,000. For IOS segmentation, each mesh face is associated with an annotation for the gingiva or the FDI notation of 32 different teeth. The 3D IOS mesh scans usually contain 100,000 to 400,000 triangular faces with a spatial resolution of 0.008–0.02mm. For 3D MF, each tooth is annotated by human experts assisted by CAD software to generate golden-standard 3D fused models. During annotation, a committee of experts were asked to decide whether the annotation is satisfactory for future oral diagnosis and treatment planning. The annotations were checked and improved by these experts until they met the necessary quality standards for future clinical use. Detailed annotation pipeline and data statistics are reported in the Supplementary.

Methodology

The DDMF framework includes three major steps: the CBCT segmentation, the IOS segmentation, and the MF, as illustrated in Figure 1. First, the CBCT segmentation module takes 2D CBCT slices as input and generates predictions for all pixels. Second, the IOS segmentation module takes IOS meshes as input and generates predictions for all faces in the IOS meshes. The MF module then integrates the reconstructed CBCT meshes and segmented IOS meshes to generate fused, accurate, high-resolution meshes with high-fidelity crown-root-bone structures for real-world clinical applications.

CBCT segmentation with TSTNet

We formulated the CBCT segmentation task as 2D segmentation over each CBCT slice. Suppose we have a dataset $\mathcal{D} = \{(\mathbf{x}_n, \mathbf{y}_n^t, \mathbf{y}_n^a)\}_{n=1}^N$, where $\mathbf{x}_n$, $\mathbf{y}_n^t$, and $\mathbf{y}_n^a$ denote the nth CBCT slice, corresponding pixel-level tooth and alveolar bone labels respectively, and N is the number of images in the dataset. In this step, the goal is to obtain pixel-wise tooth predictions $\widehat{\mathbf{y}}_n^t$ and alveolar bone predictions $\widehat{\mathbf{y}}_n^a$ from the CBCT slice $\mathbf{x}_n$, where the predictions can be used to reconstruct the 3D tooth and alveolar bone mesh models for subsequent usage. Below are step-by-step details of the TSTNet, as illustrated in Figure 2. More architecture details are in the Figure S1.

Data preprocessing. The number of background pixels and tooth pixels in the CBCT images $\mathbf{x}_i$ are highly imbalanced. Based on empirical statistics from the annotated masks $\mathbf{y}_n^t$, we cropped the lower 1/4 and right/top/left 1/10 in the original CBCT image $\mathbf{x}_n$ to alleviate the pixel-wise class imbalance problem. Afterward, the CBCT images $\mathbf{x}_n$ were augmented with resizing, random clip, and flip strategies.

The backbone of TSTNet. We proposed a deep-learning method based on Swin Transformer, named TSTNet, to perform 2D segmentation on each CBCT slice $\mathbf{x}_n$. As shown in Figure 2, TSTNet is composed of a backbone network and multiple segmentation heads. Swin Transformer[31] is utilized as the backbone, which is a hierarchical Transformer whose representation is computed

with Shifted windows. The hierarchical architecture has the flexibility to model at various scales, capable of extracting both local and global features, where the local features help identify the boundary between the background class and the tooth class, and the global features provide richer context information for robust classification. Such designs allow the Swin Transformer to achieve superior performance on multiple vision tasks compared with CNN based methods. Specifically, the backbone of TSTNet contains four stages. Each stage is constructed using 2/2/18/2 Swin Transformer blocks, and the multi-head attention (MHA) modules within these blocks contain 4/18/16/32 heads respectively.[31] We followed the original setting of Swin Transformer to employ the UperNet head as the main segmentation head for multiscale feature aggregation and fusion. More details about the backbone can be found in.[31]

Tooth error calibration head. Existing segmentation heads misclassified the pixels in the boundary between the tooth and alveolar bone. To alleviate this issue, we proposed the tooth error calibration (TEC) head to correct the error-prone feature representations in boundary areas for better segmentation performance. Notably, TEC can be incorporated into the hidden layers during training, and decoupled in the inference stage without additional parameters and inference time. TEC consists of the image decoupling, prototype clustering, and error calibration submodules, as illustrated in Figure 2.

The ground truth $\mathbf{y}_n^t$ and the predicted tooth mask of the UperNet head serve as inputs for TEC. The image decoupling submodule categorizes the pixels into three sets based on predictions: true positive (TP) s_k^{tp}, FN s_k^{fn}, and FP s_k^{fp} for category k, where k denotes the tooth category in our case. Based on the current and historical TP pixels, the prototype clustering submodule calculates the category prototype μ_k via exponential moving average (EMA)[69]:

$$\mu_k = \rho\mu_k + (1-\rho)\frac{1}{n_k^{tp}}\sum_{i\in s_k^{tp}}\mathbf{e}_i, \quad \text{(Equation 1)}$$

where n_k^{tp} is the number of current TP pixels for category k, $\mathbf{e}_i$ is the embedding for pixel i, $\rho = 0.9$ is the momentum value to adjust the retained proportion of historical prototype. Afterward, the cosine similarity between pixel i and prototype μ_k in embedding space is defined as follows:

$$\cos\theta_{ik} = \tilde{\mu}_k\tilde{\mathbf{e}}_i^\top = \frac{\mu_k\mathbf{e}_i^\top}{\|\mu_k\|_2\|\mathbf{e}_i\|_2}, \quad \text{(Equation 2)}$$

where $\|\cdot\|_2$ is the L2 distance and $\tilde{\mu}_k$ is the normalized vector with magnitude 1. Our method aims at maximizing $\cos\theta_{ik}$ to make $\mathbf{e}_i$ close to prototype μ_k, thus clustering pixels of the same category for better tooth segmentation performance.

The TEC simultaneously calculates the penalty terms for FN and FP pixels with an error calibration submodule. The FP errors occur when pixels of the background are excessively similar to the tooth pixels in the feature space, while the FN errors occur when pixels belong to the tooth misclassified as background. To tackle this, we take pixel i of the tooth category k as an anchor. The FN penalty term pulls the FN pixels toward the anchor of the tooth class, while the FP penalty term pushes the FP pixels to the opposite pole against the anchor in the embedding space, i.e., as background pixels. We define the FN and FP penalty terms as follows:

$$T_i^{fp} = \begin{cases} 1+\dfrac{1}{n_k^{fp}}\displaystyle\sum_{j\in s_k^{fp}}\tilde{\mathbf{e}}_j\tilde{\mathbf{e}}_i^\top & \text{, if } n_k^{fp}>0 \\ 0 & \text{, otherwise.}\end{cases}, \quad \text{(Equation 3)}$$

$$T_i^{fn} = \begin{cases} 1-\dfrac{1}{n_k^{fn}}\displaystyle\sum_{j\in s_k^{fn}}\tilde{\mathbf{e}}_j\tilde{\mathbf{e}}_i^\top & \text{, if } n_k^{fn}>0 \\ 0 & \text{, otherwise.}\end{cases}. \quad \text{(Equation 4)}$$

T_i^{fp} is 0 when there are no FP pixels or the average similarity converges to -1, indicating all FP pixels are in the opposite direction from anchor i. T_i^{fn} is reduced to 0 when there are no FN pixels or all FN and TP pixels are placed in the same direction in the feature space. Finally, TEC integrates the cosine similarities and the FP/FN penalty terms into a TEC loss, serving as an additional loss to train the network:

$$L_i^{\text{TEC}} = -\log\frac{\exp\left(\cos\theta_{ik}/\tau-(1-p_{ik})T_i^{fn}\right)}{\exp\left(\cos\theta_{ik}/\tau-(1-p_{ik})T_i^{fn}\right)+\sum_{l\neq k}\exp(\cos\theta_{il}/\tau)} - \log\frac{\exp\left(\cos\theta_{ik}/\tau-(1-p_{ik})T_i^{fp}\right)}{\exp\left(\cos\theta_{ik}/\tau-(1-p_{ik})T_i^{fp}\right)+\sum_{l\neq k}\exp(\cos\theta_{il}/\tau)}, \quad \text{(Equation 5)}$$

where $\tau = 0.5$ is the temperature, and p_{ik} is the probability prediction for wrongly segmented pixels. By minimizing L_i^{TEC}, all FP pixels are pushed to the opposite direction against anchor i, and all FN pixels are pulled to the same direction toward anchor i.

Loss function of TSTNet. In CBCT slices, the area of the background class is roughly ten times larger than that of the tooth class, which leads to a severe class imbalance problem. To overcome this issue, the class balanced cross entropy loss and online hard example mining (OHEM) cross entropy loss[33,70] are introduced as the objective function of the UperNet head, intending to avoid overfitting of the background class. The OHEM loss tends to choose the examples with higher loss or more diversity as the training data and assign different weights to different classes, reducing the bias to the class with majority samples.[71] Besides the UperNet and TEC heads, TSTNet also employs an FCN head, serving as the auxiliary head to further improve the segmentation performance. As for the FCN head, the Lovasz-Softmax loss is employed as the objective function, which performs better on segmenting small objects and reducing FNs, and helps alleviate the class imbalance problem to some extent and avoid the missed detection of tooth area.[70,72] Together with the OHEM cross-entropy loss and the pixel-level TEC representation learning modules with a novel TEC loss, TSTNet is able to attain fine-grained segmentation results across various tooth anatomies. Overall, the total loss for TSTNet on pixel i is defined as:

$$L_i^{\text{Seg}} = L_i^{\text{OHEM}} + L_i^{\text{Lovasz}} + \lambda L i^{\text{TEC}}, \quad \text{(Equation 6)}$$

where $\lambda = 0.1$ is the weight of the TEC loss. During inference, the TEC and FCN heads are discarded.

IOS segmentation with IOSNet

We performed 3D segmentation on IOS meshes. The goal of this step is to predict the label $\mathbf{y}_n^l$ given any face f_n in the mesh $\mathcal{M}_l$, where $\mathbf{y}_n^l \in \{0, 11-18, 21-28, 31-38, 41-48\}$ denotes the gingiva and FDI notations for the 32 permanent teeth, respectively. We first transformed the IOS mesh to a point cloud during preprocessing by randomly sampling 10,000 face center points from each IOS mesh. For each point, we extracted the 3D coordinates, face normal vector, and face shape descriptor to form a 15-dimensional feature vector, as in.[16] Then, the point clouds were segmented with the IOSNet and further mapped back to the original meshes with a k-nearest neighbor aggregation strategy, followed by a standard smoothing based on graph-cut for post-processing.[16,17] The architecture of the IOSNet is illustrated in Figure 2, where we adopted the Edge-Conv block with similar architectures as in.[16,36] More architecture details are in the Figure S2.

Novel loss functions in IOSNet. Though several methods have been proposed for tooth segmentation in IOS, they had limitations in precise boundary segmentation or generalization to complicated anatomies, such as crowded teeth and hyperdontia. To alleviate this issue, we proposed two novel loss functions. The first one is a centroid loss which helps learn the coarse tooth shapes, avoiding weird segmentations (recognizing two teeth as one or vice versa). The second one is boundary loss which helps produce accurate boundary predictions for complicated samples, i.e., mesh faces between tooth-tooth or tooth-gingiva boundaries.

In IOS mesh, adjacent teeth were frequently misidentified as a single tooth because of their similar structures or close connections. To achieve accurate segmentation, we designed the centroid loss to assure that the geometrical center of each tooth can be correctly captured. Specifically, given the tooth point cloud P, the centroid loss is defined as:

$$L^{\text{centroid}} = \frac{1}{C}\sum_{i=1}^{C} dis(pc_i - gc_i), \quad \text{(Equation 7)}$$

where $C = 33$ denotes the number of categories in the annotations, $dis(\cdot)$ denotes the Euclidean distance of two points, pc_i and gc_i represent the prediction centroid and the gold centroid for the ith class, respectively, which are defined as:

$$pc_i = \frac{\sum_{j=1}^{N_i} \tilde{p}_j \times s_j}{\sum_{j=1}^{N_i} \tilde{p}_j}, \quad \text{(Equation 8)}$$

$$gc_i = \frac{\sum_{j=1}^{M_i} s_j}{M_i}, \quad \text{(Equation 9)}$$

where $\tilde{p}_j$ is defined as:

$$\tilde{p}_j = \begin{cases} 1, & p_j > th_u \\ 0, & p_j < th_l \\ p_j, & \text{otherwise} \end{cases}, \quad \text{(Equation 10)}$$

and N_i and M_i denote the number of points in the ith class except the gingiva in the prediction and the ground truth, p_j denotes the probability of the jth point predicted as the ith class, and s_j represents the 3D coordinates of the jth point. Here, we empirically defined th_l and th_u as 0.38 and 0.6, respectively, to eliminate points with low confidence during computing the centroids. By minimizing L^{centroid}, we encouraged the predictions to maintain the same geometrical centers as the ground truth, avoiding weird segmentation results.

Another challenge in 3D tooth segmentation is to accurately delineate the tooth-tooth or tooth-gingiva boundaries. To cope with the issue, we designed a novel boundary loss, which is defined as:

$$L^{\text{boundary}} = L^{ce}(pb, gb), \quad \text{(Equation 11)}$$

where L^{ce} denotes the cross entropy loss, pb and gb represent the predicted probability and ground truth label of the boundary points, respectively. Here, the top 5% points were chosen as the boundary points according to the KL-divergence of all the points, while the KL-divergence for the ith point is defined as:

$$KL\_div_i = \max_{j \in \kappa_i} KLD(c_i, k_j), \quad \text{(Equation 12)}$$

where c_i denotes the prediction probability distribution of the center point i, and k_j denotes the jth neighbor point's probability distribution, KLD is the KL-divergence between two distributions. We used maximum as the aggregation operation in a local neighborhood to select the most representative value from a set of KLD values computed by probability distribution of the center point and its neighbor points. KL_div_i indicates the distributional difference between the ith point and its $K = |\kappa_i|$ surrounding points, while points with a large KL_div_i indicate it is of high probability to become a boundary point. We set $K = 5$ in our implementation. Overall, the total loss for IOSNet is defined as:

$$L^{\text{total}} = L^{\text{ce}}(p, g) + L^{\text{centroid}} + L^{\text{boundary}}, \quad \text{(Equation 13)}$$

where L^{ce} denotes the cross entropy loss, p and g denote the prediction scores of all points from IOSNet and the ground truth of all points respectively.

Experimental setup

For TSTNet, the CBCT images were resized to random resolution within $2,048\times 2,048$, and subsequently randomly clipped to 512×512 and flipped. We employed the AdamW optimizer with an initial learning rate of $6e^{-5}$ and a weight decay of 0.01, a scheduler that used linear learning rate decay, and a linear warmup of 1,500 iterations. All models were trained on NVIDIA RTX 3090 GPUs with batch size 16 for 160,000 iterations. For IOSNet, we followed the procedures in Hao et al.[16] and Xu et al.[17] to adopt a graph-cut-based boundary smoothing for post-processing, training all models with 10,000 points. We employed AdamW as the optimizer with an initial learning rate of $3e^{-3}$ and a weight decay of $1e^{-3}$. All models were trained on NVIDIA RTX 3090 GPUs with batch size 8 for 200 epochs.

Multimodal fusion of CBCT and IOS

With the segmentation outputs from the previous steps, the MF module aims to produce fused 3D tooth instances with high-fidelity toot crowns from IOS, comprehensive tooth roots from CBCT, and accurate alveolar bones from CBCT, leading to complete 3D dental models for accurate tooth crown-root-bone analysis in clinical applications.

The MF modules include three steps: 1) 3D CBCT mesh reconstruction and automatic segmentation of half jaws and individual teeth in the reconstructed mesh based on point curvature feature; 2) Registration between CBCT data and IOS tooth crown meshes (mandible and maxilla); 3) Replacement of the crowns in CBCT meshes with IOS meshes to obtain fused clinically applicable dental models, as illustrated in Figure 1 - Step 3: Multimodal Data Fusion.

Point curvature feature-based half-jaw segmentation. The MF module first reconstructs the 3D meshes for the teeth and alveolar bones from CBCT based on the standard marching-cube reconstruction and HLO smoothing algorithms.[73] The CBCT and IOS usually have different bite positions. The CBCT usually includes both upper and lower jaws with close or open bite positions as in Figure 5A, while the IOS usually includes separated half jaws. Hence, to accurately fuse both modalities, a promising method is to first get the upper and lower jaws in CBCT, and then perform half-jaw registration and fusion between CBCT and IOS. However, as shown in Figure 5A, there usually exist connected boundaries between adjacent teeth or the contacts in maxilla and mandible, especially for patients in close bite positions, which imposes significant difficulties for accurate half jaw registration and individual teeth delineation. To address this issue, we proposed a geometry-based segmentation algorithm to separate the half jaws and further delineate each tooth in CBCT meshes with a novel point curvature feature.

Specifically, the point curvature feature of a vertex is defined as the average of angles between normal vectors of all its neighbors, which is different from either mean curvature or Gaussian curvature. Mathematically, given an vertex v with normal vector n_v, we define its first-order neighbors as $u \in N(v)$, associated with normal vectors n_u, the point curvature feature cur_v of vertex v is defined as:

$$cur_v = \frac{1}{|N(v)|} \sum_{u \in N(v)} \arccos\left(\frac{n_v \cdot n_u}{|n_v||n_u|}\right). \quad \text{(Equation 14)}$$

This definition can be extended to lth-order neighbors by changing the set of neighbors. The intuition behind the curvature-based segmentation algorithm is that the angle between the normal vectors of adjacent points can capture the curvature of different scales by changing the level of neighbors. We experimentally found that this curvature could help recognize the ambiguous boundaries between adjacent teeth, and the upper and lower jaw contacts, while the traditional curvatures such as Gaussian curvature usually failed in such scenarios, as shown in Figure 5B and demonstrated in the following experiments.

We applied a point curvature feature-based region growth segmentation algorithm, an "erosion-expansion" procedure, to separate the upper and lower jaws, as shown in Figure 5C. The vertices with high curvature values can serve as boundaries even with connected teeth in maxilla and mandible. We first computed the point curvature feature for each vertex, and removed vertices with curvatures in the top T percent from the mesh (erosion). Followed by a simple connected component analysis algorithm, we separated the individual teeth, and merged back the deleted vertices to the nearest separated components, on top of which the KD-trees are constructed, to get unbroken reconstructed teeth (expansion). Based on individual teeth, we generated the half jaws by computing the gravity center of each tooth and separating them into upper and lower parts with a RANSAC algorithm.[74] Additional technical algorithm details were attached in the Data S1, S2 and S3.[75]

Registration of two modalities. The CBCT segmentation results from the previous section are maxillary and mandibular parts, which are to be registered with the IOS meshes. We denote the vertex set of the IOS mesh as $\mathcal{V}_I$, and the vertex set of the CBCT mesh as $\mathcal{V}_C$. The registration method is to find the optimal transformation $\mathcal{T}^* = [R|t]$ such that the transformed point cloud $\mathcal{T}^*(\text{x})$ best aligns with the target, in which $R \in SO(3)$ (3D rotation group in geometry) denotes the rotation, $t \in \mathbf{R}^3$ denotes the translation. The registration formula is defined as follows:

$$\mathcal{T}^* = \underset{\mathcal{T}}{\text{argmin}} \sum_{(v_I, v_C) \in Corr} \|v_C - \mathcal{T}(v_I)\|^2, \quad \text{(Equation 15)}$$

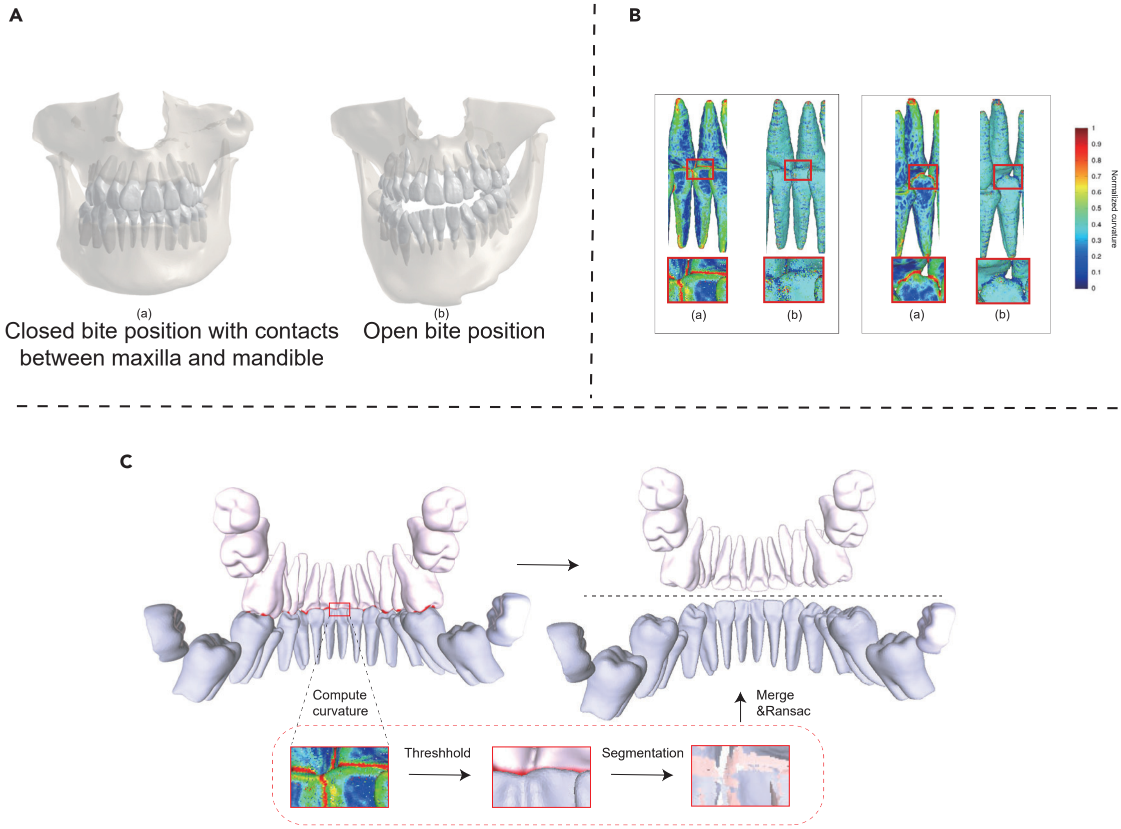

Figure 5. The details of the point curvature-based half-jaw segment

(A) The reconstructed 3D CBCT meshes. (Aa) Closed bite position with contacts between maxilla and mandible. (Ab) Open bite position.

(B) The visualization of colorized curvatures. (Ba) Our point curvature. (Bb) Gaussian curvature.

(C) The half-jaw segmentation processing based on point curvature.

where $\mathcal{T}$ is the set of rigid transformations represented by a 4× 4 matrix. $\mathcal{T}^*$ is the optimal transformation. *Corr* is a set of correspondences between $\mathcal{V}_I$ and $\mathcal{V}_C$, where $v_c \in \mathcal{V}_C, v_i \in \mathcal{V}_I$.

We proposed a two-stage registration method composed of a global registration to roughly register the two point sets as the initial alignment, and an improved ICP registration step for further refinement. In the global registration, the CBCT and IOS meshes are first down-sampled with voxel size of 0.6 and then registered with a RANSAC scheme on the 33-dimensional FPFH vector space.[76] The segmentation is further improved by the multiscale ICP algorithm performing point-to-plane ICP registration at three scales. In particular, we performed multiscale ICP with point-to-plane ICP registration[77] at three sequential scales (with distance thresholds of 0.36, 0.18, 0.12)[78]:

$$\mathcal{T}^i = \underset{\mathcal{T}}{\text{argmin}} \sum_{(v_i, v_c) \in Corr^i} \left\| v_c - \mathcal{T}^{i-1}(v_i) \right\|^2, i = 1, 2, 3, \quad \text{(Equation 16)}$$

where $\mathcal{T}^0$ is the global rigid transformation obtained at the distance threshold of 0.36, and $\mathcal{T}^1, \mathcal{T}^2$ denote the corresponding iterative multiscale registration with distance thresholds 0.18 and 0.12, respectively. Finally, we took the transformed mesh by $\mathcal{T}^2$ as the registration output.

Tooth crown replacement with mesh fusion. The final step in this MF module is to replace the low-resolution and error-prone CBCT crown with the high-fidelity and clinically applicable IOS crown. We performed the fusion on the point level, taking the registration results as the inputs. Specifically, the points $\mathcal{V}_I$ in the IOS mesh $\mathcal{M}_I$ were taken as reference, and the points belonging to the CBCT crown, i.e., $\mathcal{V}_C$ in $\mathcal{M}_C$, were removed. These points were identified based on the Euclidean distance of the points in $\mathcal{V}_C$ to the KDTree constructed from IOS point clouds $\mathcal{V}_I$. With an empirical threshold value on the Euclidean distance, followed by a simple DBSCAN algorithm[40] to detect the isolated outlier points, we were able to easily identify, crop, and replace the tooth crown points in $\mathcal{V}_C$ with points in $\mathcal{V}_I$. Finally, we got the fused mesh *P* based on the fused point cloud and the original normal vectors from the IOS and CBCT mesh $(\mathcal{M}_I, \mathcal{M}_C)$ with Poisson reconstruction. More technical details regarding the tooth crown replacement steps are attached in the Data S4.[75]

SUPPLEMENTAL INFORMATION

Supplemental information can be found online at https://doi.org/10.1016/j.patter.2023.100825.

ACKNOWLEDGMENTS

This work is supported by the National Natural Science Foundation of China (grant 62106222), the Natural Science Foundation of Zhejiang Province (grant LZ23F020008), Sichuan Science and Technology Program (2022ZDZX0031), and the Zhejiang University-Angelalign Inc. R&D Center for Intelligent Healthcare.

AUTHOR CONTRIBUTIONS

J.H., Z.L., and Z.Z. initiated this project. J. Liu, J.H., H.L., W.P., and J.Y. designed and implemented the model architecture. J. Liu, W.P., and Z.L. performed validation experiments. J. Liu, Z.L., J. Li, Z.J., and Y.F. created the dataset. J.H., Y.F., Z.J., and G.W. performed clinical validation experiments. J.H., J. Liu, and Z.L. wrote the manuscript. J.H., Z.L., and Z.Z. supervised this project. All authors read and contributed to revision and approved the manuscript.

CellPress
OPEN ACCESS

DECLARATION OF INTERESTS

Y.F. is employed with Angelalign Inc. W.P. is employed with OPT Machine Vision Tech Co., Ltd. Japan.

Received: December 15, 2022
Revised: March 24, 2023
Accepted: July 21, 2023
Published: August 15, 2023

REFERENCES

1. Weiss, R., and Read-Fuller, A. (2019). Cone beam computed tomography in oral and maxillofacial surgery: an evidence-based review. Dent. J. *7*, 52.
2. Liebregts, J., Xi, T., Timmermans, M., de Koning, M., Bergé, S., Hoppenreijs, T., and Maal, T. (2015). Accuracy of three-dimensional soft tissue simulation in bimaxillary osteotomies. J. Cranio-Maxillo-Fac. Surg. *43*, 329–335.
3. Wang, H., Minnema, J., Batenburg, K.J., Forouzanfar, T., Hu, F.J., and Wu, G. (2021). Multiclass cbct image segmentation for orthodontics with deep learning. J. Dent. Res. *100*, 943–949.
4. Jang, T.J., Kim, K.C., Cho, H.C., and Seo, J.K. (2022). A fully automated method for 3d individual tooth identification and segmentation in dental cbct. IEEE Trans. Pattern Anal. Mach. Intell. *44*, 6562–6568.
5. Shaheen, E., Leite, A., Alqahtani, K.A., Smolders, A., Van Gerven, A., Willems, H., and Jacobs, R. (2021). A novel deep learning system for multi-class tooth segmentation and classification on cone beam computed tomography. a validation study. J. Dent. *115*, 103865.
6. Huang, J., Yan, H., Li, J., Stewart, H.M., and Setzer, F. (2021). Combining anatomical constraints and deep learning for 3-d cbct dental image multi-label segmentation. In 2021 IEEE 37th International Conference on Data Engineering (ICDE) (IEEE), pp. 2750–2755.
7. Cui, Z., Li, C., and Wang, W.T. (2019). ToothNet: automatic tooth instance segmentation and identification from cone beam ct images. In Proceedings of the IEEE/CVF Conference on Computer Vision and Pattern Recognition, pp. 6368–6377.
8. Komuro, A., Yamada, Y., Uesugi, S., Terashima, H., Kimura, M., Kishimoto, H., Iida, T., Sakamoto, K., Okuda, K., Kusano, K., et al. (2021). Accuracy and dimensional reproducibility by model scanning, intraoral scanning, and cbct imaging for digital implant dentistry. Int. J. Implant Dent. *7*, 63–67.
9. Patcas, R., Müller, L., Ullrich, O., and Peltomäki, T. (2012). Accuracy of cone-beam computed tomography at different resolutions assessed on the bony covering of the mandibular anterior teeth. Am. J. Orthod. Dentofacial Orthop. *141*, 41–50.
10. Savoldelli, C., Vandersteen, C., Dassonville, O., and Santini, J. (2018). Dental occlusal-surface-supported titanium guide to assist cutting and drilling in mandibular bilateral sagittal split osteotomy. J. Stomatol. Oral Maxillofac. Surg. *119*, 75–78.
11. Flügge, T.V., Nelson, K., Schmelzeisen, R., and Metzger, M.C. (2013). Three-dimensional plotting and printing of an implant drilling guide: simplifying guided implant surgery. J. Oral Maxillofac. Surg. *71*, 1340–1346.
12. Polley, J.W., and Figueroa, A.A. (2013). Orthognathic positioning system: intraoperative system to transfer virtual surgical plan to operating field during orthognathic surgery. J. Oral Maxillofac. Surg. *71*, 911–920.
13. Schulze, R., Heil, U., Gross, D., Bruellmann, D.D., Dranischnikow, E., Schwanecke, U., and Schoemer, E. (2011). Artefacts in CBCT: A review. Dentomaxillofacial Radiol. *40*, 265–273.
14. Hirschinger, V., Hanke, S., Hirschfelder, U., and Hofmann, E. (2015). Artifacts in orthodontic bracket systems in cone-beam computed tomography and multislice computed tomography. J. Orofac. Orthop. *76*, 152–160.
15. Schulze, R.K.W., Berndt, D., and d'Hoedt, B. (2010). On cone-beam computed tomography artifacts induced by titanium implants. Clin. Oral Implants Res. *21*, 100–107.
16. Hao, J., Liao, W., Zhang, Y.L., Peng, J., Zhao, Z., Chen, Z., Zhou, B.W., Feng, Y., Fang, B., Liu, Z.Z., and Zhao, Z.H. (2022). Toward clinically applicable 3-dimensional tooth segmentation via deep learning. J. Dent. Res. *101*, 304–311.
17. Xu, X., Liu, C., and Zheng, Y. (2019). 3d tooth segmentation and labeling using deep convolutional neural networks. IEEE Trans. Vis. Comput. Graph. *25*, 2336–2348.
18. Tian, S., Dai, N., Zhang, B., Yuan, F., Yu, Q., and Cheng, X. (2019). Automatic classification and segmentation of teeth on 3d dental model using hierarchical deep learning networks. IEEE Access *7*, 84817–84828.
19. Zanjani, F.G., Moin, D.A., Verheij, B., Claessen, F., Cherici, T., Tan, T., and de With, P.H.N. (2019). Deep learning approach to semantic segmentation in 3d point cloud intra-oral scans of teeth. In International Conference on Medical Imaging with Deep Learning (PMLR), pp. 557–571.
20. Cui, Z., Li, C., Chen, N., Wei, G., Chen, R., Zhou, Y., Shen, D., and Wang, W. (2021). Tsegnet: an efficient and accurate tooth segmentation network on 3d dental model. Med. Image Anal. *69*, 101949.
21. Mangano, F., Gandolfi, A., Luongo, G., and Logozzo, S. (2017). Intraoral scanners in dentistry: a review of the current literature. BMC Oral Health *17*, 149–211.
22. Jang, T.J., Yun, H.S., Kim, J.-E., Lee, S.-H., and Seo, J.K. (2021). Fully automatic integration of dental cbct images and full-arch intraoral impressions with stitching error correction via individual tooth segmentation and identification. Preprint at arXiv. https://doi.org/10.48550/arXiv.2112.01784.
23. Qian, J., Lu, S., Gao, Y., Tao, Y., Lin, J., and Lin, H. (2021). An automatic tooth reconstruction method based on multimodal data. J. Vis. *24*, 205–221.
24. Cruz, R.S., Lebrat, L., Bourgeat, P., Fookes, C., Fripp, J., and Salvado, O.D. (2021). A 3d deep learning approach for cortical surface reconstruction. In Proceedings of the IEEE/CVF Winter Conference on Applications of Computer Vision, pp. 806–815.
25. Ronneberger, O., Fischer, P., and Brox, T. U-net (2015). Convolutional networks for biomedical image segmentation. In International Conference on Medical image computing and computer-assisted intervention (Springer), pp. 234–241.
26. Zhou, Z., Siddiquee, M.M.R., Tajbakhsh, N., and Liang, J. (2020). Unet++: Redesigning skip connections to exploit multiscale features in image segmentation. IEEE Trans. Med. Imag. *39*, 1856–1867.
27. Chen, L.-C., Papandreou, G., Schroff, F., and Adam, H. (2017). Rethinking atrous convolution for semantic image segmentation. Preprint at arXiv. https://doi.org/10.48550/arXiv.1706.05587.
28. Long, J., Shelhamer, E., and Darrell, T. (2015). Fully convolutional networks for semantic segmentation. In Proceedings of the IEEE conference on computer vision and pattern recognition, pp. 3431–3440.
29. Valanarasu, J.M.J., Oza, P., Hacihaliloglu, I., and Patel, V.M. (2021). Medical transformer: Gated axial-attention for medical image segmentation. In International Conference on Medical Image Computing and Computer-Assisted Intervention (Springer), pp. 36–46.
30. Wang, H.W., Cao, P., Wang, J., and Zaiane, O.R. (2022). Uctransnet: Rethinking the skip connections in u-net from a channel-wise perspective with transformer. Proc. AAAI Conf. Artif. Intell. *61*, 2441–2442.
31. Liu, Z., Lin, Y., Cao, Y., Hu, H., Wei, Y., Zhang, Z., Lin, S., and Guo, B. (2021). Swin transformer: Hierarchical vision transformer using shifted windows. In Proceedings of the IEEE/CVF International Conference on Computer Vision, pp. 10012–10022.
32. Cui, Z., Fang, Y., Mei, L., Zhang, B., Yu, B., Liu, J., Jiang, C., Sun, Y., Ma, L., Huang, J., et al. (2022). A fully automatic ai system for tooth and alveolar bone segmentation from cone-beam ct images. Nat. Commun. *13*, 2096–2111.
33. Xu, J., Chen, K., and Lin, D. (2020). Mmsegmenation. https://github.com/open-mmlab/mmsegmentation.
34. Qi, C.R., Su, H., Mo, K., and Guibas, L.J.P. (2017). Deep learning on point sets for 3d classification and segmentation. In Proceedings of the IEEE conference on computer vision and pattern recognition, pp. 652–660.

35. Qi, C.R., Yi, L., Su, H., and Guibas, L.J. (2017). Pointnet++: Deep hierarchical feature learning on point sets in a metric space. Adv. Neural Inf. Process. Syst. *30*.

36. Wang, Y., Sun, Y., Liu, Z., Sarma, S.E., Bronstein, M.M., and Solomon, J.M. (2019). Dynamic graph cnn for learning on point clouds. ACM Trans. Graph. *38*, 1–12.

37. Lian, C., Wang, L., Wu, T.-H., Wang, F., Yap, P.-T., Ko, C.-C., and Shen, D. (2020). Deep multi-scale mesh feature learning for automated labeling of raw dental surfaces from 3d intraoral scanners. IEEE Trans. Med. Imag. *39*, 2440–2450.

38. Zhang, L., Zhao, Y., Meng, D., Cui, Z., Gao, C., Gao, X., Lian, C., and Shen, D.T. (2021). Discriminative geometric feature learning with two-stream graph convolutional network for 3d dental model segmentation. In Proceedings of the IEEE/CVF Conference on Computer Vision and Pattern Recognition, pp. 6699–6708.

39. Fan, H., Su, H., and Guibas, L.J. (2017). A point set generation network for 3d object reconstruction from a single image. In Proceedings of the IEEE conference on computer vision and pattern recognition, pp. 605–613.

40. Ester, M., Kriegel, H.-P., Sander, J., and Xu, X. (1996). A density-based algorithm for discovering clusters in large spatial databases with noise. In Proc. of The 2nd Int'l Conf. on Knowledge Discovery and Data Mining, pp. 226–231.

41. Hatamizadeh, A., Tang, Y., Nath, V., Yang, D., Myronenko, A., Landman, B., Roth, H.R., and Xu, D. (2022). UNETR: Transformers for 3d medical image segmentation. In Proceedings of the IEEE/CVF Winter Conference on Applications of Computer Vision, pp. 574–584.

42. Hatamizadeh, A., Nath, V., Tang, Y., Yang, D., Roth, H.R., and Xu, D. (2022). Swin unetr: Swin transformers for semantic segmentation of brain tumors in mri images. In Brainlesion: Glioma, Multiple Sclerosis, Stroke and Traumatic Brain Injuries: 7th International Workshop, BrainLes 2021, Held in Conjunction with MICCAI 2021, Virtual Event, September 27, 2021, Revised Selected Papers, Part I (Springer), pp. 272–284.

43. Isensee, F., Jaeger, P.F., Kohl, S.A.A., Petersen, J., and Maier-Hein, K.H. (2021). nnu-net: a self-configuring method for deep learning-based biomedical image segmentation. Nat. Methods *18*, 203–211.

44. Milletari, F., Navab, N., and Ahmadi, S.-A. (2016). V-net: Fully convolutional neural networks for volumetric medical image segmentation. In 2016 fourth international conference on 3D vision (3DV) (IEEE), pp. 565–571.

45. Vaswani, A., Shazeer, N., Parmar, N., Uszkoreit, J., Jones, L., Gomez, A.N., Kaiser, Ł., and Polosukhin, I. (2017). Attention is all you need. Adv. Neural Inf. Process. Syst. *30*.

46. Kenton, J.D., Chang, M.-W., Lee, K., and Toutanova, K. (2019). Pretraining of deep bidirectional transformers for language understanding. In Proceedings of NAACL-HLT, pp. 4171–4186.

47. Radford, A., Narasimhan, K., Salimans, T., and Sutskever, I. (2018). Improving Language Understanding by Generative Pre-training. https://www.cs.ubc.ca/~amuham01/LING530/papers/radford2018improving.pdf.

48. Carion, N., Massa, F., Synnaeve, G., Usunier, N., Kirillov, A., and Zagoruyko, S. (2020). End-to-end object detection with transformers. In European conference on computer vision (Springer), pp. 213–229.

49. Guo, M.-H., Xu, T.-X., Liu, J.-J., Liu, Z.-N., Jiang, P.-T., Mu, T.-J., Zhang, S.-H., Martin, R.R., Cheng, M.-M., and Hu, S.-M. (2022). Attention Mechanisms in Computer Vision: A Survey (Computational Visual Media), pp. 1–38.

50. Han, K., Wang, Y., Chen, H., Chen, X., Guo, J., Liu, Z., Tang, Y., Xiao, A., Xu, C., Xu, Y., et al. (2020). A survey on visual transformer. Preprint at arXiv. https://doi.org/10.1109/TPAMI.2022.3152247.

51. Gan, Y., Xia, Z., Xiong, J., Li, G., and Zhao, Q. (2018). Tooth and alveolar bone segmentation from dental computed tomography images. IEEE J. Biomed. Health Inform. *22*, 196–204.

52. Wang, L., Gao, Y., Shi, F., Li, G., Chen, K.-C., Tang, Z., Xia, J.J., and Shen, D. (2016). Automated segmentation of dental cbct image with prior-guided sequential random forests. Med. Phys. *43*, 336–346.

53. Ezhov, M., Zakirov, A., and Gusarev, M. (2019). Coarse-to-fine volumetric segmentation of teeth in cone-beam ct. In 2019 IEEE 16th International Symposium on Biomedical Imaging (ISBI 2019) (IEEE), pp. 52–56.

54. Xie, Y., Zhang, J., Xia, Y., and Wu, Q. (2022). UniMiss: Universal medical self-supervised learning via breaking dimensionality barrier. In Computer Vision–ECCV 2022: 17th European Conference, Tel Aviv, Israel, October 23–27, 2022, Proceedings, Part XXI (Springer), pp. 558–575.

55. Zou, B.-j., Liu, S.-j., Liao, S.-h., Ding, X., and Liang, Y. (2015). Interactive tooth partition of dental mesh base on tooth-target harmonic field. Comput. Biol. Med. *56*, 132–144.

56. Kondo, T., Ong, S.H., and Foong, K.W.C. (2004). Tooth segmentation of dental study models using range images. IEEE Trans. Med. Imag. *23*, 350–362.

57. Kumar, Y., Janardan, R., Larson, B., and Moon, J. (2011). Improved segmentation of teeth in dental models. Computer-Aided Design and Applications *8*, 211–224.

58. Fan, R., Jin, X., and Wang, C.C.L. (2015). Multiregion segmentation based on compact shape prior. IEEE Trans. Autom. Sci. Eng. *12*, 1047–1058.

59. Li, Z., and Wang, H. (2016). Interactive tooth separation from dental model using segmentation field. PLoS One *11*, e0161159.

60. Qiu, L., Ye, C., Chen, P., Liu, Y., Han, X., and Cui, S. (2022). Darch: Dental arch prior-assisted 3d tooth instance segmentation with weak annotations. In Proceedings of the IEEE/CVF Conference on Computer Vision and Pattern Recognition, pp. 20752–20761.

61. Wu, T.-H., Lian, C., Lee, S., Pastewait, M., Piers, C., Liu, J., Wang, F., Wang, L., Chiu, C.-Y., Wang, W., et al. (2022). Two-stage mesh deep learning for automated tooth segmentation and landmark localization on 3d intraoral scans. IEEE Trans. Med. Imag. *41*, 3158–3166.

62. Hu, H., Li, Z., and Gao, W. (2023). Mpcnet: Improved meshsegnet based on position encoding and channel attention. IEEE Access *11*, 23326–23334.

63. Zanjani, F.G., Moin, D.A., Claessen, F., Cherici, T., Parinussa, S., Pourtaherian, A., Zinger, S., and de With, P.H.N. (2019). Mask-mcnet: Instance segmentation in 3d point cloud of intra-oral scans. In International Conference on Medical Image Computing and Computer-Assisted Intervention (Springer), pp. 128–136.

64. Gateno, J., Xia, J., Teichgraeber, J.F., and Rosen, A. (2003). A new technique for the creation of a computerized composite skull model. J. Oral Maxillofac. Surg. *61*, 222–227.

65. Uechi, J., Okayama, M., Shibata, T., Muguruma, T., Hayashi, K., Endo, K., and Mizoguchi, I. (2006). A novel method for the 3-dimensional simulation of orthognathic surgery by using a multimodal image-fusion technique. Am. J. Orthod. Dentofacial Orthop. *130*, 786–798.

66. Swennen, G.R.J., Mommaerts, M.Y., Abeloos, J., De Clercq, C., Lamoral, P., Neyt, N., Casselman, J., and Schutyser, F. (2009). A cone-beam ct based technique to augment the 3d virtual skull model with a detailed dental surface. Int. J. Oral Maxillofac. Surg. *38*, 48–57.

67. Liu, J. (2023). The Dataset of "Deep Learning-Enabled 3d Multimodal Fusion of Cone-Beam Ct and Intraoral Mesh Scans for Clinically Applicable Tooth-Bone Reconstruction". https://doi.org/10.5281/zenodo.8027553.

68. Liu, J. (2023). The Code of "Deep Learning-Enabled 3d Multimodal Fusion of Cone-Beam Ct and Intraoral Mesh Scans for Clinically Applicable Tooth-Bone Reconstruction". https://doi.org/10.5281/zenodo.8027716.

69. Ye, M., Zhang, X., Yuen, P.C., and Chang, S.-F. (2019). Unsupervised embedding learning via invariant and spreading instance feature. In Proceedings of the IEEE/CVF Conference on Computer Vision and Pattern Recognition, pp. 6210–6219.

70. Shrivastava, A., Gupta, A., and Girshick, R. (2016). Training region-based object detectors with online hard example mining. In Proceedings of the IEEE conference on computer vision and pattern recognition, pp. 761–769.

71. Weir, T. (2017). Clear aligners in orthodontic treatment. Aust. Dent. J. *62*, 58–62.

72. Berman, M., Triki, A.R., and Blaschko, M.B. (2018). The lovász-softmax loss: A tractable surrogate for the optimization of the intersection-over-union

measure in neural networks. In Proceedings of the IEEE conference on computer vision and pattern recognition, pp. 4413–4421.

73. Pan, W., Lu, X., Gong, Y., Tang, W., Liu, J., He, Y., and Qiu, G. (2020). Hlo: Half-kernel laplacian operator for surface smoothing. Comput. Aided Des. *121*, 102807.

74. Bolles, R.C., and Fischler, M.A. (1981). A ransac-based approach to model fitting and its application to finding cylinders in range data. IJCAI *1981*, 637–643.

75. Liu, J. (2023). The Data of "deep Learning-Enabled 3d Multimodal Fusion of Cone-Beam Ct and Intraoral Mesh Scans for Clinically Applicable Tooth-Bone Reconstruction" (zenodo). https://doi.org/10.5281/zenodo.8078833.

76. Rusu, R.B., Blodow, N., and Beetz, M. (2009). Fast point feature histograms (fpfh) for 3d registration. In 2009 IEEE international conference on robotics and automation (IEEE), pp. 3212–3217.

77. Besl, P.J., and McKay, N.D. (1992). Method for registration of 3-d shapes. In Sensor fusion IV: control paradigms and data structures, *1611* (Spie), pp. 586–606.

78. Zhou, Q.-Y., Park, J., and Koltun, V. (2018). Open3d: A modern library for 3d data processing. Preprint at arXiv. https://doi.org/10.48550/arXiv.1801.09847.

Patterns, Volume *4*

Supplemental information

Deep learning-enabled 3D multimodal fusion of cone-beam CT and intraoral mesh scans for clinically applicable tooth-bone reconstruction

Jiaxiang Liu, Jin Hao, Hangzheng Lin, Wei Pan, Jianfei Yang, Yang Feng, Gaoang Wang, Jin Li, Zuolin Jin, Zhihe Zhao, and Zuozhu Liu

Patterns, Volume *4*

Supplemental information

Deep learning-enabled 3D multimodal fusion of cone-beam CT and intraoral mesh scans for clinically applicable tooth-bone reconstruction

Jiaxiang Liu, Jin Hao, Hangzheng Lin, Wei Pan, Jianfei Yang, Yang Feng, Gaoang Wang, Jin Li, Zuolin Jin, Zhihe Zhao, and Zuozhu Liu

Layers	Output size (Output name)	TSTNet
Input	h x w x 3	
Patch Embedding	h/4 x w/4 x 128	[Patch Partition → h/4 x w/4 x 48<br>Linear Embedding: 4x4, dim 128 Conv2d, stride 4 → LN]
Swin Block	h/4 x w/4 x 128	[LN → window size 7, dim 128, head 4 W-MSA → Residual connect<br>LN → MLP → Residual connect<br>LN → window size 7, dim 128, head 4 SW-MSA → Residual connect<br>LN → MLP → Residual connect]
Swin_stage2	h/8 x w/8 x 256	[Patch Merging [group feature map → h/8 x w/8 x 4 x 128<br>LN → dim 128 x 2 linear → h/8 x w/8 x 256]<br>window size 7, dim 256, head 8 Swin Block]
Swin_stage3	h/16 x w/16 x 512	[Patch Merging [group feature map → h/16 x w/16 x 4 x 256<br>LN → dim 256 x 2 linear → h/16 x w/16 x 512]<br>window size 7, dim 512, head 16 Swin Block] x 9
Swin_stage4	h/32 x w/32 x 1024<br>(pred seg)	[Patch Merging [group feature map → h/32 x w/32 x 4 x 512<br>LN → dim 512 x 2 linear → h/32 x w/32 x 1024]<br>window size 7, dim 1024, head 32 Swin Block]
TEC<br>[discard when infering]	(metric loss)	[Compute TP, FN, FP(GT labels, pred seg)<br>Compute category prototype(TP pixel) by EMA<br>Compute cos similarity(pixel, category prototype)<br>Error calibration [compute FP penalty term(FP pixel, anchor)<br>compute FN penalty term(FN pixel, anchor)]<br>Compute metric loss(cos similaity, penalty, compensation)]
FCN Head<br>[auxiliary head, discard when infering]	2 x h x w	[3 x 3, dim 256 Conv2d → SyncBN<br>3 x 3, dim 256 Conv2d → SyncBN<br>ratio 0.1 Dropout2d → 1x1, dim 2 Conv2d]
UperNet Head<br>[decode head]	2 x h x w	[head_input channels [128, 256, 512, 1024]<br>[channels 128 → 1 x 1, dim 512 Conv2d → SyncBN → out_128<br>channels 256 → 1 x 1, dim 512 Conv2d → SyncBN → out_256<br>channels 512 → 1 x 1, dim 512 Conv2d → SyncBN → out_512]<br>channels 1024 → [scale 1 AdaptiveAvgPool → 1 x 1, dim 512 Conv2d → SyncBN<br>scale 2 AdaptiveAvgPool → 1 x 1, dim 512 Conv2d → SyncBN<br>scale 3 AdaptiveAvgPool → 1 x 1, dim 512 Conv2d → SyncBN<br>scale 6 AdaptiveAvgPool → 1 x 1, dim 512 Conv2d → SyncBN]<br>→ concat along channel dim → 3 x 3, dim 512 Conv2d → SyncBN → out_1024<br>[out_128 → 3 x 3, dim 512 Conv2d → SyncBN<br>out_256 → 3 x 3, dim 512 Conv2d → SyncBN<br>out_512 → 3 x 3, dim 512 Conv2d → SyncBN]<br>SyncBN ← 3 x 3, dim 512 Conv2d ← concat along channel dim ←<br>→ ratio 0.1 Dropout2d → 1x1, dim 2 Conv2d]

Figure S1: **The details of TSTNet.**

Layers	Output Size (output name)	IOSNet
Input	b x n x 15	
Get_graph_feature	b x 30 x n x k	knn(k) ⟶ b x n x k<br>get_neighbor_feature ⟶ b x n x k x 15<br>offset_feature = neighbor_feature — center_point_feature ⟶ b x n x k x 15<br>concat offset_feature and center_point_feature along channel dimension ⟶ b x n x k x 30
Transformation Net	b x n x 15	1x1, 128 Conv2d ⟶ BatchNorm ⟶ Mish activation<br>1x1, 256 Conv2d ⟶ BatchNorm ⟶ Mish activation<br>max pool along k_neighbor dim ⟶ b x 256 x n<br>1x1, 1024 Conv1d ⟶ BatchNorm ⟶ Mish activation<br>max pool along n dim ⟶ b x 1024<br>512-d linear ⟶ Mish activation<br>256-d linear ⟶ Mish activation<br>225-d linear ⟶ reshape as b x15 x15(transform matrix)<br>input * transform matrix
EdgeConv_1	b x 64 x n (edgeconv1)	get_graph_feature<br>1x1, 128 Conv2d ⟶ BatchNorm ⟶ Mish activation<br>1x1, 128 Conv2d ⟶ BatchNorm ⟶ Mish activation<br>1x1, 64 Conv2d ⟶ BatchNorm ⟶ Mish activation<br>max pool along k_neighbor dim
EdgeConv_2	b x 64 x n (edgeconv2)	get_graph_feature<br>1x1, 64 Conv2d ⟶ BatchNorm ⟶ Mish activation<br>1x1, 64 Conv2d ⟶ BatchNorm ⟶ Mish activation<br>1x1, 64 Conv2d ⟶ BatchNorm ⟶ Mish activation<br>max pool along k_neighbor dim
EdgeConv_3	b x 64 x n (edgeconv3)	get_graph_feature<br>1x1, 64 Conv2d ⟶ BatchNorm ⟶ Mish activation<br>1x1, 64 Conv2d ⟶ BatchNorm ⟶ Mish activation<br>max pool along k_neighbor dim
Get_global_feature	b x 1024 (global_feature)	concat (edgeconv1, edgeconv2, edgeconv3)<br>1x1, 1024 Conv1d ⟶ BatchNorm ⟶ Mish activation<br>max pool along n dim
Category Vector	b x 2	
Add_category_vector	b x 1088 x n (new_global_fea)	1x1, 64 Conv1d ⟶ Mish activation ⟶ category_feature<br>concat(global_feature, category_feature)
Get_all_feature	b x 1280 x n	concat (new_global_fea, edgeconv1, edgeconv2, edgeconv3)
Segmentation head	b x 33 x n	1x1, 256 Conv1d ⟶ BatchNorm ⟶ Mish activation<br>dropout<br>1x1, 256 Conv1d ⟶ BatchNorm ⟶ Mish activation<br>dropout<br>1x1, 128 Conv1d ⟶ BatchNorm ⟶ Mish activation<br>1x1, 33 Conv1d

Figure S2: **The details of IOSNet.**

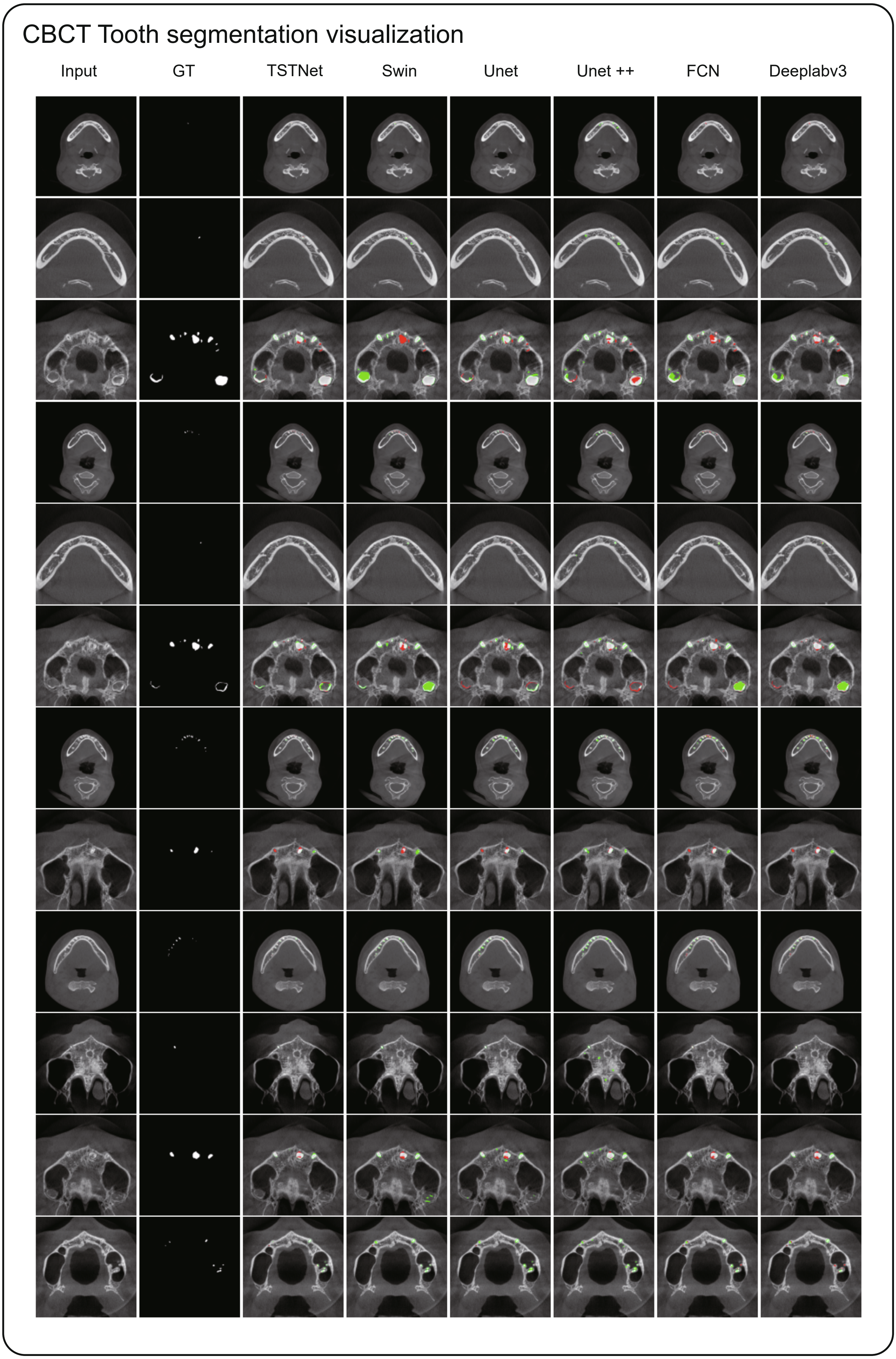

Figure S3: **More visualizations of tooth segmentation results.** TSTNet can accurately segment the pixels and greatly calibrate the FP and FN pixels. The results of TSTNet are the closest to the ground truth.

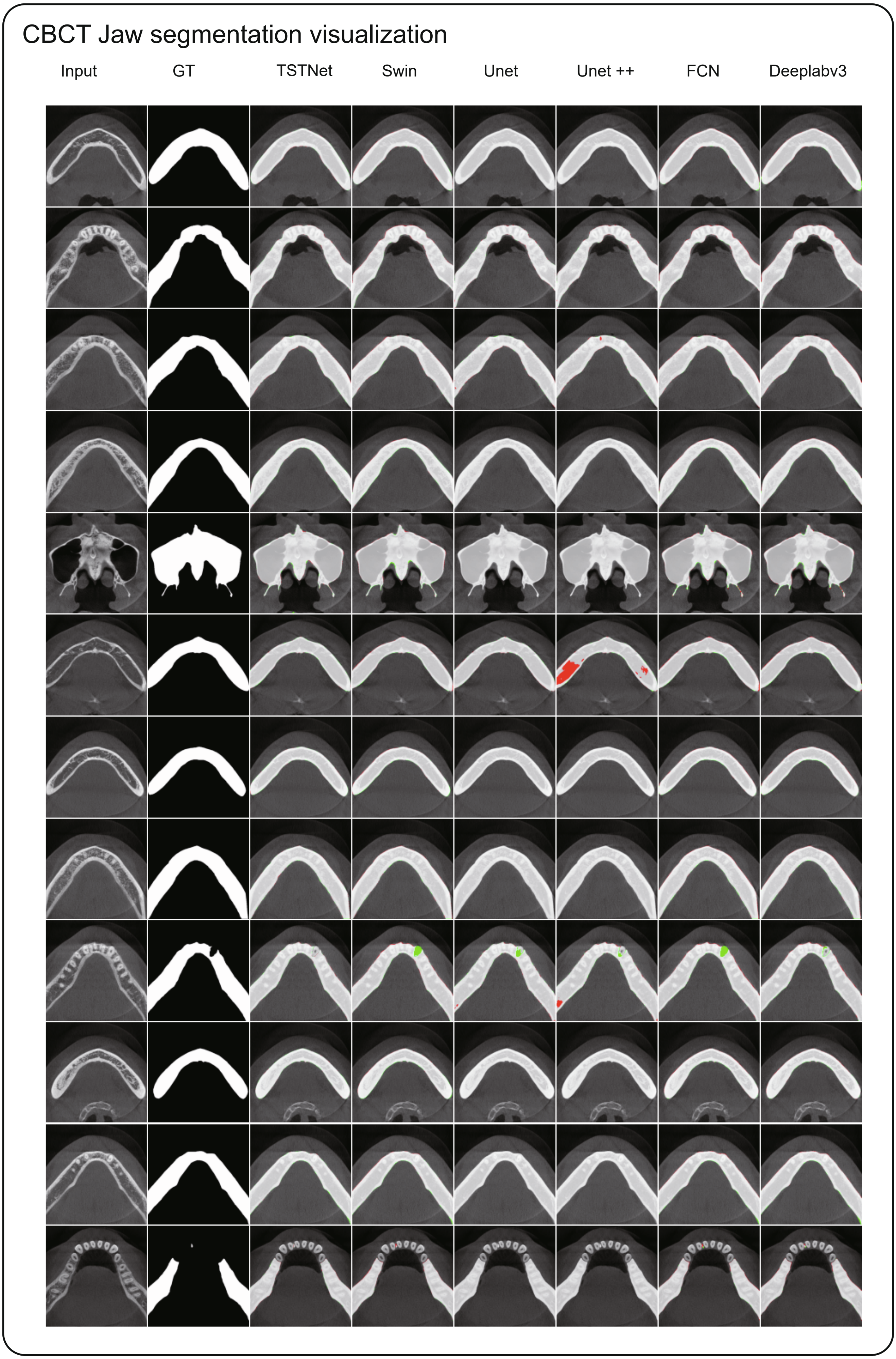

Figure S4: **More visualizations of jaw segmentation results.**

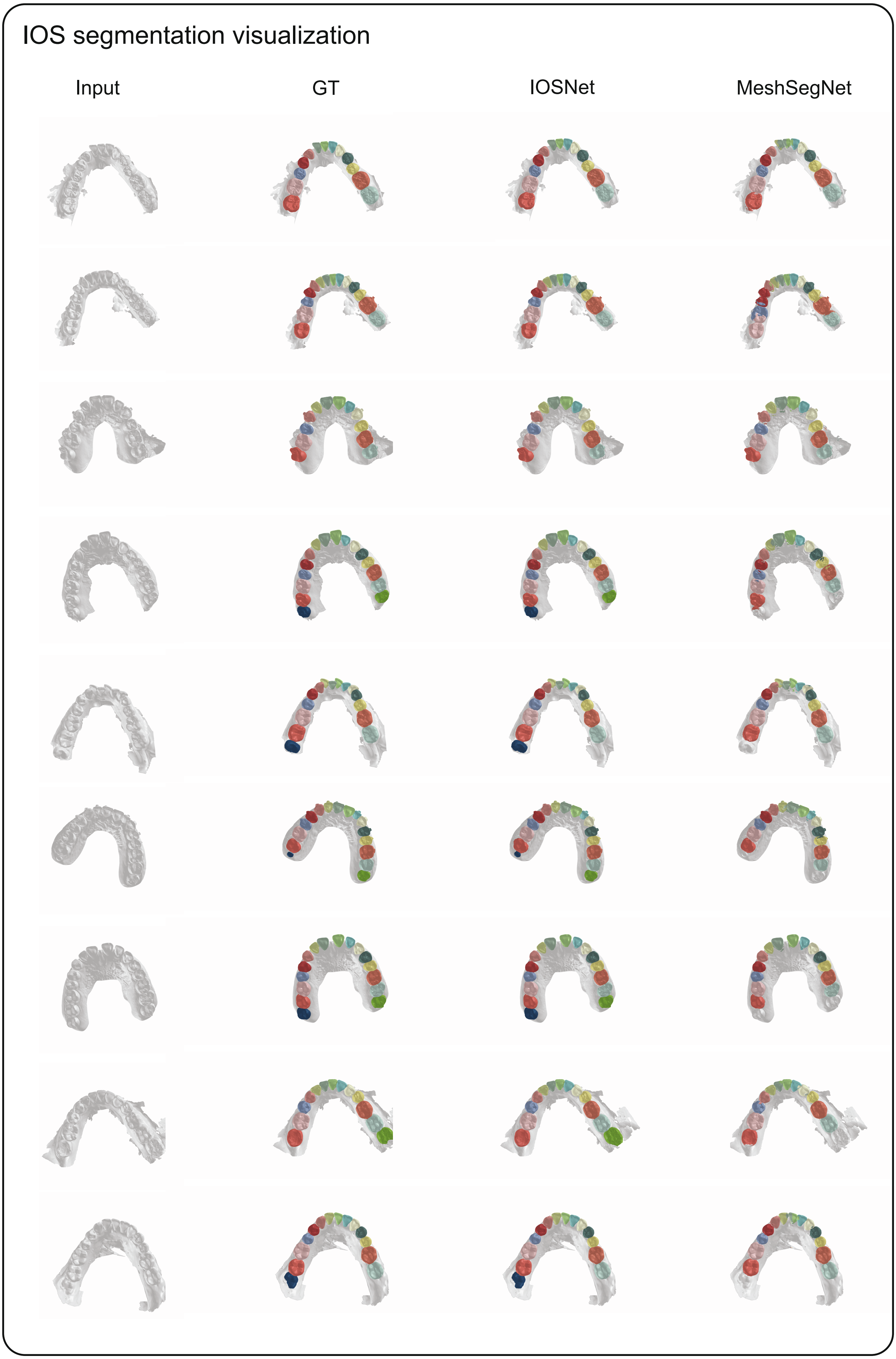

Figure S5**: More visualizations of IOSNet results.** IOSNet can get smoother and more accurate tooth segmentation results than MeshSegNet. In terms of the abnormal tooth numbers (the 1st row), our IOSNet can identify the incisors accurately.

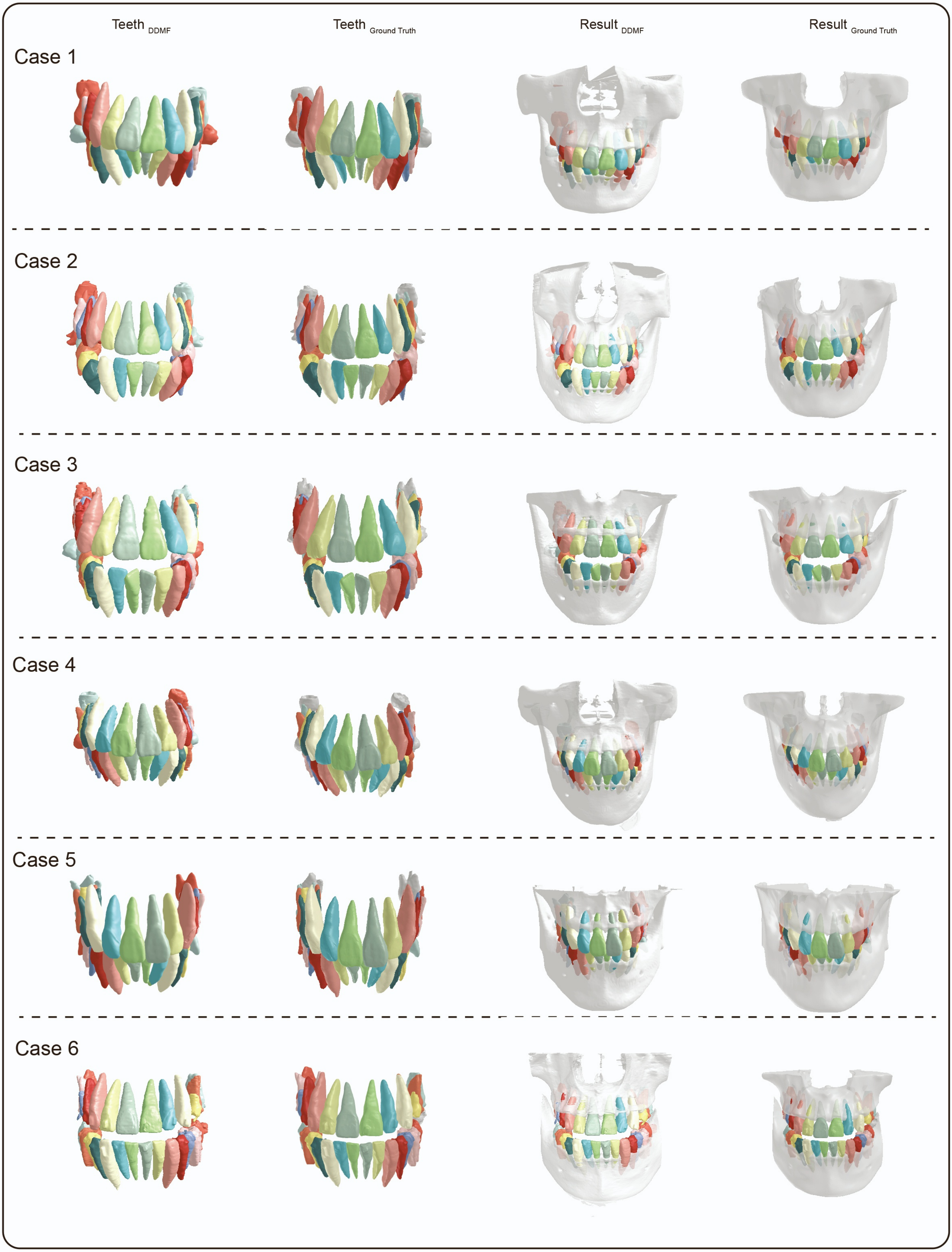

Figure S6: **More visualizations of DDMF results.** The quality of the DDMF result is approximate to the ground truth labeled by professional experts.

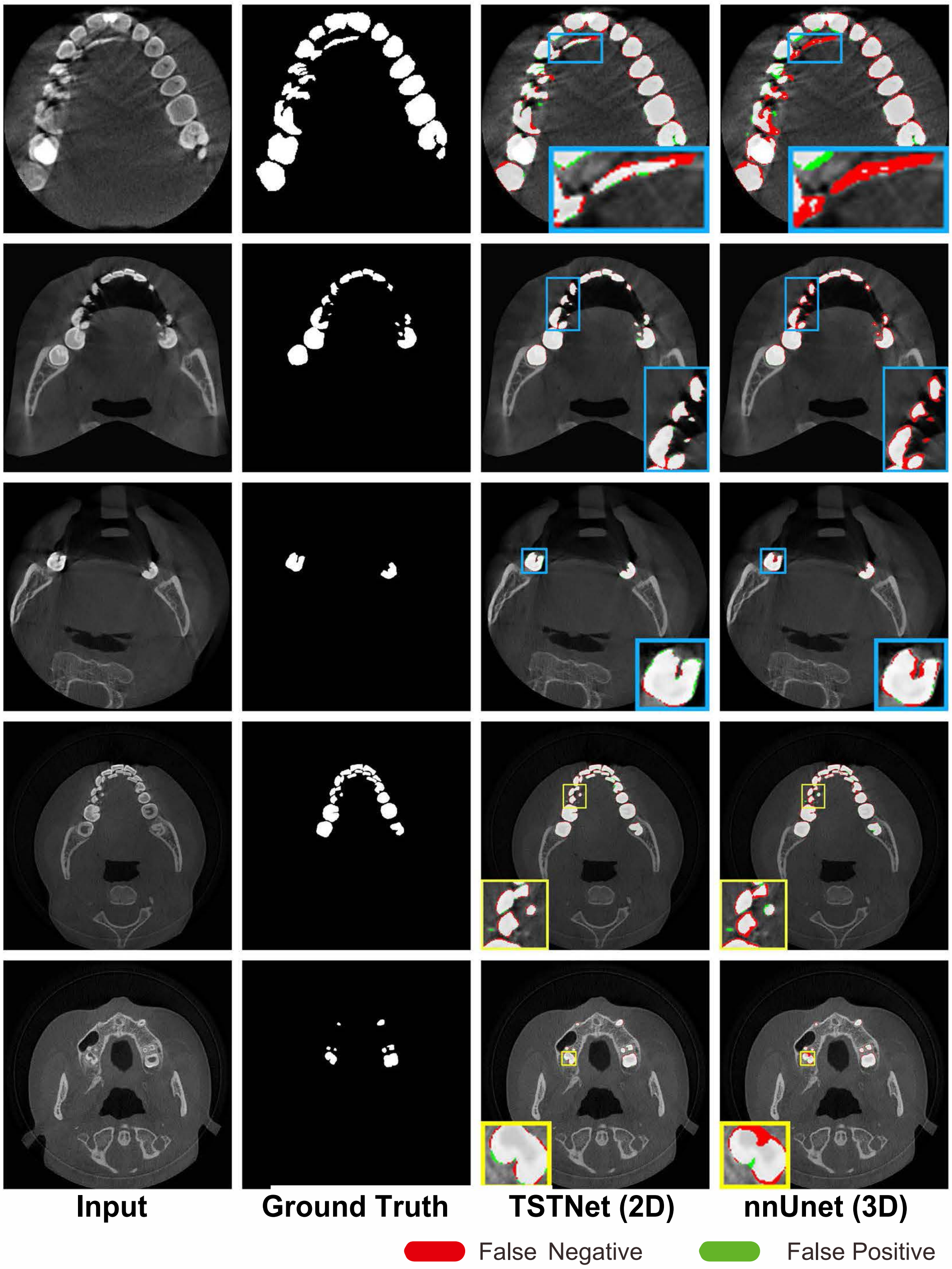

Figure S7: **Visualization of segmentation results for TSTNet and nnUnet.** We annotated 30 cases in 3D using 3D Slicer, and then trained nnUnet, a powerful backbone for many 3D segmentation tasks, with these 30 annotated cases. Visualizations demonstrate the superior performance of TSTNet, which commits fewer false positive and false negative errors than nnUnet.

Table S1: **Model complexity and clinical utility of CBCT segmentation (Tested on n=43 patients).** The number of parameters in TSTNet is approximately the same as the standard Swin transformer.

Models	Unet	Unet++	FCN	Deepla bv3	Swin	TSTNst (w/o aug)	TSTNet	TSTNst (multi-scale)	Human Experts
#Params(M)	29.62	34.95	49.48	68.10	121.17	121.43	121.43	121.43	-
Inf-T(s)	~0.072	~0.085	~0.076	~0.066	~0.071	~0.052	~0.479	~3.070	~600-900

#Params: number of parameters in the neural networks. Inf-T: end-to-end inference time of models. ~ means approximately. “w/o aug” denotes TSTNet with standard augmentation, i.e., without cropping and domain specific augmentation.
“multi-scale” denotes TSTNet with multi-scale inference.

Table S2: **Model Complexity and Clinical Utility of IOS Segmentation (Tested on n=100 patients).** It indicates that the number of parameters and the inference time of IOSNet are almost the same as the DC-Net. Compared with the baseline, although the IOSNet has more parameters, its inference speed is one order of magnitude faster than the baseline, and at least 25 times faster than the human experts, which shows that the IOSNet can achieve highly accurate segmentation at a faster speed. Therefore, our IOSNet has a promising future in clinical dentistry.

**Models**	**Baseline**	**DC-Net**	**IOSNet**	**Human Experts**
#Params(M)	0.4	1.5	1.5	-
Inf-T(s)	~321	~24	~24	~600-900

#Params: number of parameters in the neural networks. Inf-T: end-to-end inference time of models.

~ means approximately.

Table S3: **Evaluation of the registration between reconstructed CBCT meshes and IOSscans (Tested on 30 groups data).** Our registration method can achieve the best performance in the Registration of two modal data.

Method	Super4pc	AAICP	Ours
Fitness	0.1120	0.0573	**0.3100**
Inlier_rmse	0.0858	0.0866	**0.0823**
Correspondence_set size	7933.9	4009.5	**21270.9**
Success rate	13.30%	6.67%	**90%**

Fitness, which measures the overlapping area. The higher the better.

Inlier_rmse, which measures the RMSE of all inlier correspondences. The lower the better.

Table S4: **Tooth multimodal fusion Metrics of the proposed method (Tested on 479 teeth in 20 cases).**

Case	ASSD (mm)	CD (mm)	HD (mm)
Case01	0.14±0.04	0.17±0.02	0.35±0.22
Case02	0.18±0.02	0.21±0.02	0.40±0.05
Case03	0.18±0.03	0.21±0.03	0.39±0.08
Case04	0.14±0.02	0.17±0.02	0.31±0.06
Case05	0.25±0.07	0.27±0.07	0.51±0.10
Case06	0.11±0.02	0.14±0.02	0.21±0.04
Case07	0.17±0.04	0.19±0.05	0.35±0.13
Case08	0.14±0.02	0.17±0.02	0.30±0.09
Case09	0.14±0.03	0.17±0.02	0.32±0.07
Case10	0.10±0.02	0.14±0.02	0.23±0.06
Case11	0.12±0.06	0.15±0.06	0.28±0.16
Case12	0.11±0.05	0.14±0.04	0.26±0.12
Case13	0.10±0.02	0.14±0.02	0.23±0.05
Case14	0.13±0.04	0.16±0.03	0.31±0.08
Case15	0.21±0.09	0.23±0.07	0.48±0.23
Case16	0.15±0.08	0.18±0.06	0.34±0.17
Case17	0.12±0.03	0.15±0.03	0.26±0.07
Case18	0.15±0.02	0.17±0.02	0.30±0.06
Case19	0.22±0.03	0.24±0.03	0.57±0.13
Case20	0.21±0.09	0.24±0.07	0.59±0.55

ASSD: Average symmetric surface distance; HD: Housdoff distance; CD: Chamfer distance.

Table S5: **Segmentation results of TSTNet and nnUnet (Tested on 15 patients, which has 295 slices).** We annotated 30 cases in 3D using 3D Slicer, and then trained nnUnet, a powerful backbone for many 3D segmentation tasks, with these 30 annotated cases. TSTNet exhibits better slice-wise segmentation results than nnUnet.

**Model**	**IoU (%)**	**Dice (%)**	**Inf-T (s)**
nnUNet	85.02	91.90	~600
TSTNet	**88.77**	**94.05**	**~140**

IoU: intersection over union; Dice: Dice Coefficient. Bolden numbers indicate the best performance.

Inf-T: end-to-end inference time of models for a single patient.

Table S6: **CBCT information**

#CBCT scans	503 (more than 150,000 slices)
#Annotated slices	9,651
Age	19.53 ± 7.57 (mean ± std)
Male/Female	32.5%/67.5%
Pixel resolution of 2D CBCT slices	[260, 260] to [1000, 1000]

Imaging protocols

Voxel resolution	[0.3, 0.3, 0.3], [0.266, 0.266, 0.251], [0.2, 0.2, 0.2], [0.25, 0.25, 0.25], [0.226, 0.226, 0.226], [0.18, 0.18, 0.18], [0.35, 0.35, 0.35], [0.16, 0.16, 0.16], [0.246, 0.246, 0.245], [0.183, 0.183, 0.183], [0.227, 0.227, 0.227], [0.319, 0.319, 0.322], [0.5, 0.5, 0.5], [0.28, 0.28, 0.28], [0.337, 0.337, 0.3], [0.22, 0.22, 0.22], [0.15, 0.15, 0.15], [0.256, 0.256, 0.256], [0.32, 0.32, 0.269], [0.125, 0.125, 0.5]
CBCT machine	HYBPLUS, SS-X9010DPro-3DE, PHT-35LHS, HighRes3D, PAPAYA 3D, CS 9300 Select, NTVGiEVO, XG3D, SS-X10010DPlus, Point 800 3D Plus 8N, Alioth, SS-X9010DPro, K9500, RAYSCAN Alpha, skyView, ORTHOPHOS SL, CS 8100SC 3D, Point 3D Combi 500 Series, SS-X9010DMax

Supplemental Experimental Procedures

Dataset: The dataset includes 503 samples with both CBCT and IOS collected from hospitals and clinics in 25 provinces in China during 2018-2021. The 503 samples are also associated with ground truth 3D reconstructions with fused IOS tooth crowns and CBCT tooth bones, all annotated by a committee of human experts. The 503 patients are at age 19.53±7.57 years old, with 32.5% male and 67.5% female. Besides, we collected an extra 28,559 IOS meshes. The tooth segmentation network is trained with 5-fold cross validation each with 50 patients. The jaw segmentation network is trained with 840 slices and tested on 246 slices. The IOS segmentation network is evaluated with 200 IOS scans as in the baselines.

Data annotation for 3D CBCT scans is more time-consuming and expensive compared to annotating selected 2D slices, as indicated by existing literature [1,2] and our experience. It typically takes 150-240 minutes to get a coarse label for one 3D CBCT scan using interactive tools like 3DSlicer, and an additional 60-120 minutes to have experts double-check annotations. On the other hand, annotating one 2D CBCT scan with 15-20 slices takes 30-40 minutes, and experts can double-check annotations in 10 minutes with an observational error of 1-2 pixels. Due to this difference in annotation time, most 3D-based CBCT studies use small datasets or datasets with coarse annotations. Using 2D segmentation methods allows for leveraging large and high-quality datasets. Additionally, general 3D segmentation methods require more computational resources and exhibit larger inference latency[3]. For instance, segmenting one 3D CBCT scan using a standard 3D nnUnet [19] takes over 10 minutes with NVIDIA GTX 3090 GPUs. This long response time and GPU demand necessitate optimization of both algorithm design and engineering implementation to meet real-world clinical applications. Therefore, we use 2D segmentation instead of 3D to deal with CBCT segmentation.

We annotated 9,651 CBCT slices of the 503 patients for CBCT tooth segmentation, where we select 15-25 slices for each patient. Similarly, we annotated 1,066 images for CBCT jaw segmentation. The slices are selected to maximize the difference among slices, e.g., for a CBCT with 400 slices, we might start annotation at slice 100 where tooth apex are first scanned, and subsequently select in total 15-25 slices to annotate every 10 slices, avoiding annotating highly-correlated and highly-similar slices. By doing so we can get annotated slices that contain different anatomical information, which could help improve the robustness and generalization ability of our model. We choose the slices for the jaw annotation following the same procedure.

The annotation process for CBCT slices is three-stage. First, each slice is annotated by the senior undergraduates or master students majoring in stomatology. Second, the annotated slices are revised and corrected by a committee of experts who hold doctoral degrees in stomatology and at least 5 years clinical experience. Finally, several senior radiologists with more than 10 years experience are going to work with the expert to further refine the labels, and their consensus is regarded as the ground truth. The images are annotated with the Labelme software, while a small portion of them are also examined by the Mimic software. The details of the CBCT information can be found in Table S6.

The annotation process for the IOS dataset is as follows. Given an input mesh scan, the experts can use the software “Atreat Processor” to annotate each mesh face. For each patient, a junior technician with 1-5 years working experience in dentistry will first annotate the IOS scan, with the help of automic solutions such as DC-Net. Afterwards, the annotation will be examined by a senior technician with more than 5 years working experience and a dentist (usually an orthodontist).

The annotations of the fused tooth-bone meshes which are going to be used to compute the 3D fusion performance such as ASSD are also conducted by a committee of human experts assisted by CAD software. During annotation, the experts need to decide whether the annotation is satisfactory for future oral diagnosis and treatment planning. If all of them (usually three experts) agree that the annotation is satisfactory, we will take that annotation. Otherwise, the annotation needs to be improved until it is good enough for future clinical usage.

Data preprocessing: In training, this paper proposed a data augmentation strategy for CBCT data. Firstly, based on the prior and empirical statistics from the annotated masks, we cropped the lower 1/4 and right/top/left 1/10 in the original CBCT image to partially alleviate the class imbalance problem. The CBCT images are resized to random resolution within [2048, 2048] with a ratio range of is (0.5, 2.0), and randomly clipped to [512, 512]. Then, the random horizontal flipping probability of 0.5, and random photometric distortion are performed.

As for the IOS scans, we first uniformly sample 10,000 faces from the original mesh and regard each face center as a point to form a point cloud with 10,000 points. The features associated with each

point include three parts: 1. the position of the face, i.e., the 3D coordinate of the face center denoted as $h_c = (x_c, y_c, z_c) \in R^3$; 2. the 3-dimensional normal vector of the face surface $h_n \in R^3$; 3. the face shape feature $f_s \in R^9$. For each face with three vertices as $(x_i, y_i, z_i)$for i from 1 to 3, and a face center (x_c, y_c, z_c), the face shape feature is defined as $Concat(x_i - x_c, y_i - y_c, z_i - z_c)$ for i from 1 to 3. $Concat(\cdot)$ is the concatenate operation for vectors, leading to a 9-dimensional shape feature for each face. In consequence, the final output after data preprocessing for each IOS mesh scan is a point cloud with 10,000 points, each associated with a 15-dimensional feature vector $h = Concat(h_c, h_n, h_s) \in R^{15}$. This preprocessing procedure can be finished with a modern computer on-the-fly.

We apply data augmentation with four different perturbations to enlarge the IOS dataset. We independently sample 10,000 points every time for each perturbation. The perturbation is either random rotation by -10 to 10 degrees or random translation by -10 to 10 millimeters, along with one of the x, y, and z axes. The network is trained for 100 epochs. The model is evaluated on a validation set with 100 scans, and the model with the highest validation accuracy is selected for testing. During testing, we sample 40,000 points for each mesh, which will be segmented. For the rest points (i.e., faces), we choose five coordinate-based nearest neighbors from the 40,000 points, and the neighbor with the highest probability will determine the label for that point.

For CBCT jaw data, 840 CBCT images were for training, and 246 images were tested. In training, random horizontal flip and random scale are applied to augment the input image, random crop and padding are implemented to set the image size [512, 512].

TSTNet Details: The architecture details of TSTNet are shown in Figure S1. The OHEM and Lovasz loss are implemented as the reference [4], which assigns the weights (1:2) to background and tooth classes. The minimum keep pixels parameter in OHEM loss is 0.7.

IOSNet Details: The architecture details of IOSNet are shown in Figure S2.

Mesh reconstruction: The CBCT mesh is reconstructed from the CBCT segmentation results. The marching cubes algorithm is used to process CBCT image data to obtain the complete reconstructed mesh of all the CBCT teeth mesh [1]. Afterwards, the HLO is applied to smooth the surfaces to reduce the sharp creases on the surface caused by image pixels and marching cube algorithms [2]. Then, we got a satisfactory CBCT mesh.

Point Curvature Feature based Segmentation: In our method, the point curvature feature of a vertex is defined as the average of angles between normal vectors of all its neighbors, which is different from either mean curvature or Gaussian curvature, as defined in the Method. The point curvature feature is used to separate the maxilla and mandible, as well as delineate each tooth in the reconstructed 3D CBCT mesh.

We propose a new segmentation algorithm to separate the half jaws and each tooth using this novel point curvature feature. The intuition behind the curvature-based segmentation algorithm is that the angle between the normal vectors of adjacent points can capture the curvature of different scales by changing the level of neighbors. The algorithm is more or less like an "erosion-expansion" procedure. We first compute the point curvature feature for each vertex, and vertices with curvature in the top T percent would be removed from the mesh (erosion). Followed by a simple connected component analysis algorithm, the individual teeth can be separated. We will use 2nd order neighbors and $T = 20\%$ so that the main bodies of the teeth can be roughly separated. The general curvature-based segmentation algorithm is detailed in Algorithm 1 (Data S1). After the segmentation is applied, we will get both the unrefined teeth and the removed segments. After that, we extract every connected component from the remaining mesh as a single tooth. Small segments (experimentally, less than 2000 vertices) are discarded as noises. Then, we build the k-d tree for each estimated tooth, and we merge the removed point to its closest tooth. The details of the merge process are shown in Algorithm 2 (Data S2).

To better leverage the precision and efficiency of the k-d tree, we propose a multi-step approach during the merge process. We use T_{iter} steps for the merge process. In each step, we will merge the $1/T_{iter}$ removed points back to the teeth. Then we rebuild all the k-d trees based on the updated tooth points, and so on. The larger T_{iter}, the better merge performance. In the extreme case, we update the k-d tree after merging one point. Typically, we choose $T_{iter} = 5$ to balance the trade off between merging efficiency and performance. We take the minimum and the maximum z-axis coordinates of points as the reference, and classify each tooth to the maxilla or mandible by comparing the distance from the tooth centroid to the two references. As a result, the maxilla and mandible are separated and classified for registration in the next step. The maxilla and mandible segmentation algorithm is detailed in Algorithm 3 (Data S3).

Tooth crown replacement algorithm: When the registration IOS data is obtained, the next step is to fuse the CBCT with IOS scans to complete the integration of 3D crown and root data. The goal of this step is to replace the points and geometry corresponding to the tooth crown in CBCT with the IOS scan. We propose a tooth crown replacement algorithm, which consists of two steps: (1) Half-jaw fusion to get the fused half jaw, (2) Individual tooth fusion to get each fused individual tooth.

The half-jaw fusion algorithm is illustrated in Algorithm 4 (Data S4). We construct the KD-tree with the IOS data, and compute the distance from each point in the CBCT to the KD-tree. To remove the points corresponding to the CBCT crown, we remove points with top $d(d = 30\%)$ distance in the CBCT point cloud. However, some independent noise (including noise points and noise clusters) may be introduced in the mesh reconstruction process. Therefore, the outlier algorithm and the DBSCAN clustering algorithm are adopted for deleting those clusters and noise points [7]. The DBSCAN algorithm is a high-density-based clustering non-parametric algorithm that can delete some abnormal debris clusters with less than $k(k = 2000)$ point clouds. Finally, the oral scan is integrated with the remaining points in the CBCT, and Poisson reconstruction (Depth=8) is performed to generate the rough fusion results. Laplacian filtering algorithm (*Iter* = 6) is applied to obtain the final fusion result.

After we get the fused half jaw, we further integrate the tooth roots with the IOS mesh crown to get each tooth. By doing so we get each tooth with FDI notations. Specifically, we merge the remaining points in the CBCT to its corresponding IOS tooth crown by computing the Euclidean distance and integrating CBCT points to the nearest IOS tooth crown.

CBCT image segmentation metrics: To objectively evaluate the results obtained from different CBCT segmentation methods, four representative fusion quality metrics are adopted in this paper [8], using the default parameters provided in a corresponding study. The larger values of these metrics, the better segmentation results. More details regarding these metrics are as follows.

1) IoU

The Intersection over Union (IoU) is defined as the area of intersection between the predicted segmentation map A and the ground truth map B, divided by the area of the union between the two maps, and ranges between 0 and 1:

$$\mathrm{IoU} = J(A,B) = \frac{|A \cap B|}{|A \cup B|}.$$

2) Dice

The Dice coefficient, commonly used in medical image analysis, can be defined as twice the overlap area of the predicted and ground-truth maps divided by the total number of pixels:

$$\mathrm{Dice} = \frac{2|A \cap B|}{|A| + |B|}.$$

3) Recall / Precision

The Recall / Precision scores can be defined for each class, as well as at the aggregate level, as follows:

$$\mathrm{Recall} = \frac{\mathrm{TP}}{\mathrm{TP}+\mathrm{FN}}, \qquad \mathrm{Precision} = \frac{\mathrm{TP}}{\mathrm{TP}+\mathrm{FP}},$$

where TP refers to the true positive fraction, FP refers to the false positive fraction, and FN refers to the false negative fraction.

IOS mesh segmentation metrics

1) ACC_a

ACC_a means the average-area accuracy. Given a jaw mesh $M = \{m_i\}, i = 1\ to\ N$ with N faces, the ACC_a is defined as the ratio of the total area of correctly predicted faces to the whole faces :

$$ACC_a = \frac{\sum_{i=1}^{N} \Phi(y_i, \hat{y}_i) \cdot a_i}{\sum_{i=1}^{N} a_i},$$

$$\Phi(y_i, \hat{y}_i) = \begin{cases} 1, & if\ y_i = \hat{y}_i, \\ 0, & otherwise. \end{cases},$$

where a_i denotes the area for face m_i, $\hat{y}_i$ and y_i denotes the predicted label and the gold label of face m_i, respectively, and $\Phi(\cdot)$ is the indicator function.

2) ACC_f

ACC_f means the per-face accuracy. Similarly, given a jaw mesh $M = \{m_i\}, i = 1\ to\ N$ with *N* faces, the ACC_f is defined as the ratio of the number of correctly predicted faces to the whole faces:

$$ACC_f = \frac{1}{N} \sum_{i=1}^{N} \Phi(y_i, \hat{y}_i),$$

where the meanings of $y\hat{}_i$, y_i and $\Phi(\cdot)$ are the same as above.

Multimodal Fusion metrics: We evaluate the multimodal fusion result by computing the error between the results of DDMF and ground truth. We utilize three distance-based metrics to measure the geometric error, namely, Chamfer distance (CD) [9], average absolute distance (ASSD) [10], and Hausdorff distance (HD) [10]. The CD measures the mean distance between two sets of vertices. ASSD and HD compute the average and maximum distance between two sets of 10k sampled points from surface meshes.

The ASSD is defined as follows :

$$ASSD\left(S_R, S_A\right) = mean\left\{mean\left\{dist\left(a, S_R\right), a \in S_A\right\}, mean\left\{dist\left(r, S_A\right), r \in S_R\right\}\right\}.$$

*S*R and *S*A are the surfaces of objects of gold standard and algorithm segmentation, respectively, dist(a, *S*R) is the nearest Euclidean distance from a surface point a to the surface *S*R, and mean {•} is the arithmetical average operator.

The Hausdorff Distance (*HD*) between two finite point sets *A* and *B* is defined by:

$$HD(A, B) = \max(h(A, B), h(B, A)),$$

where *h(A,B)* is called the directed Hausdorff distance and given by

$$h(A, B) = \max_{a \in A} \min_{b \in B} ||a - b||,$$

where ||a−b|| is Euclidean distance between a and b.

The Chamfer distance between S_1, S_2 is defined as follow:

$$d_{CD}\left(S_1, S_2\right) = \sum_{x \in S_1} \min_{y \in S_2} \|x - y\|_2^2 + \sum_{y \in S_2} \min_{x \in S_1} \|x - y\|_2^2.$$

For each point, the d_{CD} finds the nearest neighbor in the other set and sums the squared distances up. Viewed as a function of point locations in S_1 and S_2, CD is continuous and piecewise smooth. The range search for each point is independent.

Comparison with Baselines

1) CBCT image segmentation

We introduce both state-of-the-art CNNs (Unet [11] , Unet++ [12] , FCN [13] , and DeepLabv3 [14]) and recently proposed transformer models(MedT [15], UctransNet [16], Swin transformer [17]). The backbone of DeepLabv3 and FCN is ResNet-50 [18]. The Swin Transformer is pre-trained with Imagenet-22k. DeepLabv3, FCN, and standard Swin Transformer are based on the public code at mmsegmentation [4], and the training hyper parameters are in mmsegmentation by default. The implementations of Swin, MedT, UCTransNet, UNet, UNet++ are based on their original source codes. The training is conducted with 4 Nvidia RTX 3090 GPUs and Pytorch 1.8.0 for comparison experiments, and the training procedure lasts for 160K iterations.

Supplemental References

1. Xie, Y., Zhang, J., Xia, Y., and Wu, Q. (2022). UniMiSS: Universal Medical Self-supervised Learning via Breaking Dimensionality Barrier. In Computer Vision–ECCV 2022: 17th European Conference, Tel Aviv, Israel, October 23–27, 2022, Proceedings, Part XXI (Springer), pp. 558–575.
2. Cui, Z., Fang, Y., Mei, L., Zhang, B., Yu, B., Liu, J., Jiang, C., Sun, Y., Ma, L., and Huang, J. (2022). A fully automatic AI system for tooth and alveolar bone segmentation from cone-beam CT images. Nat. Commun. *13*, 1–11.
3. Zhang, Y., Liao, Q., Ding, L., and Zhang, J. (2022). Bridging 2D and 3D segmentation networks for computation-efficient volumetric medical image segmentation: An empirical study of 2.5 D solutions. Comput. Med. Imaging Graph. *99*, 102088.
4. Xu, J., Chen, K., and Lin, D. (2020). MMSegmenation.
5. Rusu, R.B., Blodow, N., and Beetz, M. (2009). Fast point feature histograms (FPFH) for 3D registration. In 2009 IEEE international conference on robotics and automation (IEEE), pp. 3212–3217.
6. Besl, P.J., and McKay, N.D. (1992). Method for registration of 3-D shapes. In Sensor fusion IV: control paradigms and data structures (Spie), pp. 586–606.
7. Zhou, Q.-Y., Park, J., and Koltun, V. (2018). Open3D: A modern library for 3D data processing. ArXiv Prepr. ArXiv180109847.
8. Minaee, S., Boykov, Y.Y., Porikli, F., Plaza, A.J., Kehtarnavaz, N., and Terzopoulos, D. (2021). Image segmentation using deep learning: A survey. IEEE Trans. Pattern Anal. Mach. Intell.
9. Fan, H., Su, H., and Guibas, L.J. (2017). A point set generation network for 3d object reconstruction from a single image. In Proceedings of the IEEE conference on computer vision and pattern recognition, pp. 605–613.
10. Cruz, R.S., Lebrat, L., Bourgeat, P., Fookes, C., Fripp, J., and Salvado, O. (2021). Deepcsr: A 3d deep learning approach for cortical surface reconstruction. In Proceedings of the IEEE/CVF Winter Conference on Applications of Computer Vision, pp. 806–815.
11. Ronneberger, O., Fischer, P., and Brox, T. (2015). U-net: Convolutional networks for biomedical image segmentation. In International Conference on Medical image computing and computer-assisted intervention (Springer), pp. 234–241.
12. Zhou, Z., Siddiquee, M.M.R., Tajbakhsh, N., and Liang, J. (2019). Unet++: Redesigning skip connections to exploit multiscale features in image segmentation. IEEE Trans. Med. Imaging *39*, 1856–1867.
13. Long, J., Shelhamer, E., and Darrell, T. (2015). Fully convolutional networks for semantic segmentation. In Proceedings of the IEEE conference on computer vision and pattern recognition, pp. 3431–3440.

14. Chen, L.-C., Papandreou, G., Schroff, F., and Adam, H. (2017). Rethinking atrous convolution for semantic image segmentation. ArXiv Prepr. ArXiv170605587.
15. Valanarasu, J.M.J., Oza, P., Hacihaliloglu, I., and Patel, V.M. (2021). Medical transformer: Gated axial-attention for medical image segmentation. In International Conference on Medical Image Computing and Computer-Assisted Intervention (Springer), pp. 36–46.
16. Wang, H., Cao, P., Wang, J., and Zaiane, O.R. (2022). Uctransnet: rethinking the skip connections in u-net from a channel-wise perspective with transformer. In Proceedings of the AAAI Conference on Artificial Intelligence, pp. 2441–2449.
17. Liu, Z., Lin, Y., Cao, Y., Hu, H., Wei, Y., Zhang, Z., Lin, S., and Guo, B. (2021). Swin transformer: Hierarchical vision transformer using shifted windows. In Proceedings of the IEEE/CVF International Conference on Computer Vision, pp. 10012–10022.
18. He, K., Zhang, X., Ren, S., and Sun, J. (2016). Identity mappings in deep residual networks. In European conference on computer vision (Springer), pp. 630–645.
19. Isensee, F., Jaeger, P.F., Kohl, S.A., Petersen, J., and Maier-Hein, K.H. (2021). nnU-Net: a self-configuring method for deep learning-based biomedical image segmentation. Nat. Methods *18*, 203–211.
